# CSF3R-AS promotes hepatocellular carcinoma progression and sorafenib resistance through the CSF3R/JAK2/STAT3 positive feedback loop

**DOI:** 10.1038/s41419-025-07558-4

**Published:** 2025-03-28

**Authors:** Ziyang Feng, Yan Gao, Changjing Cai, Jun Tan, Ping Liu, Yihong Chen, Gongping Deng, Yanhong Ouyang, Xuewen Liu, Ke Cao, Shan Zeng, Ying Han, Xiangying Deng, Hong Shen

**Affiliations:** 1 Department of Oncology, Xiangya Hospital, Central South University, Changsha, Hunan 410008 China; 2 Department of Oncology, The Third Xiangya Hospital, Central South University, Changsha, Hunan 410008 China; 3 Postdoctoral Station of Medical Aspects of Specific Environments, The Third Xiangya Hospital, Central South University, Changsha, Hunan 410008 China; 4 Department of Neurosurgery, Xiangya Hospital, Central South University, Changsha, Hunan 410008 China; 5 Department of Emergency, Hainan General Hospital, Hainan Affiliated Hospital of Hainan Medical University, Hainan, 570311 China; 6 National Clinical Research Center for Geriatric Disorders, Xiangya Hospital, Central South University, Changsha, Hunan 410008 China

**Keywords:** Oncogenes, Liver cancer

## Abstract

Antisense circular RNA is a special type of circular RNA that is derived from the antisense complementary strand of parental mRNA. However, the function of antisense circRNA in hepatocellular carcinoma (HCC) is still unclear. Here, we reported that CSF3R-AS was upregulated in HCC and correlated with a poor prognosis. CSF3R-AS promoted the proliferation, angiogenesis, and metastasis of HCC, and inhibited apoptosis. Mechanistically, CSF3R-AS has a 180-base complementary pairing sequence with its parental mRNA CSF3R, which can directly bind to CSF3R and recruit RBMS3 to stabilize its parental mRNA, and finally activate JAK2/STAT3 signaling pathway. Interestingly, STAT3 can act as a transcription factor of CSF3R-AS, which means that there is a CSF3R-AS/CSF3R/JAK2/STAT3 positive feedback loop in HCC. Finally, the CSF3R-AS/CSF3R/JAK2/STAT3 positive feedback loop was also activated in HCC sorafenib-resistant cells, and blocking this loop was expected to improve the sensitivity of HCC to sorafenib. These findings suggested that the CSF3R-AS/CSF3R/JAK2/STAT3 positive feedback loop could promote HCC progression and sorafenib resistance. Blocking this loop is expected to provide new research directions and therapy targets for HCC.

## Introduction

Currently, malignant tumors are a major cause of disease-related mortality in humans. Among all malignant tumors, liver cancer has the second highest mortality rate [1]. The primary causes or etiology of liver cancer include viral infection, aflatoxin, nitrosamines, alcohol, cirrhosis, and heredity [2]. According to the histologic classification of liver cancer, it can be divided into three types: Hepatocellular carcinoma (HCC), hepatobiliary epithelial carcinoma, and mixed hepatocellular carcinoma [3–5]. HCC is the most common type of liver cancer, accounting for more than 90% of all liver cancers [6–8]. In recent years, significant advancements have been made in the treatment of HCC, including surgery, chemotherapy, radiotherapy, interventional therapy, immunotherapy, targeted therapy, and the integration of traditional Chinese medicine approaches, which have improved the prognosis of HCC to a certain extent [9, 10]. However, due to the fact that many patients were diagnosed at the advanced stage of the disease, and drug-resistance is a common issue among the majority of patients, the five-year overall survival rate (OS) of HCC remains relatively low at just 18% [11]. Therefore, it is of great significance to explore new therapeutic targets and drug resistance mechanisms for HCC.

Non-coding RNA (ncRNA) is a class of RNAs that cannot encode polypeptides and proteins, mainly including: long non-coding RNA (lncRNA), microRNA (miRNA), circularRNA (circRNA), small nuclear RNA (snRNA), small nucleolar RNA (snoRNA), and so on [12–14]. Non-coding RNAs have been regarded as meaningless fragments produced during mRNA shearing, but in recent years, researchers have found that non-coding RNAs are involved in the regulation of various important physiological activities, especially the progression of malignant tumors. Among them, circRNAs are a special class of non-coding RNAs that are more resistant to the digestion of nucleic acid exonucleases [15–17]. It has been found that the main functions of circRNAs include: (1) MiRNAs sponges; (2) Binding to proteins; (3) Translating proteins; (4) Participating in the regulation of alternative splicing; (5) Regulation of parental gene expression [18–20]. Among them, antisense circular RNAs are a more specialized class of circRNAs that are derived from the antisense complementary strand of parental mRNAs, and may be involved in regulating the expression of parental mRNAs at the transcriptional, splicing, and translational levels. Current studies have shown that the main functions of antisense RNAs include: (1) Binding to DNA and affecting DNA transcription; (2) Binding directly to mRNA and affecting mRNA translation; (3) Recruiting RNA-binding proteins to increase mRNA stability [21–23]. For example, Jian Ma found that circSCRIB inhibits the splicing and translation of its parental mRNA, which in turn promotes the proliferation, invasion, and metastasis of breast cancer [24]. So far, the role and function of antisense circRNAs in HCC have never been reported, and their specific mechanisms still need to be further investigated.

Colony Stimulating Factor 3 Receptor (CSF3R) is a member of the class I cytokine receptor superfamily, which is widely involved in the regulation of granulocyte hematopoietic cell proliferation and differentiation [25–27]. In recent years, more and more studies have shown that CSF3R may also play a key role in the progression of solid tumors. For example, Chakraborty et al. found that CSF3R activation promoted the proliferation of bladder cancer cells in vitro, and in vivo experiments also showed that CSF3R-positive cells had significantly larger tumor volumes than negative cells [28]. Sorafenib is the earliest targeted drug approved by the European Medicines Agency for the treatment of HCC. It is a multi-targeted kinase inhibitor and significantly inhibits tumor cell proliferation and angiogenesis [29–31]. Numerous studies have shown that sorafenib can achieve good efficacy when used to treat patients with advanced HCC [32, 33]. However, relevant studies have also shown that patients treated with sorafenib tend to become drug resistant within six months, and the emergence of drug resistance greatly limits the clinical efficacy of sorafenib [34]. Current studies suggest that several signaling pathways are involved in the acquired resistance of sorafenib, including (1) the PI3K/AKT pathway, (2) the JAK2/STAT3 pathway, and (3) the RAS/RAF/MEK/ERK pathway [35–37]. For example, Su-Chuan Lai found that activation of the IL6/STAT3 pathway could induce resistance of HCC to sorafenib by up-regulating the expression of DNMT3b and OCT4 [38]. So far, the specific role of CSF3R in HCC progression and sorafenib resistance has not been reported.

In our study, we found that CSF3R-AS, as an antisense circRNA, was significantly upregulated and associated with a poor prognosis of HCC. Mechanistically, we found that CSF3R-AS had a 180-base complementary pairing site with its parental mRNA CSF3R, and thus can directly bind to CSF3R, while CSF3R-AS can act as an intermediate scaffold molecule to recruit RBMS3, which in turn can stabilize CSF3R. We also found that there is a CSF3R-AS/CSF3R/JAK2/STAT3 positive feedback loop in HCC, which plays important roles in promoting HCC progression and sorafenib resistance, and the blockade of this loop is expected to provide a novel therapeutic target for HCC.

## Results

### CSF3R-AS is up-regulated and correlates with a poor prognosis in HCC

**Fig. 1:**
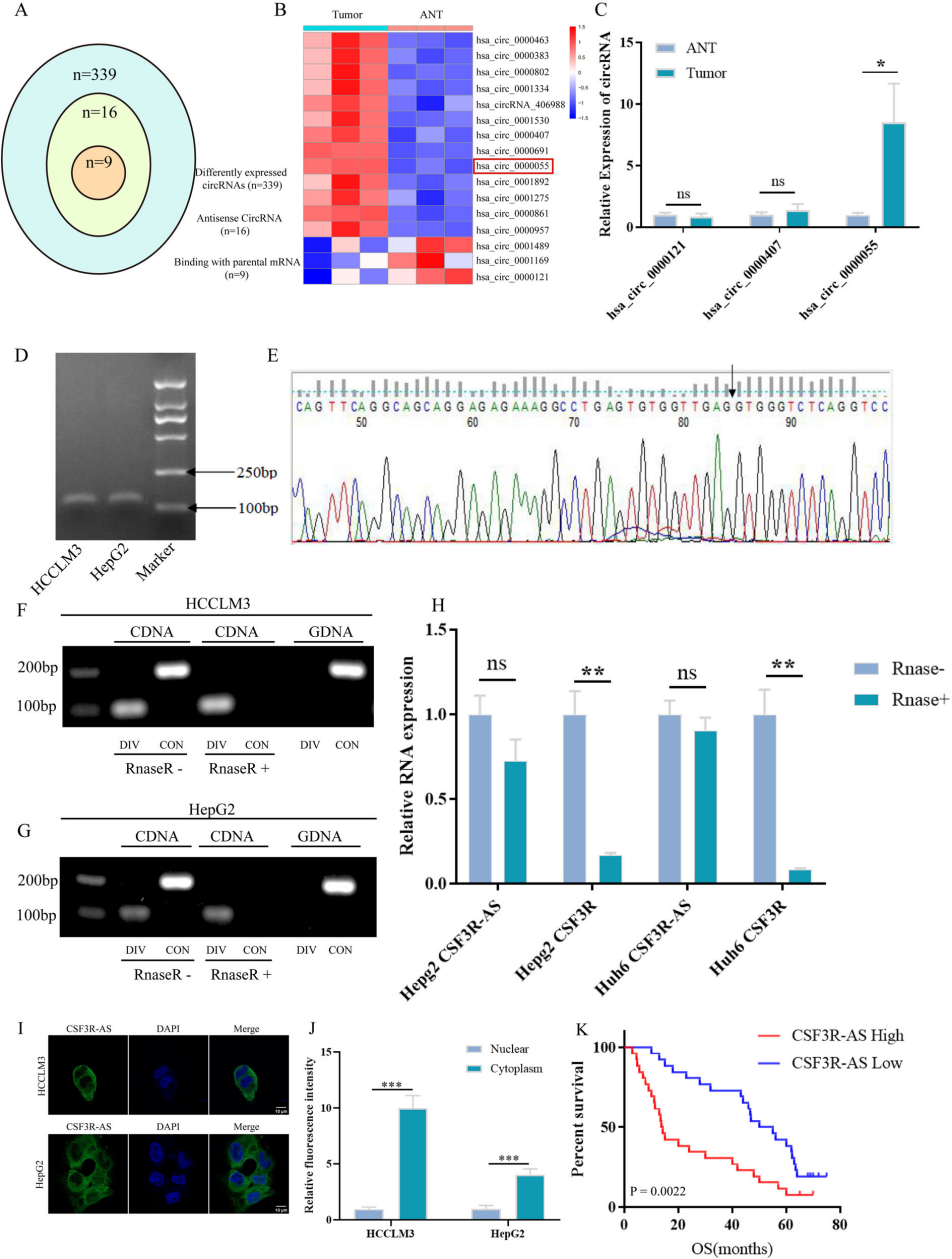
The identification and characterization of CSF3R-AS in HCC. **A** There were 339 differently expressed circRNAs in HCC, including 16 antisense circRNAs. 9 of them had the reverse complementary sequence with their parental mRNA. **B** Among the 16 antisense circRNAs, 13 of them were up-regulated and 3 of them were down-regulated. **C** The qRT-PCR results suggest that only hsa_circ_0000055 was up-regulated in HCC. **D** The agarose gel electrophoresis suggests that the length of the PCR product was consistent with expectation. **E** Sanger sequencing to validate the junction site. **F**–**G** The divergent primer can be successfully used to amplify the template in CDNA after the treatment with Rnase R, but the convergent primer could not. The divergent primer could not amplify the template using GDNA. **H** CSF3R-AS was more resistant to Rnase R. **I**–**J** CSF3R-AS was mainly located in the cytoplasm of HCC cells. **K** HCC patients with higher CSF3R-AS expression had worse OS. (ANT: adjacent noncancerous tissue, ns: none significance, * represents *P* < 0.05, ** represents *P* < 0.01).

To explore the role of antisense circRNAs in the development of HCC, we performed circRNAs sequencing in three HCC tumor tissues and paired adjacent noncancerous tissues. The results obtained suggested that there were 339 differently expressed circRNAs, including 16 antisense circRNAs (Fig. 1A). Among the 16 antisense circRNAs, 13 of them were up-regulated while 3 of them were down-regulated (Fig. 1B). Furthermore, we analyzed the 16 antisense circRNAs sequence and found that 9 antisense circRNAs had the reverse complementary sequence with their parental mRNA, including hsa_circ_0000121, hsa_circ_0000802, hsa_circ_0001334, hsa_circ_0001530, hsa_circ_0000407, hsa_circ_0000055, hsa_circ_0001892, hsa_circ_0000957 and hsa_circ_0000691. Next, we tried to design the divergent primer to detect these circRNAs, but only three primers were successfully designed, including hsa_circ_0000121, hsa_circ_0000407, and hsa_circ_0000055. Then, we detected the expression of these circRNAs in HCC tissues via qRT-PCR. The results obtained showed that only hsa_circ_0000055 was significantly up-regulated in HCC (Fig. 1C). The parental mRNA of hsa_circ_0000055 was CSF3R, so we named hsa_circ_0000055 as CSF3R-AS and decided to study it further.

To further verify the circular structure of CSF3R-AS, the PCR product was validated via agarose gel electrophoresis and sanger sequencing (Fig. 1D, E). Next, Rnase R was used to treat the RNA of HepG2 and HCCLM3 cells. The results of agarose gel electrophoresis suggested that a divergent primer can be successfully used to amplify the template in C DNA after the treat of Rnase R, but a convergent primer cannot be used to amplify the template (Fig. 1F, G). Besides, a divergent primer cannot amplify the template using G DNA (Fig. 1F, G). Similarly, the results of qRT-PCR suggested that CSF3R-AS was more resistant to Rnase R compared to the liner transcript (Fig. 1H). Finally, the result of FISH showed that CSF3R-AS was mainly located in the cytoplasm of HCC cells (Fig. 1I, J).

Next, we analyzed the correlation between CSF3R-AS expression and HCC clinical pathological parameters. Kaplan-Meier analysis revealed that HCC patients with higher CSF3R-AS expression had worse OS (Fig. 1K). Besides, the expression of CSF3R-AS was correlated with tumor size, vascular invasion and TNM stage, and higher expression of CSF3R-AS represented larger tumor size, worse vascular invasion and higher TNM stage (Table S1). Then, the univariate cox regression analyses suggested that tumor size, differention, vascular invasion, TNM stage and CSF3R-AS were related to HCC patients’s OS (Table S2). Finally, we further performed multivariate cox regression analysis, the results showed that tumor size, differention, TNM stage and CSF3R-AS may function as an independent risk factor for HCC patients prognosis (Table S3).

### CSF3R-AS promotes HCC progression in vitro and in vivo

**Fig. 2:**
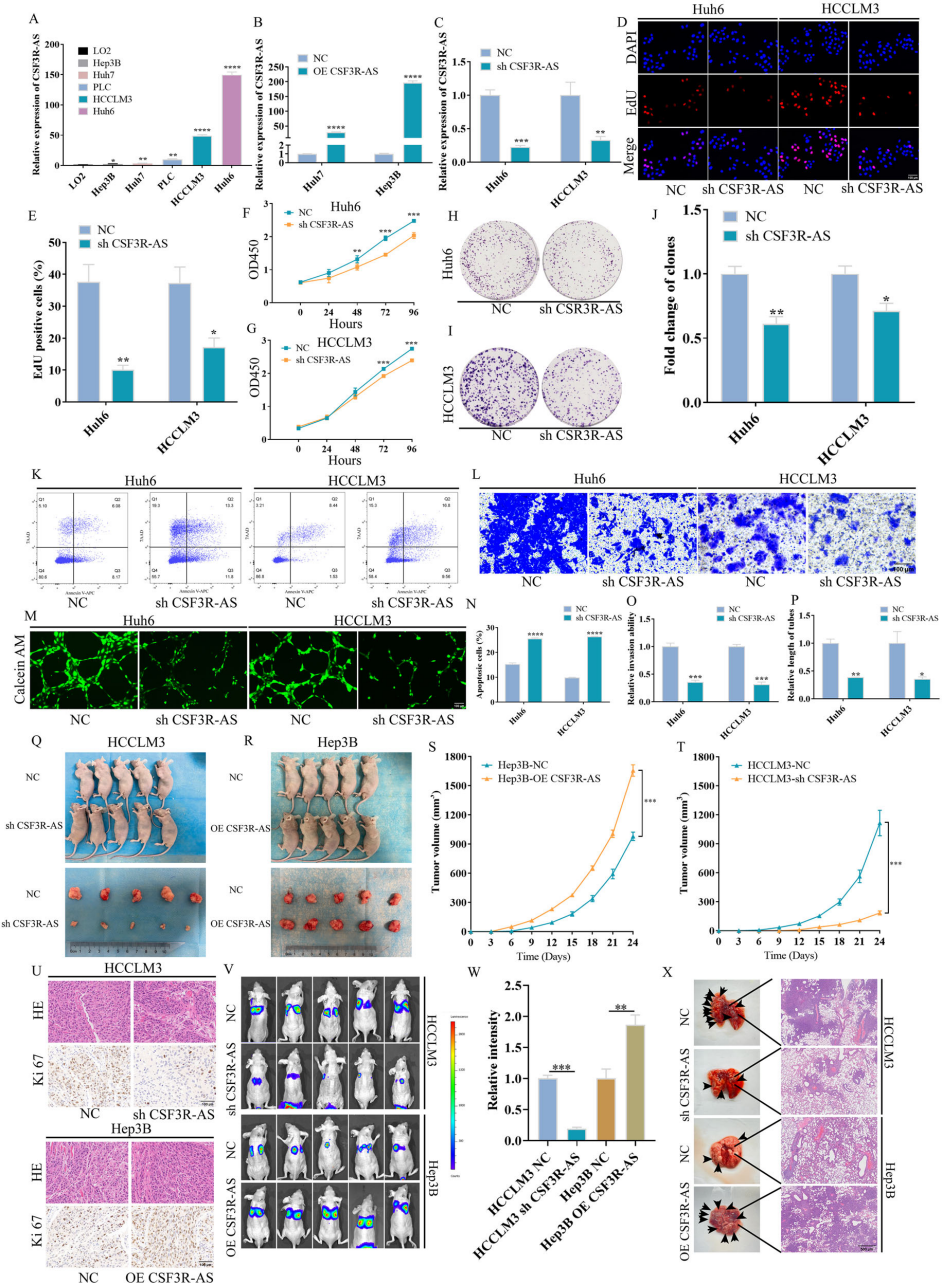
CSF3R-AS promoted HCC progression in vitro and in vivo. **A** The expression of CSF3R-AS in Huh6, HCCLM3, PLC, Huh7, Hep3B, and normal liver epithelial cells LO2. **B**, **C** The overexpression and knock down efficiency of CSF3R-AS. **D**–**J** The results of EdU, CCK8, and clone formation suggest that the knock down of CSF3R-AS repressed cell proliferation. **K** The knock down of CSF3R-AS promoted apoptosis. **L**–**M** The knock down of CSF3R-AS suppressed cell invasion and angiogenesis. **N**–**P** The quantitative statistics of (**K**–**M**). **Q**, **R** Representative images of subcutaneous tumor tissues (*n* = 5). **S**, **T** The tumor volume growth curves of subcutaneous tumors in nude mice. **U** The staining of HE and Ki67 of subcutaneous tumors. **V** Representative in vivo imaging of a lung metastasis model (*n* = 5). **W** The quantitative statistics of (**V**). **X** Representative HE staining of lung sections in (**V**). (* represents *P* < 0.05, ** represents *P* < 0.01, *** represents *P* < 0.001, **** represents *P* < 0.0001).

Next, we detected the expression of CSF3R-AS in Huh6, HCCLM3, PLC, Huh7, Hep3B, and normal liver epithelial cells LO2 (Fig. 2A). According to the expression of CSF3R-AS, CSF3R-AS was knocked down in Huh6 and HCCLM3 cells and overexpressed in Huh7 and Hep3B cells (Fig. 2B, C). The results of EdU, CCK8, and clone formation suggested that the knock down of CSF3R-AS repressed cell proliferation, whereas the overexpression of CSF3R-AS exerted opposite effects (Fig. 2D–J, Figure S1A–G). Flow cytometry showed that the knock down of CSF3R-AS promoted apoptosis (Fig. 2K and N), but the overexpression of CSF3R-AS repressed apoptosis (Figure S2A and D). Transwell and tube formation assay revealed that the knock down of CSF3R-AS suppressed cell invasion and angiogenesis (Fig. 2L-M and O-P), while the opposite were observed after the overexpression of CSF3R-AS (Figure S2B, C and E-F).

To explore the function of CSF3R-AS in vivo, we established xenograft mouse models by subcutaneously injecting CSF3R-AS overexpressed cells or knock down cells. As shown in Fig. 2Q–T, the tumor of the CSF3R-AS knock down group grew slower than that of the control group. Similarly, the tumor grew faster after the overexpression of CSF3R-AS (Fig. 2Q–T). Besides, the results of IHC suggested that the knock down of CSF3R-AS reduced the expression of Ki67, while the opposite were observed after the overexpression of CSF3R-AS (Fig. 2U). Furthermore, we analyzed whether CSF3R-AS promoted the distant metastasis of HCC via tail vein injection of HCC cells. Figure 2V–X suggests that the knock down of CSF3R-AS significantly suppressed lung metastasis of HCC cells, whereas the overexpression of CSF3R-AS promoted lung metastasis of HCC cells.

### CSF3R-AS increases the stability of CSF3R mRNA via recruiting RBMS3 protein

**Fig. 3:**
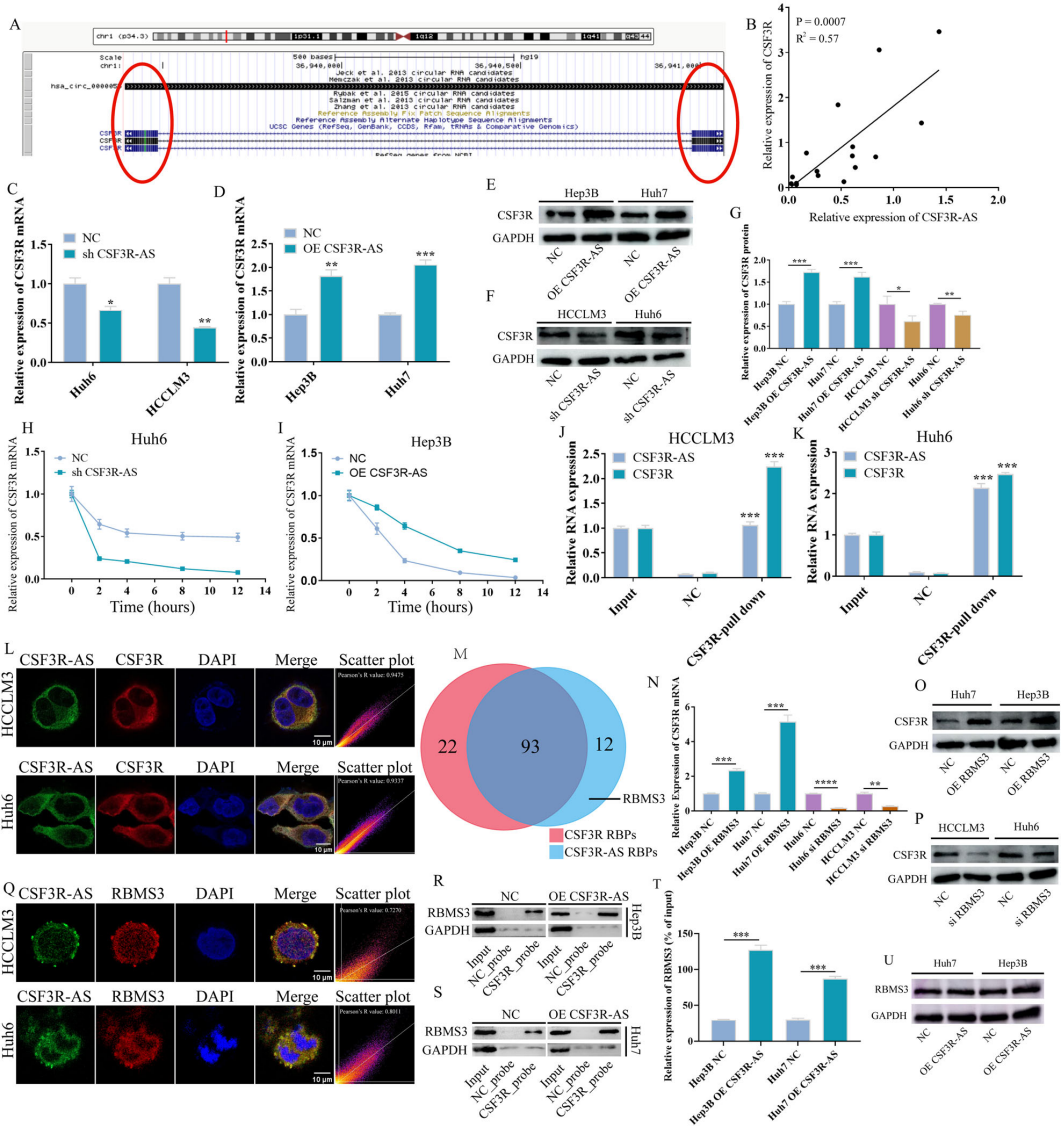
CSF3R-AS increased the stability of CSF3R mRNA via recruiting RBMS3 protein. **A** The 180 reverse complementary pairing bps between CSF3R-AS and CSF3R mRNA. **B** The correlation between CSF3R-AS and CSF3R mRNA. **C**–**G** CSF3R-AS affected the expression of CSF3R mRNA and protein. **H**–**I** CSF3R-AS increased the stability of CSF3R mRNA. **J**–**K** CSF3R probe group can enrich more CSF3R-AS than the NC probe group. **L** The colocalization between CSF3R-AS and CSF3R mRNA. **M** The RBPs that interacting with CSF3R-AS and CSF3R. **N**–**P** RBMS3 affected the expression of CSF3R mRNA and protein. **Q** The colocalization between CSF3R-AS and RBMS3. **R**–**T** CSF3R probe enriched more RBMS3 after the overexpression of CSF3R-AS. **U** CSF3R-AS had no influence on the expression of RBMS3. (* represents *P* < 0.05, ** represents *P* < 0.01, *** represents *P* < 0.001, **** represents *P* < 0.0001).

Recently, several studies have shown that circRNAs can regulate the stability of parental mRNA. Interestingly, we found that there were 180 reverse complementary pairing bps between CSF3R-AS and CSF3R mRNA (Fig. 3A). Thus, we hypothesized that CSF3R-AS can bind with CSF3R mRNA and regulate its stability. To verify this hypothesis, we first investigated the correlation between the expression of CSF3R-AS and CSF3R mRNA in HCC. The results obtained from qRT-PCR suggested that there was a significant positive correlation between CSF3R-AS and CSF3R in HCC tissues (Fig. 3B). Then, we found that the expression of CSF3R mRNA and protein were significantly down regulated after the knock down of CSF3R-AS, and up regulated after the overexpression of CSF3R-AS (Fig. 3C–G). Finally, we performed the RNA stability experiment using actinomycin D. The results revealed that the knock down of CSF3R-AS reduced the stability of CSF3R mRNA, while the opposite was observed after the overexpression of CSF3R-AS (Fig. 3H, I).

Next, the RNA pull down experiment was performed to evaluate whether CSF3R-AS can bind with CSF3R mRNA. Figure 3J, K revealed that the CSF3R probe group can enrich more CSF3R-AS than the NC probe group. The results of FISH also suggest that there was a significant colocalization between CSF3R-AS and CSF3R mRNA in HCCLM3 cells and Huh6 cells, and they were both localized in the cytoplasm (Fig. 3L).

Many studies have shown that RNA binding proteins (RBPs) can directly bind with RNA and regulate RNA stability, transcription, and translating efficiency. Thus, we further analyzed potential RBPs that can bind with CSF3R-AS in the RBPMAP database. The results obtained suggested that 105 RBPs (Table S5) can bind with CSF3R-AS and 115 RBPs (Table S6) can bind with CSF3R mRNA (Fig. 3M). Among them, 12 RBPs can bind with CSF3R-AS but cannot bind with CSF3R, including A1C, DAZAP1, HNRNPD, KHDRBS1, PUM2, RBM3, RBM47, RBMS, RBMS2, RBMS3, SF1, and ZCRB1 (Fig. 3M). Thus, we further analyzed the subcellular localization of the 12 RBPs in the uniprot database. The results obtained revealed that only RBMS3 was mainly localized in the cytoplasm. Given that CSF3R-AS and CSF3R mRNA were both localized in the cytoplasm, we hypothesized that CSF3R-AS can function as a scaffold to bind with CSF3R mRNA and recruit RBMS3 to stabilize CSF3R. Next, we found that the expression of CSF3R mRNA and protein were upregulated after the overexpression of RBMS3, while the opposite were observed after the knock down of RBMS3 (Fig. 3N–P). Besides, Fig. 3Q suggests that CSF3R-AS and RBMS3 were colocalized in the cytoplasm. Meanwhile, we found that the expression of RBMS3 didn’t change after the overexpression of CSF3R-AS, but the CSF3R probe enriched more RBMS3 after the overexpression of CSF3R-AS, which means that CSF3R-AS has the potential to facilitate the binding of CSF3R mRNA and RBMS3 (Fig. 3R–U). All of the above results suggest that CSF3R-AS can bind with CSF3R mRNA and recruit RBMS3 to stabilize the expression of CSF3R.

### CSF3R-AS deletion mutation cannot regulate the expression of CSF3R mRNA and HCC progression

**Fig. 4:**
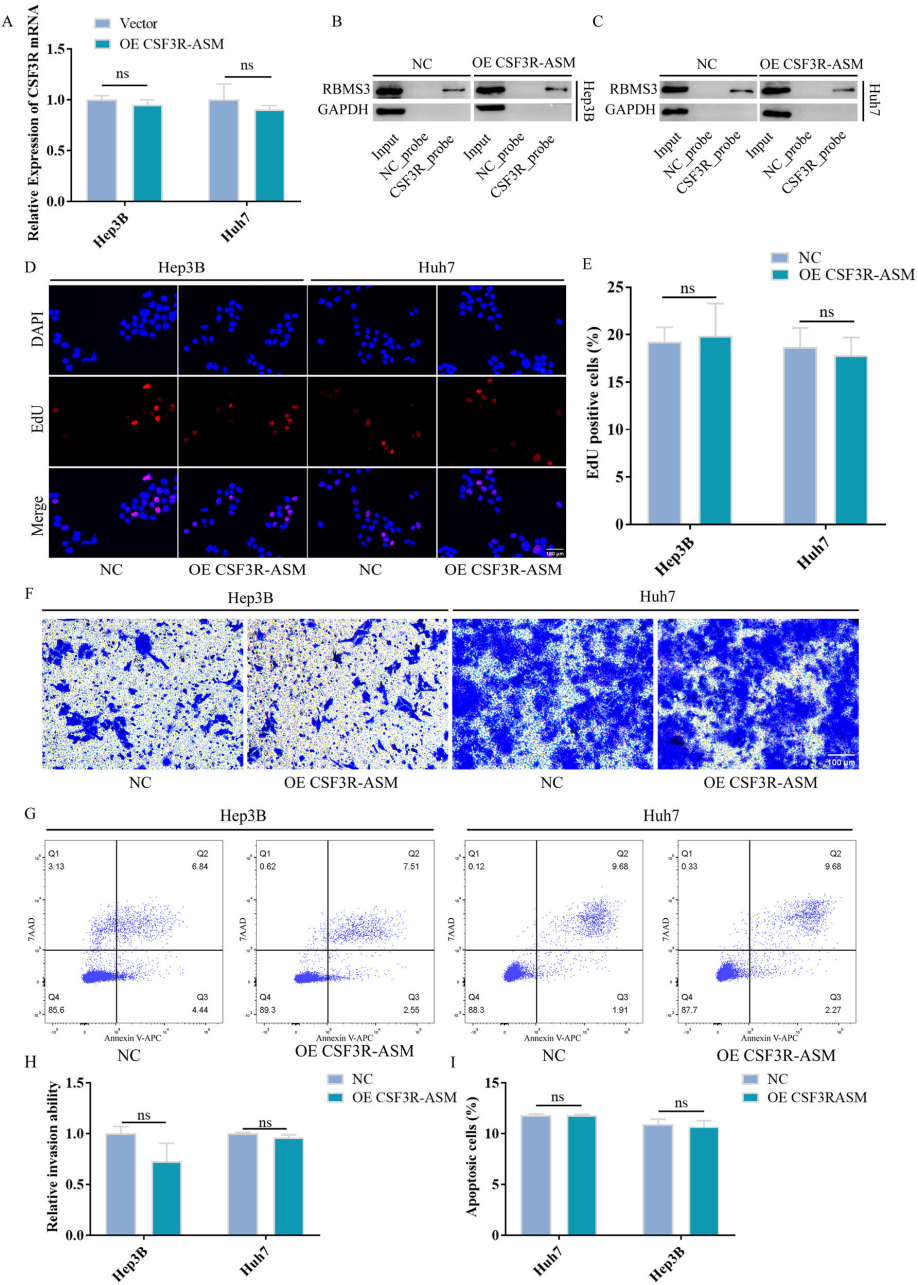
CSF3R-AS deletion mutation could not regulate the expression of CSF3R mRNA and HCC progression. **A** CSF3R-ASM cannot affect the expression of CSF3R mRNA. **B**, **C** CSF3R-ASM had no influence on the binding between CSF3R mRNA and RBMS3. **D**, **E** CSF3R-ASM had no influence on the proliferation of HCC cells. **F**–**I** CSF3R-ASM had no influence on the invasion and apoptosis of HCC cells. (ns: no significance).

Our previous results suggest that there were 180 reverse complementary pairing bps between CSF3R-AS and CSF3R mRNA, and CSF3R-AS can bind with CSF3R mRNA. Thus, we further explored the potential binding sequence between CSF3R-AS and CSF3R mRNA. We constructed the CSF3R-AS mutation plasmid, CSF3R-ASM, which lacks the 180 bps. Next, we detected the potential function of CSF3R-ASM. Figure 4A suggests that CSF3R-ASM cannot affect the expression of CSF3R mRNA. Similarly, CSF3R-ASM had no influence on the binding between CSF3R mRNA and RBMS3 (Fig. 4B, C). Furthermore, Fig. 4D–I suggests that CSF3R-ASM had no influence on the proliferation, invasion, and apoptosis of HCC cells. All of the above results suggest that CSF3R-AS deletion mutation cannot regulate the expression of CSF3R mRNA and HCC progression.

### CSF3R-AS promotes HCC progression via CSF3R-AS/CSF3R/JAK2/STAT3 positive feedback loop

**Fig. 5:**
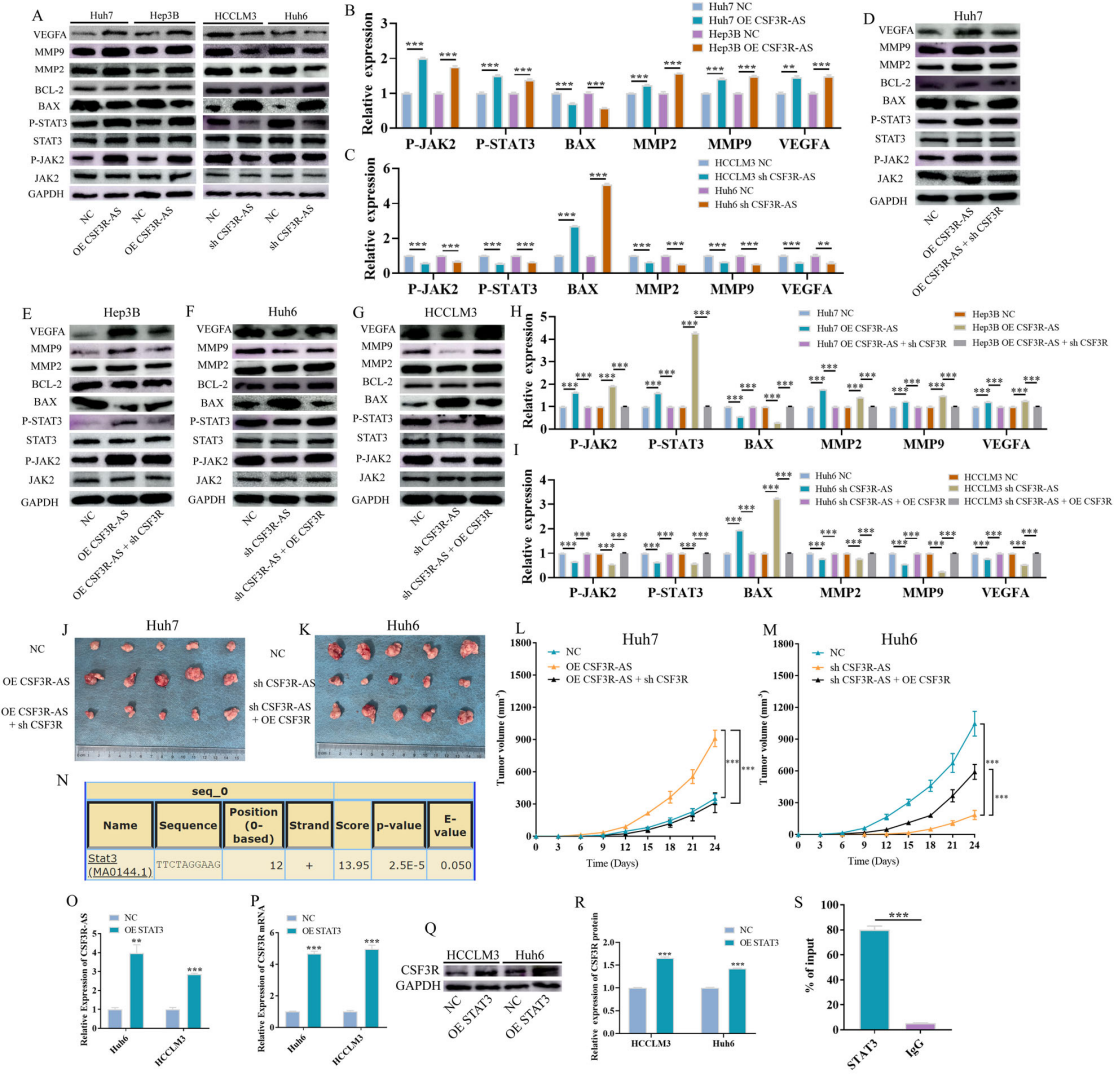
CSF3R-AS promoted HCC progression via the CSF3R-AS/CSF3R/JAK2/STAT3 positive feedback loop. **A** CSF3R-AS significantly activated the JAK2/STAT3 signaling pathway and downstream genes. **B**, **C** The quantitative statistics of (**A**). **D**–**G** The rescue experiments of CSF3R-AS activated the JAK2/STAT3 signaling pathway via CSF3R. **H**–**I** The quantitative statistics of (**D**–**G**). **J**–**M** CSF3R rescued the influence of CSF3R-AS on HCC proliferation in vivo (*n* = 5). **N** The binding sites between STAT3 and the CSF3R-AS promoter region. **O** Overexpression of STAT3 promoted the expression of CSF3R-AS. **P**–**R** Overexpression of STAT3 promoted the expression of CSF3R mRNA and protein. **S** The results of CHIP-qPCR. (** represents *P* < 0.01, *** represents *P* < 0.001).

CSF3R is a member of the type I cytokine receptor family and play important roles in the proliferation and differentiation of granulocytic hematopoietic cells via activating JAK2/STAT3 signaling pathway. However, the role of CSF3R in HCC has never been reported. Next, we treated the HCC cells with 40 ng/ml Granulocyte Colony-Stimulating Factor (G-CSF) and detected the down-stream signaling pathway. Figure S3A–H suggests that G-CSF can promote HCC proliferation and invasion but can also suppress apoptosis. Besides, the JAK2/STAT3 signaling pathway and down-stream target genes, including BAX, MMP2, MMP9, and VEGFA, were significantly activated after the treatment of G-CSF (Fig. S3I–J). Similarly, we found that the overexpression of CSF3R-AS significantly activated the JAK2/STAT3 signaling pathway, and the knock down of CSF3R-AS suppressed this pathway (Fig. 5A–C). Next, we knocked down CSF3R after the overexpression of CSF3R-AS, the results obtained suggested that CSFR rescued the influence of CSF3R-AS on the JAK2/STAT3 pathway (Fig. 5D–I). Similar results were observed when CSF3R was overexpressed after knocking down CSF3R-AS (Fig. 5D–I). All of the above results suggest that CSF3R-AS can activate the JAK2/STAT3 pathway via CSF3R.

Then, we further investigated whether CSF3R-AS promoted the progression of HCC via CSF3R. At first, we overexpressed CSF3R after the knock down of CSF3R-AS and analyzed the malignant phenotype of HCC cells. Figure S4A–K shows that CSF3R rescued the influence of CSF3R-AS on HCC proliferation, apoptosis, invasion, and angiogenesis. Similar results were observed when CSF3R was knocked down after the overexpression of CSF3R-AS (Fig. S5A–K). Besides, the in vivo experiments also suggested that CSF3R-AS can promote HCC progression via CSF3R (Fig. 5J–M).

Finally, to analyze why CSF3R-AS was up-regulated in HCC, we predicted the potential transcription factor of CSF3R-AS using the JASPAR and LASAGNA-Search2.0 databases. In the JASPAR database, we selected 949 transcription factors from Homo sapiens to perform the prediction. The results obtained suggest that there were 7429 potential binding sites between the CSF3R-AS promoter region and the 949 transcription factors. Interestingly, STAT3, a famous transcription factor, ranked 36^th^ (Top 0.5%) among the 7429 binding sites. The potential binding sequence is 12-22 bases: GTTCTAGGAAG. Meanwhile, we predicted the binding sites between STAT3 and the CSF3R-AS promoter region in the LASAGNA-Search2.0 database as well. The result was consistent with that of the JASPAR database, and the sequence was indeed GTTCTAGGAAG (Fig. 5N). Previous results have suggested that STAT3 can be activated and function as the down-stream gene of CSF3R-AS, and now we found that STAT3 may function as the transcription factor of CSF3R-AS. Thus, we speculated that there was a CSF3R-AS/CSF3R/JAK2/STAT3 positive feedback loop in HCC and it promoted HCC progression. Next, we investigated the expressions of CSF3R-AS and CSF3R after the overexpression of STAT3. The results obtained suggested that CSF3R-AS and CSF3R were significantly upregulated after the overexpression of STAT3 (Fig. 5O–R). Finally, we designed the PCR primers across both sides of the 12-22 bases of the promoter region. The results of CHIP-qPCR revealed that STAT3 can be significantly enriched compared to the IgG group (Fig. 5S). Thus, STAT3 can function as a transcription factor of CSF3R-AS and promote the expression of CSF3R-AS, which means that there is a CSF3R-AS/CSF3R/JAK2/STAT3 positive feedback loop in HCC.

### CSF3R-AS/CSF3R/JAK2/STAT3 positive feedback loop promotes the sorafenib resistance of HCC

**Fig. 6:**
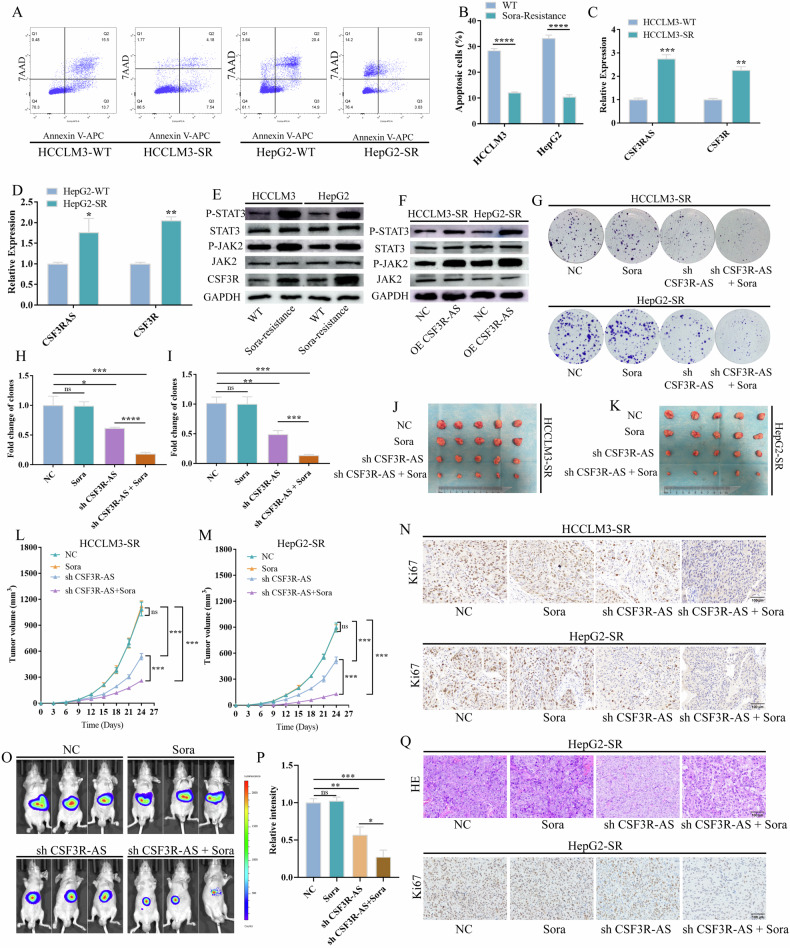
The CSF3R-AS/CSF3R/JAK2/STAT3 positive feedback loop promoted sorafenib resistance of HCC. **A**, **B** HCCLM3-SR and HepG2-SR were more resistant to sorafenib compared to the parental cells. **C**–**E** The CSF3R/JAK2/STAT3 signaling pathway was activated in the sorafenib-resistant cells. **F** Overexpression of CSF3R-AS activated JAK2/STAT3 signaling pathway in sorafenib-resistant cells. **G**–**I** The combination of sorafenib and CSF3R-AS knock down significantly suppressed the proliferation of sorafenib-resistant cells. **J**–**M** The combination of sorafenib and CSF3R-AS knock down significantly suppressed the proliferation of sorafenib-resistant cells in vivo (*n* = 5). **N** The combination of sorafenib and CSF3R-AS knock down significantly suppressed the expression of Ki67. **O**, **P** The combination of sorafenib and CSF3R-AS knock down significantly suppressed the proliferation of sorafenib-resistant cells in orthotopic tumor model. **Q** The combination of sorafenib and CSF3R-AS knock down significantly suppressed the expression of Ki67 in orthotopic tumor model. (Sora: sorafenib, * represents *P* < 0.05, ** represents *P* < 0.01, *** represents *P* < 0.001, **** represents *P* < 0.0001).

Several studies have reported that the JAK2/STAT3 signaling pathway plays important roles in the sorafenib resistance of HCC. Thus, we speculated that CSF3R-AS may function as an important regulator to promote HCC sorafenib resistance via the CSF3R/JAK2/STAT3 positive feedback loop. Next, to validate our hypothesis, we tried to establish sorafenib resistant HCC cell lines using HCCLM3, HepG2, MHCC97H, Hep3B and Huh7. Only HCCLM3 and HepG2 cells were successfully established, and the resistant cells were named as HCCLM3-SR and HepG2-SR. At first, we detected the sorafenib-resistant abilities of HCCLM3-SR and HepG2-SR. Figure 6A, B suggests that, compared with the parental wild-type cells, the apoptosis ratio was significantly reduced after the treatment with sorafenib. Next, we investigated the activation status of the CSF3R-AS/CSF3R/JAK2/STAT3 positive feedback loop in HCCLM3-SR and HepG2-SR. Figure 6C–E suggests that CSF3R-AS and CSF3R were significantly upregulated in the sorafenib-resistant cells. Meanwhile, JAK2/STAT3 was activated as well. Besides, CS3R-AS can still activate JAK2/STAT3 signaling pathway in HCC sorafenib-resistant cells (Fig. 6F). Next, we tried to block the CSF3R-AS/CSF3R/JAK2/STAT3 positive feedback loop to relieve sorafenib resistance. Figure 6G–I suggests that sorafenib alone had no influence on the proliferation of sorafenib-resistant cells, and the knock down of CSF3R-AS suppressed proliferation, while the combination of sorafenib and CSF3R-AS knock down had the most significant inhibitory effect. Also, similarly results were obtained in the subcutaneous tumor model (Fig. 6J–N). Finally, we performed the HCC orthotopic tumor model using HepG2-SR cells to further prove the results of subcutaneous tumor model. Figure 6O, P suggest that the combination of sorafenib and CSF3R-AS knock down significantly inhibited the progression of HepG2-SR. The expression of Ki67 was also significantly down regulated using the combination strategy (Fig. 6Q). All of the above results suggest that the CSF3R-AS/CSF3R/JAK2/STAT3 positive feedback loop was activated in the sorafenib-resistant cells, and blocking the loop may relieve the sorafenib resistance of HCC.

## Discussion

In recent years, a large number of studies have shown that circRNAs can also regulate the development of malignant tumors through the formation of positive feedback loops. For example, Zhao et al. showed that circUHRF1 was upregulated in oral squamous cell carcinoma and correlated with a poor prognosis. Functionally, circUHRF1 could promote the proliferation, migration, invasion, and EMT of oral squamous cell carcinoma; mechanistically, circUHRF1 can bind with miR-526b-5p and promote the expression of c-Myc, which acts as a transcription factor to initiate the transcription of TGF-β and ESRP1, and ESRP1 could act as a splicing factor and promote the cyclization of circUHRF1, and then circUHRF1/miR-526b-5p/c-Myc/TGF-β1/ESRP1 positive feedback loop could promote the progression of oral squamous cell carcinoma [39].

CSF3R-AS is an antisense circRNA and has a 180-base reverse complementary pairing sequence with its parental mRNA CSF3R, and thus can bind directly to its parental mRNA. Meanwhile, CSF3R-AS can act as a scaffold molecule to recruit RBMS3. RBMS3, known as RNA binding motif single-stranded interacting protein 3, is an RNA-binding protein that contains two RNA-binding domains and binds to A- and U-rich RNA sequences. RBMS3 is mainly located in the cytoplasm, and plays a key role in stabilizing mRNA. C James Block found that RBMS3 bound to and stabilized the mRNA of PRRX1, which in turn promoted the development of triple-negative breast cancers. Therefore, CSF3R-AS can bind to its parental mRNA and recruit RBMS3, which in turn improves the stability of its parental mRNA CSF3R.

CSF3R is a member of the class I cytokine receptor superfamily that is widely involved in the regulation of granulocyte hematopoietic cell proliferation and differentiation. In recent years, several studies have shown that CSF3R may also play a key role in the progression of solid tumors. In this study, we found that CSF3R-AS could activate the JAK2/STAT3 signaling pathway and regulate the expression of downstream target genes (MMP2, MMP9, BCL2, BAX, and VEGFA) by increasing the expression of CSF3R, thereby promoting the proliferation, invasion, and angiogenesis of HCC. At the same time, we found that phosphorylated STAT3 could act as a transcription factor for CSF3R-AS, initiate transcription, and then in turn regulate the expression of CSF3R. That is, there is a CSF3R-AS/CSF3R/JAK2/STAT3 positive feedback loop in HCC, which promotes HCC progression.

Nowadays, the development of therapy resistance is becoming a nonnegligible clinical problem and challenge, especially for the target therapy. Sorafenib, as an important treatment for advanced HCC patients, can significantly suppressed the progression of HCC by blocking multiple kinases. However, patients treated with sorafenib tend to become drug resistant within six months, and the emergence of drug resistance greatly limits the clinical efficacy of Sorafenib [34]. Recent studies have indicated that the activation of STAT3 was observed in sorafenib-resistant HCC, but the reason for the aberrant activation is still unclear. On the basis of our previous study, we further assumed that CSF3R-AS may act as a molecular switch to activate the JAK2/STAT3 pathway in sorafenib-resistant cells. To prove the assumption, we established sorafenib-resistant HCC cell lines and found that the CSF3R-AS/CSF3R/JAK2/STAT3 positive feedback loop was indeed activated in the resistant cells, and blocking this loop increased the sensitivity of HCC to sorafenib, which is expected to provide a new therapeutic target for HCC.

However, there are still some limitations. Firstly, the the sample size of HCC patients was relatively small, and expanding the sample size may make the results more accurate. Secondly, in our experiments, we just detected the expression of CS3R-AS in HCC and adjacent non-tumor tissues rather than in serum samples, to a certain extent, it will limit the clinical application.

**Fig. 7:**
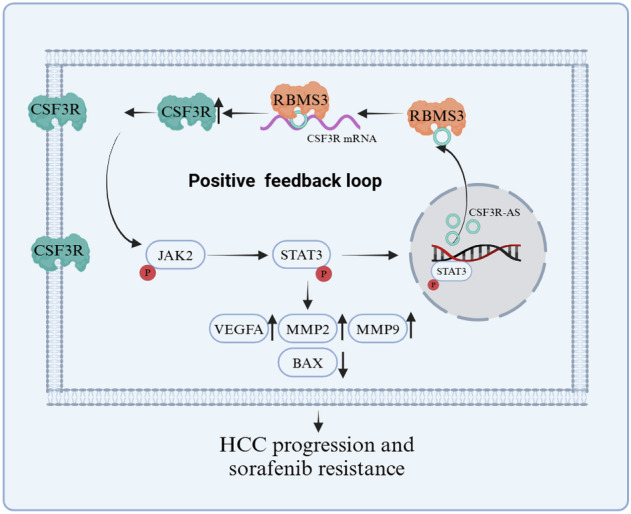
Schematic representation of the model. CSF3R-AS promotes hepatocellular carcinoma progression and sorafenib resistance through the CSF3R/JAK2/STAT3 positive feedback loop.

In summary, we identified the existence of the antisense circRNA CSF3R-AS, and found that: (1) CSF3R-AS was up-regulated in HCC and correlated with a poor prognosis. (2) CSF3R-AS bound to its parental mRNA CSF3R and recruited RBMS3 to stabilize its parental mRNA, thereby activating the downstream JAK2/STAT3 signaling pathway. (3) STAT3 can act as a transcription factor for CSF3R-AS and promote CSF3R-AS expression, which means that there was a CSF3R-AS/CSF3R/JAK2/STAT3 positive feedback loop in HCC. (4) A CSF3R-AS/CSF3R/JAK2/STAT3 positive feedback loop existed in sorafenib-resistant HCC cells, and blocking this loop was expected to enhance the sensitivity of HCC to sorafenib (Fig. 7).

## Materials and methods

### Patients specimens’ collection and cell culture

All the HCC tumors and paired adjacent noncancerous tissues were collected from 52 patients who were diagnosed with HCC and had undergone surgery at Xiangya Hospital, Central South University, Changsha, Hunan, China. Histologic and pathologic diagnoses of tissue samples were independently confirmed by 2 experienced histopathologists. Patients were excluded if the follow-up data and laboratory examination information were incomplete. All the tissues were preserved by freezing them with liquid nitrogen and subsequently stored at a temperature of –80 °C. All the patients had signed the informed consent. The study was approved by the Xiangya Hospital Ethics Committee, and the procedures were conducted according to the Declaration of Helsinki. Detailed information on the operated patients is provided in Supplemental Tables S1-3.

All the cell lines, including Huh6, HCCLM3, PLC/PRF/5, Huh7, Hep3B and LO2 cells were purchased from the Institutes of Biomedical Sciences. Huh6, HCCLM3, Huh7, and Hep3B were cultured with DMEM medium, PLC was cultured with MEM medium, and LO2 was cultured with 1640 medium. The medium was added with 10% Fetal Bovine Serum (FBS, Umedium He Fei China) and 1% penicillin/streptomycin. The cells were cultured in a humidified incubator at 37 °C, in an atmosphere of 5% CO_2_.

### Next generation sequencing

The total RNA of three HCC tumor tissues and paired adjacent noncancerous tissues were extracted using Trizol (Accurate Biology, China). The sequencing were performed by Aksomics (Shanghai, China). The differential expressed genes was analyzed with EdgeR version 3.08. Benjamini- Hochburg method was used to calculate the adjusted *P* values. The threshold was adjusted *P* < 0.05.

### Quantitative RT-PCR (qRT-PCR)

The total RNA in tissues and cells were extracted using TRIZOL (Accurate Biology, Changsha, China). After measuring the concentration of RNA, 1 μg total RNA was used to perform the reverse transcription using the RT Kit with gDNA Clean for qPCR (Accurate Biology, Changsha, China). Then the product of reverse transcription was diluted 10 times with deionized water. Finally, qRT-PCR was performed using the Premix Pro Taq HS qPCR Kit (Accurate Biology, Changsha, China) on ViiA™ 7 RT-PCR system. All the primers are summarized in Table S4.

### Western blot (WB)

The proteins in tissues and cells were extracted using RIPA (Beyotime, Shanghai, China) with 10% Protease and Phosphatase Inhibitor (NCM biotech, Suzhou, China). After measuring the concentration of protein, 30 μg proteins were electrophoresed under 80 V for 30 min and then 120 V for 60 min. Then, the gel was transferred to the PVDF membranes under 250 mA for 100 min. Subsequently, the membranes were blocked with 5% non-fat milk or BSA at room temperature for 60 min. Then the membranes were incubated with a primary antibody at 4 °C overnight. The next day, the membranes were washed three times with TBST and incubated with a secondary antibody at room temperature for 60 min. Finally, the blots were visualized using the ChemiDocXRS+ System (Bio-Rad, Hercules, CA). The antibodies used are GAPDH (Proteintech, 10494-1-AP), CSF3R (Absin, abs139063), RBMS3 (AiFang biological, AF10517), JAK2 (CST, 3230 T), STAT3 (CST, 9139 T), BAX (AiFang biological, AF300120), BCL2 (AiFang biological, AF301143), MMP2 (AiFang biological, AF300234), VEGFA (AiFang biological, AF06392) and MMP9 (AiFang biological, AF300248).

### Immunohistochemistry (IHC)

At first, the sections were dewaxed and dehydrated using xylene and ethanol. Then the sections were boiled with citrate for 10 min. Next, the sections were incubated with 3% H_2_O_2_ at room temperature for 10 min. After blocking with goat serum, the sections were incubated with primary antibodies overnight at 4 °C. The next day, the sections were washed three times with PBST at room temperature and incubated with a secondary antibody at room temperature for 30 min. Finally, the sections were stained with DAB and hematoxylin, and an N2-Mi8 microscope was used to capture the images. The antibody used is Ki67 (Proteintech, 27309-1-AP).

### Colony-formation assays

The cells were seeded into a 6-well plate at a concentration of 500 cells/well. The medium was replaced every three days. About two weeks later, the cells were fixed using 4% paraformaldehyde at room temperature for 30 min. Then, the cells were stained with 0.1% crystal violet for 30 min.

### EdU assay

The cells were seeded into a 96-well plate at a concentration of 10,000 cells/well. The next day, the cells were incubated with the EdU solution (1:1000) for 2 h. Then, the cells were fixed with 4% paraformaldehyde for 20 min. Next, the cells were incubated with the Apollo dye solution (C10310-1, RiboBIO, China) for 30 min. Then, the cells were washed with 0.5% TritonX-100 for 10 min. Finally, the cells were incubated with the DAPI solution at room temperature for 10 min. The N2-Mi8 microscope was used to capture the images.

### Transwell assay

At first, the upper chamber was added with the Matrigel. Then, 50,000 cells were seeded into the upper chamber with 200 μl DMEM (1% serum). Then, 600 μl DMEM (20% serum) were added into the lower chamber. About 24 h later, the cells of the upper chamber were removed, and the cells of the lower chamber were fixed with 4% paraformaldehyde for 20 min. Then, the cells were stained with 0.1% crystal violet for 30 min. The N2-Mi8 microscope was used to capture the images.

### Apoptosis test

The cells were seeded into a 6-well plate, and the concentration was 2 × 10^5^ cells/well. The next day, the cells were re-suspended in 200 μl of the binding buffer. Then, 1 μl of Annexin V-APC and 2 μl of 7-AAD were added to the binding buffer. The cells were then incubated at room temperature for 5 min. Finally, a flow cytometer was used to detect the cells’ apoptosis.

### Rnase R treatment

The total RNA (5 μg) was treated with 1 μl Rnase R at 37 °C for 15 min. The RNA was then incubated at 70°C for 30 min. Subsequently, the product was used to perform reverse transcription and qRT-PCR.

### Fluorescence in situ hybridization (FISH)

The cells were seeded on the cover glass of a 24-well plate; the concentration was 50,000 cells/well. Then, the cells were fixed with 4% paraformaldehyde at room temperature for 30 min. The cells were then treated with 0.1% TritonX-100 for 15 min. Next, the cells were incubated with 2×SSC buffer at 37 °C for 30 min. After denaturation of the probe, the cells were incubated with the probe at 37°C overnight. The next day, the cells were washed with 4×SSC buffer and 0.1% Tween 20 for 5 min. After washing with 2×SSC and 1×SSC for 5 min, the cells were incubated with DAPI for 5 min. Finally, the N2-Mi8 microscope was used to capture the images.

The probe was designed, validated and purchased from the RiboBio (Guangzhou, China) company. The sequence are as follows: CSF3R-AS: 5ʹ-ACCTGAGACCCACCTCAACCACACTC-3ʹ; CSF3R: 5ʹ-CAGCTTCACTCTGAAGAGTTTC-3ʹ.

### Construction of plasmid, lentivirus and transfection

The plasmid and lentivirus were constructed by GeneChem, Shanghai, China. After infection with the lentivirus, the medium was replaced. Three days later, the cells were treated with 4 μg/ml puromycin for 2 weeks. Then the efficacy of overexpression and knockdown was detected by qRT-PCR and WB.

### RNA stability experiment

The cells were treated with 5 μg/ml Dactinomycin D. The RNA was extracted after 0, 2, 4, 8, and 12 h. After reverse transcription, the expression of gene was detected using qRT-PCR.

### RNA pull-down

The cells were seeded into a 10 cm dish. About 1 × 10^8^ cells were collected to perform the experiment. The experiment was conducted using the RNA Antisense Purification (RAP) Kit (Bersinbio, Guangzhou, China) according to the manufacturer’s instructions. Finally, the expression of RNA and protein was detected using qRT-PCR and WB.

### Chromatin immunoprecipitation-qPCR

The cells were seeded into a 10 cm dish. About 1 × 10^8^ cells were collected to perform the experiment. The experiment was conducted using the ChIP Assay Kit (Beyotime, Shanghai, China) according to the manufacturer’s instructions. Finally, the enrichment of DNA was detected using qRT-PCR.

### In vivo xenograft studies

For the subcutaneous tumor model, the male BALB/C nude mice (4 weeks old, weight 16 g) were divided into 4 groups. 5 × 10^6^ cells infected with lentivirus were injected subcutaneously into the armpit. The tumor size was measured every three days. Three weeks later, the mice were sacrificed. The expression of genes was detected using qRT-PCR, WB and IHC.

For the lung metastasis model, the male BALB/C nude mice (4 weeks old, weight 16 g) were divided into 4 groups. 5 × 10^6^ cells infected with lentivirus were injected into the tail vein. Three weeks later, in vivo imaging and HE staining were used to detect the metastasis status.

### Statistical analysis

The statistical analysis was performed using GraphPad Prism. Student’s t-test was used to compare the difference between two groups, and a one-way analysis of variance was used to compare the difference between multiple groups. The data obtained are shown as average values ± SD. Kaplan-Meier survival curves were used to analyze the correlation between CSF3R-AS and HCC OS. Chi-square test and Cox regression analysis were used to analyze the correlation between CSF3R-AS and HCC Clinicopathological characteristics. *P* < 0.05 was considered statistically significant.

## Supplementary information

Figure S1

Figure S2

Figure S3

Figure S4

Figure S5

Table S1

Table S2

Table S3

Table S4

Table S5

Table S6

Supplementary figure and table legends

Uncropped original western blots

## Data Availability

All data are available upon request.
