# Supplementary figures and images for: CSF3R-AS promotes hepatocellular carcinoma progression and sorafenib resistance through the CSF3R/JAK2/STAT3 positive feedback loop
Source: Cell Death Dis. 2025 Mar 28;16(1):217. doi: 10.1038/s41419-025-07558-4 (PMC11953311; doi:10.1038/s41419-025-07558-4)

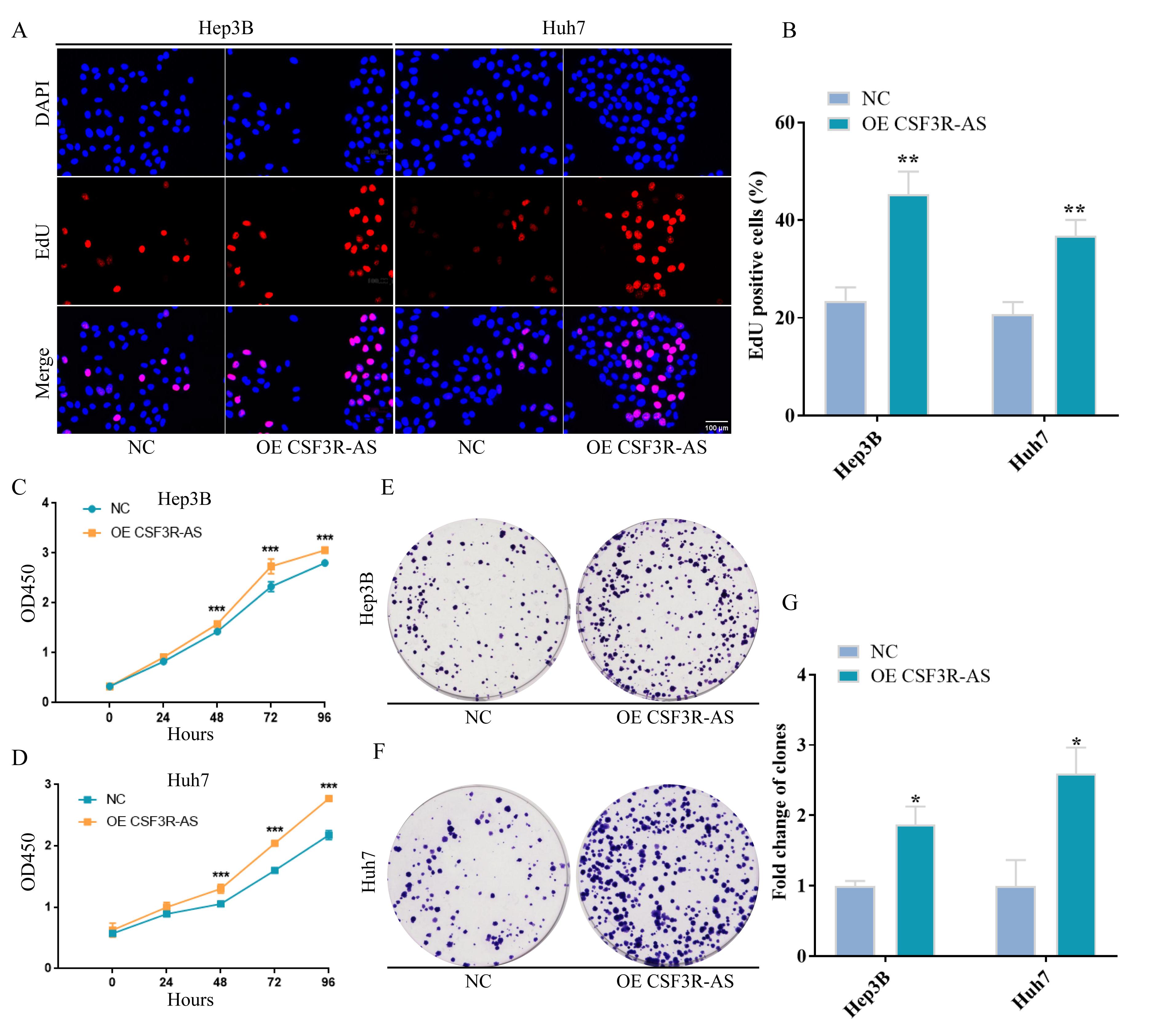

Supplement: Supplementary file 1 — Figure S1 [file 41419_2025_7558_MOESM1_ESM.jpg]

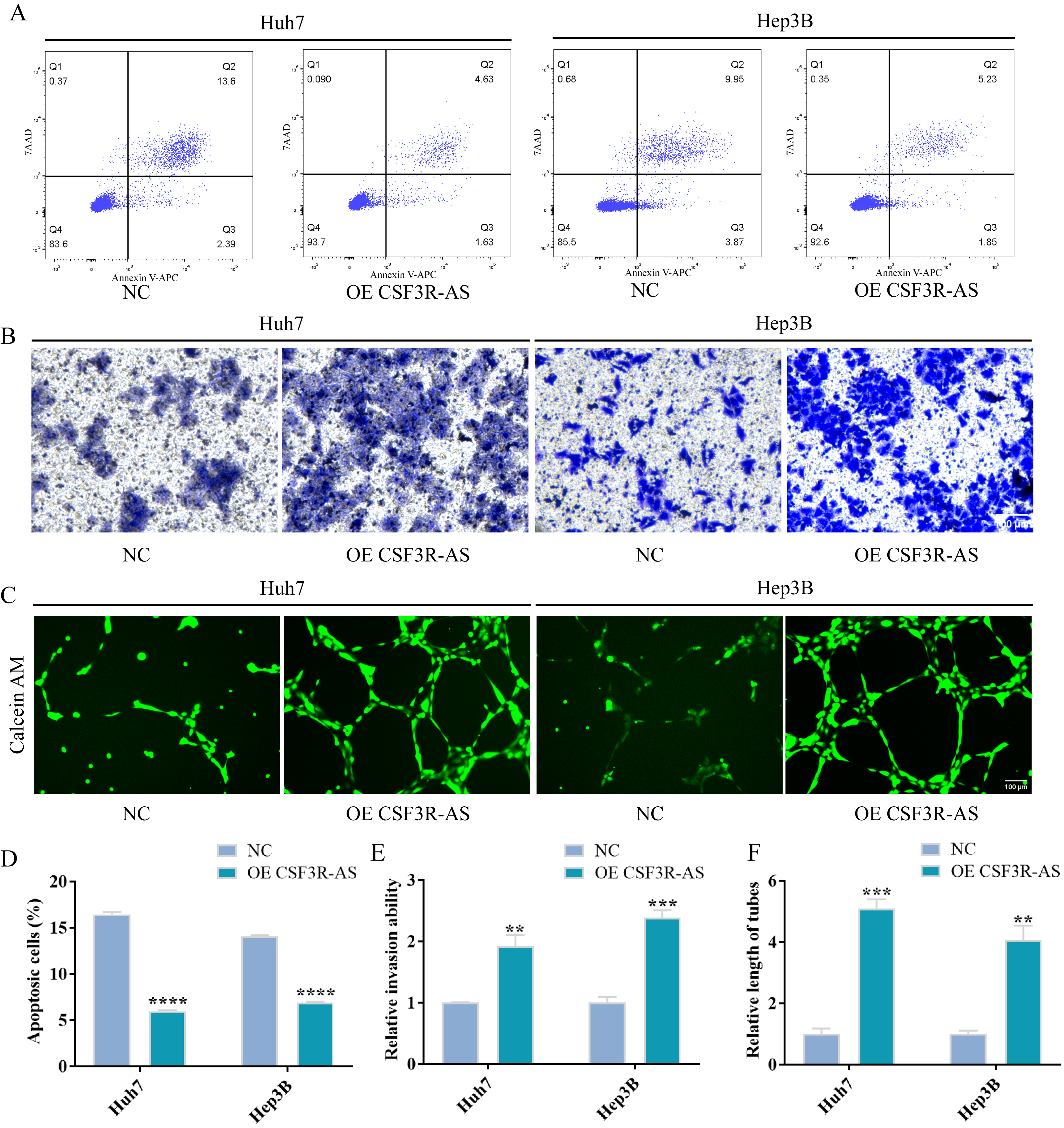

Supplement: Supplementary file 2 — Figure S2 [file 41419_2025_7558_MOESM2_ESM.jpg]

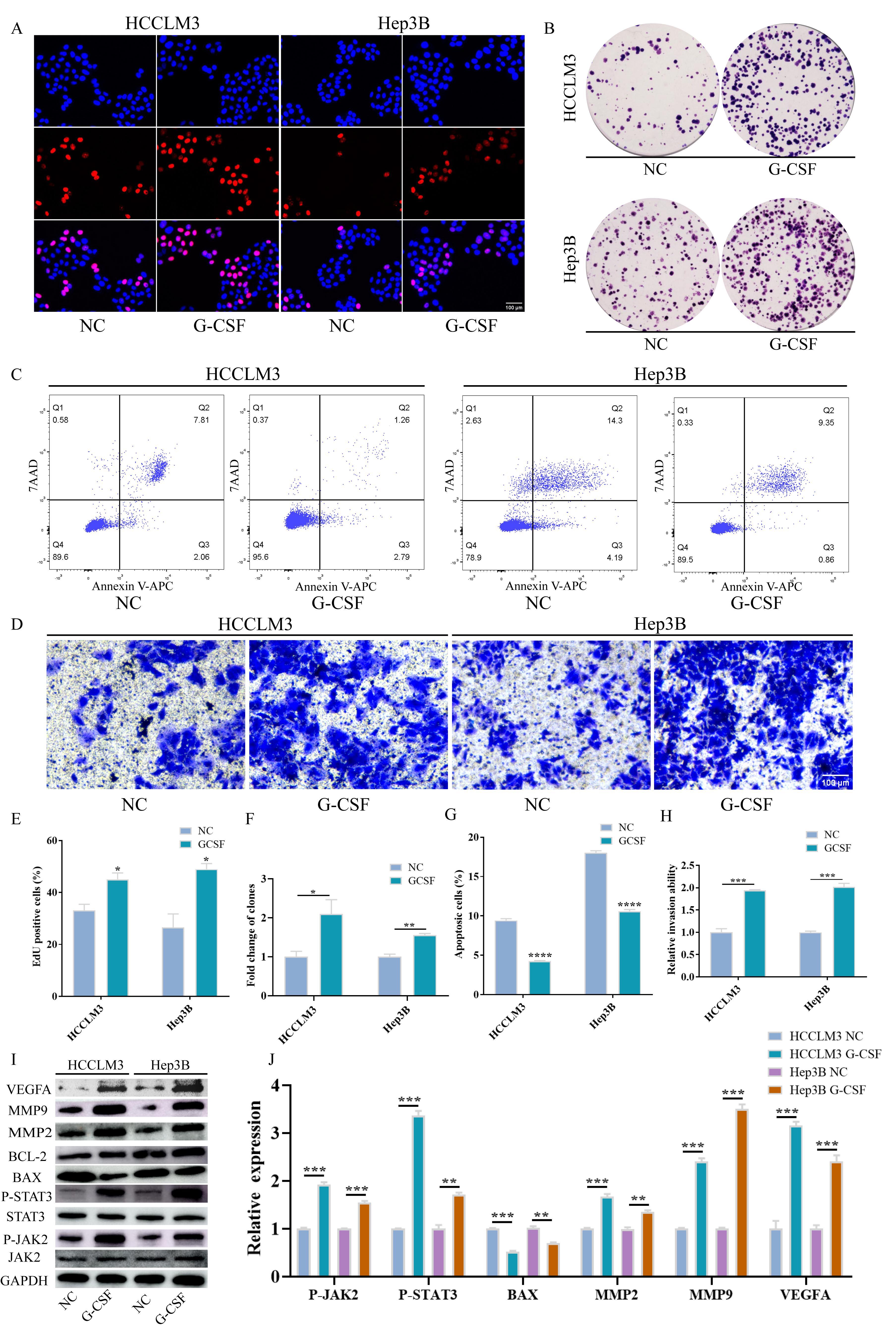

Supplement: Supplementary file 3 — Figure S3 [file 41419_2025_7558_MOESM3_ESM.jpg]

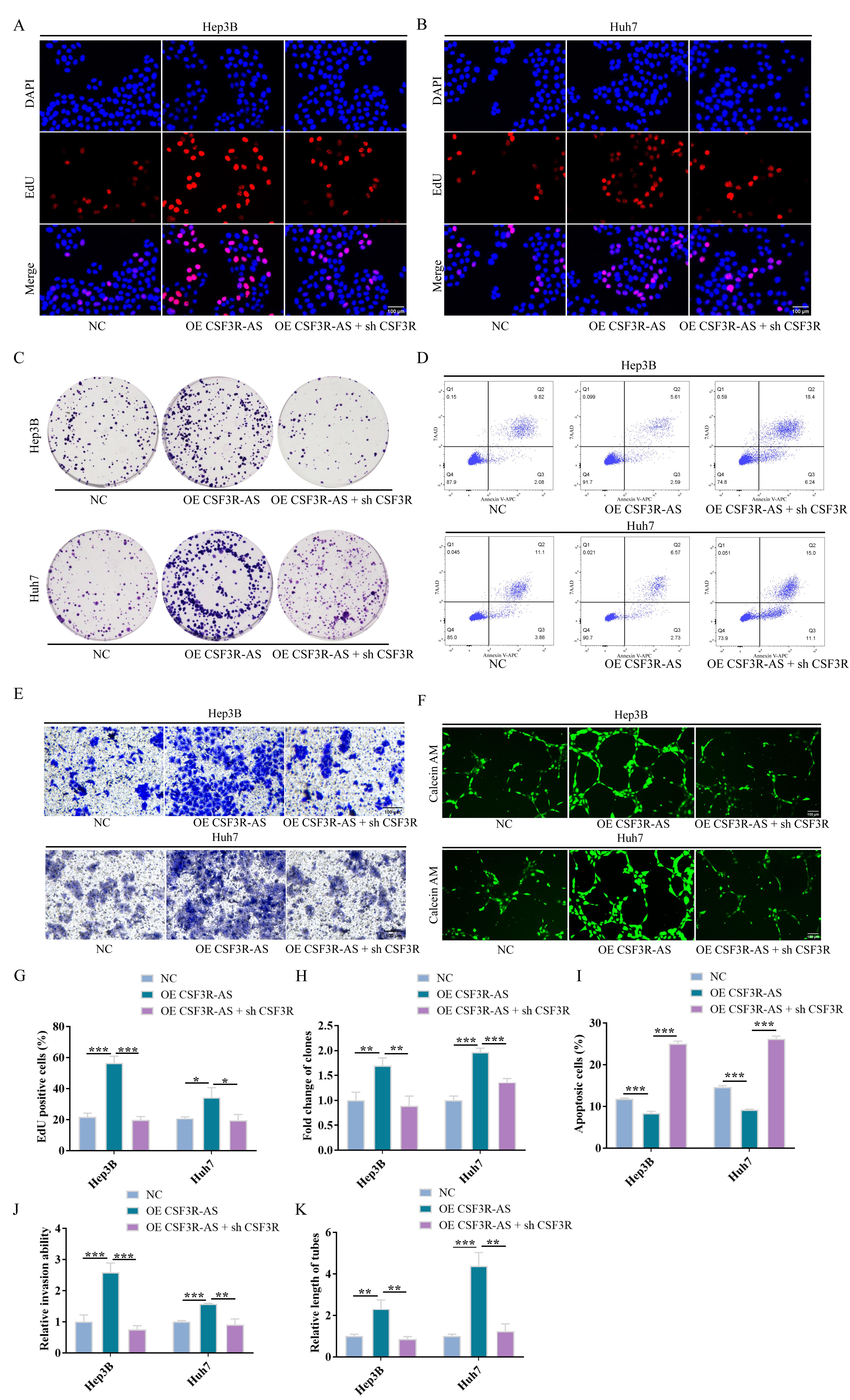

Supplement: Supplementary file 4 — Figure S4 [file 41419_2025_7558_MOESM4_ESM.jpg]

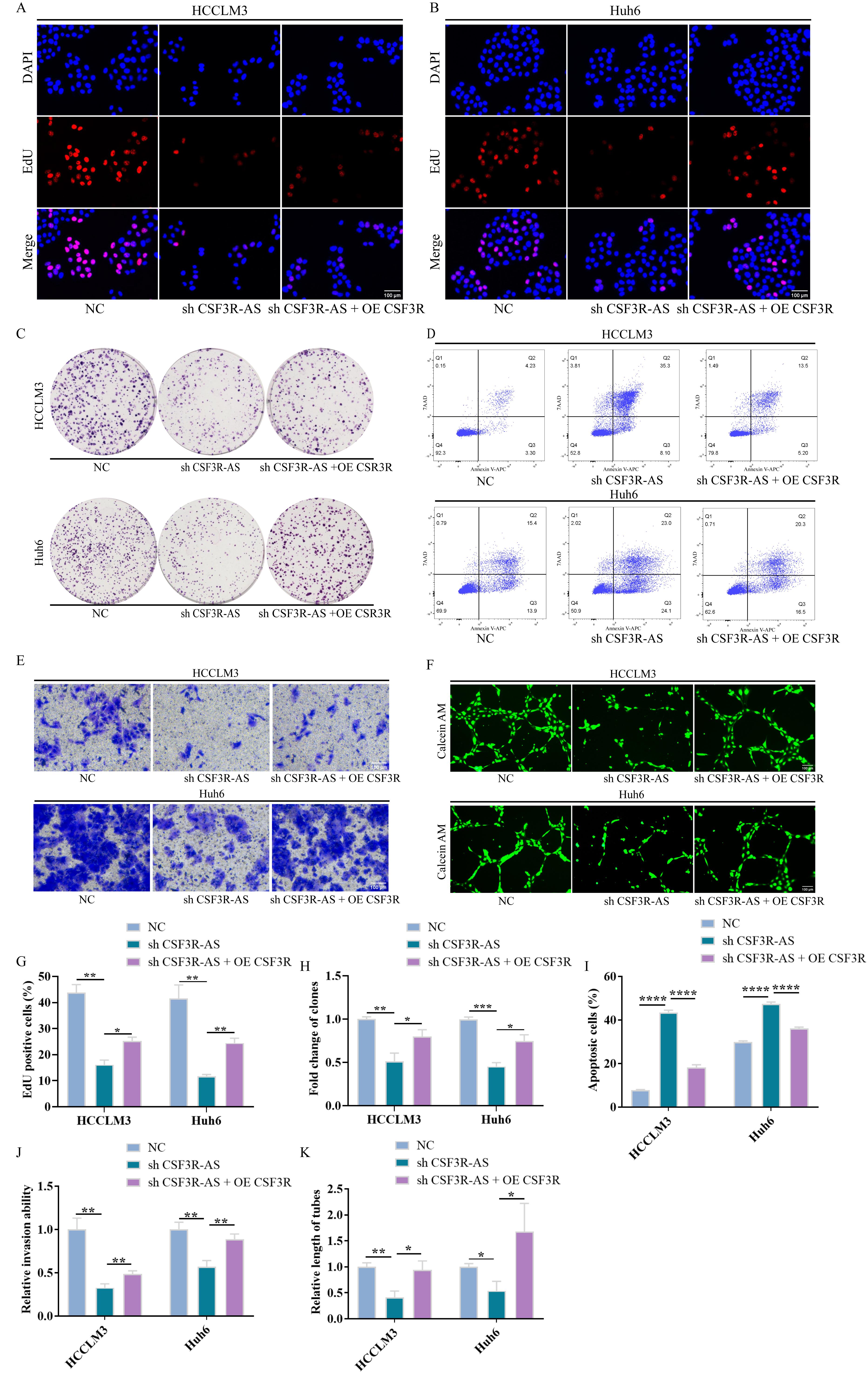

Supplement: Supplementary file 5 — Figure S5 [file 41419_2025_7558_MOESM5_ESM.jpg]
