# Supplementary material for: CSF3R-AS promotes hepatocellular carcinoma progression and sorafenib resistance through the CSF3R/JAK2/STAT3 positive feedback loop
Source: Cell Death Dis. 2025 Mar 28;16(1):217. doi: 10.1038/s41419-025-07558-4 (PMC11953311; doi:10.1038/s41419-025-07558-4)
Supplement: Supplementary file 10 — Table S5 [file 41419_2025_7558_MOESM10_ESM.docx]

Predictions for sequence: CSF3R-AS

Calculation parameters:

Genome: Human (hg38)

Selected motifs: All Human/Mouse motifs

Stringency level: High

Conservation filter: On

======================================================================================================================

Protein: A1CF(Hs/Mm)

Sequence Position Genomic Coordinate Motif K-mer Z-score P-value

397 chr1:36474192 wuaauur uucauua 2.022 2.16e-02

421 chr1:36474216 wuaauur uuauuua 2.600 4.66e-03

425 chr1:36474220 wuaauur uuaacug 2.144 1.60e-02

Protein: BOLL(Hs/Mm)

Sequence Position Genomic Coordinate Motif K-mer Z-score P-value

368 chr1:36474163 guguua guguuu 3.000 1.35e-03

597 chr1:36474392 uuuguuu uuuuuuu 3.737 9.31e-05

598 chr1:36474393 uuuguuu uuuuuuu 3.737 9.31e-05

599 chr1:36474394 uuuguuu uuuuuuu 3.737 9.31e-05

600 chr1:36474395 uuuguuu uuuuuuu 3.737 9.31e-05

601 chr1:36474396 uuuguuu uuuuuuu 3.737 9.31e-05

602 chr1:36474397 uuuguuu uuuuuuu 3.737 9.31e-05

603 chr1:36474398 uuuguuu uuuuuuu 3.737 9.31e-05

604 chr1:36474399 uuuguuu uuuuuuu 3.737 9.31e-05

605 chr1:36474400 uuuguuu uuuuuuu 3.737 9.31e-05

606 chr1:36474401 uuuguuu uuuuuuu 3.737 9.31e-05

607 chr1:36474402 uuuguuu uuuuuuu 3.737 9.31e-05

608 chr1:36474403 uuuguuu uuuuuuu 3.737 9.31e-05

609 chr1:36474404 uuuguuu uuuuuuu 3.737 9.31e-05

610 chr1:36474405 uuuguuu uuuuuuu 3.737 9.31e-05

611 chr1:36474406 uuuguuu uuuuuuu 3.737 9.31e-05

612 chr1:36474407 uuuguuu uuuuuuu 3.737 9.31e-05

613 chr1:36474408 uuuguuu uuuuuuu 3.737 9.31e-05

614 chr1:36474409 uuuguuu uuuuuuu 3.737 9.31e-05

615 chr1:36474410 uuuguuu uuuuuug 3.211 6.61e-04

851 chr1:36474646 uuuguuu uuuguuu 3.355 3.97e-04

852 chr1:36474647 guguua uuguuu 2.427 7.61e-03

853 chr1:36474648 uuuguuu uguuuuu 3.092 9.94e-04

854 chr1:36474649 uuuguuu guuuuuu 3.171 7.60e-04

855 chr1:36474650 uuuguuu uuuuuuc 2.961 1.53e-03

1142 chr1:36474937 guguua cuguua 2.629 4.28e-03

1149 chr1:36474944 guguua cugaua 2.820 2.40e-03

1542 chr1:36475337 guguua guguug 2.843 2.23e-03

Protein: BRUNOL4(Hs/Mm)

Sequence Position Genomic Coordinate Motif K-mer Z-score P-value

46 chr1:36473841 kgugukk ugugagg 1.689 4.56e-02

98 chr1:36473893 kgugukk ugugggu 3.135 8.59e-04

100 chr1:36473895 kgugukk ugggugg 2.905 1.84e-03

111 chr1:36473906 kgugukk aguguga 3.068 1.08e-03

113 chr1:36473908 kgugukk ugugagg 2.986 1.41e-03

367 chr1:36474162 kgugukk aguguuu 2.581 4.93e-03

369 chr1:36474164 kgugukk uguuuga 2.527 5.75e-03

636 chr1:36474431 kgugukk ucugugg 2.405 8.09e-03

851 chr1:36474646 kgugukk uuuguuu 2.162 1.53e-02

853 chr1:36474648 kgugukk uguuuuu 2.176 1.48e-02

1656 chr1:36475451 kgugukk ugagugu 2.230 1.29e-02

1658 chr1:36475453 kgugukk agugugg 2.257 1.20e-02

1660 chr1:36475455 kgugukk ugugguu 2.095 1.81e-02

Protein: BRUNOL5(Hs/Mm)

Sequence Position Genomic Coordinate Motif K-mer Z-score P-value

98 chr1:36473893 ugugukk ugugggu 3.337 4.23e-04

100 chr1:36473895 ugugukk ugggugg 3.062 1.10e-03

111 chr1:36473906 ugugukk aguguga 3.163 7.81e-04

113 chr1:36473908 ugugukk ugugagg 3.137 8.53e-04

367 chr1:36474162 ugugukk aguguuu 2.812 2.46e-03

369 chr1:36474164 ugugukk uguuuga 2.812 2.46e-03

373 chr1:36474168 ugugukk ugaguuu 2.625 4.33e-03

849 chr1:36474644 ugugukk ucuuugu 1.988 2.34e-02

851 chr1:36474646 ugugukk uuuguuu 2.100 1.79e-02

853 chr1:36474648 ugugukk uguuuuu 2.188 1.43e-02

1656 chr1:36475451 ugugukk ugagugu 2.387 8.49e-03

1658 chr1:36475453 ugugukk agugugg 2.337 9.72e-03

1660 chr1:36475455 ugugukk ugugguu 2.213 1.34e-02

Protein: BRUNOL6(Hs/Mm)

Sequence Position Genomic Coordinate Motif K-mer Z-score P-value

98 chr1:36473893 ugugdkg ugugggu 2.693 3.54e-03

100 chr1:36473895 ugugdkg ugggugg 2.933 1.68e-03

113 chr1:36473908 ugugdkg ugugagg 3.387 3.53e-04

636 chr1:36474431 ugugdkg ucugugg 1.827 3.38e-02

1658 chr1:36475453 ugugdkg agugugg 3.147 8.25e-04

1660 chr1:36475455 ugugdkg ugugguu 3.253 5.71e-04

Protein: CELF1(Hs/Mm)

Sequence Position Genomic Coordinate Motif K-mer Z-score P-value

367 chr1:36474162 uauguuu aguguuu 2.129 1.66e-02

636 chr1:36474431 uauguuu ucugugg 2.186 1.44e-02

Protein: CNOT4(Hs/Mm)

Sequence Position Genomic Coordinate Motif K-mer Z-score P-value

623 chr1:36474418 gacaga gacaga 2.196 1.40e-02

624 chr1:36474419 acacag acagag 1.692 4.53e-02

1014 chr1:36474809 acacag acacug 2.397 8.26e-03

1031 chr1:36474826 acacag acacug 2.397 8.26e-03

Protein: CPEB1(Hs/Mm)

Sequence Position Genomic Coordinate Motif K-mer Z-score P-value

597 chr1:36474392 uuuuu uuuuu 3.297 4.89e-04

598 chr1:36474393 uuuuu uuuuu 3.297 4.89e-04

599 chr1:36474394 uuuuu uuuuu 3.297 4.89e-04

600 chr1:36474395 uuuuu uuuuu 3.297 4.89e-04

601 chr1:36474396 uuuuu uuuuu 3.297 4.89e-04

602 chr1:36474397 uuuuu uuuuu 3.297 4.89e-04

603 chr1:36474398 uuuuu uuuuu 3.297 4.89e-04

604 chr1:36474399 uuuuu uuuuu 3.297 4.89e-04

605 chr1:36474400 uuuuu uuuuu 3.297 4.89e-04

606 chr1:36474401 uuuuu uuuuu 3.297 4.89e-04

607 chr1:36474402 uuuuu uuuuu 3.297 4.89e-04

608 chr1:36474403 uuuuu uuuuu 3.297 4.89e-04

609 chr1:36474404 uuuuu uuuuu 3.297 4.89e-04

610 chr1:36474405 uuuuu uuuuu 3.297 4.89e-04

611 chr1:36474406 uuuuu uuuuu 3.297 4.89e-04

612 chr1:36474407 uuuuu uuuuu 3.297 4.89e-04

613 chr1:36474408 uuuuu uuuuu 3.297 4.89e-04

614 chr1:36474409 uuuuu uuuuu 3.297 4.89e-04

615 chr1:36474410 uuuuu uuuuu 3.297 4.89e-04

616 chr1:36474411 uuuuu uuuuu 3.297 4.89e-04

617 chr1:36474412 uuuuu uuuug 2.797 2.58e-03

849 chr1:36474644 uuuuu ucuuu 2.406 8.06e-03

851 chr1:36474646 uuuuu uuugu 2.406 8.06e-03

852 chr1:36474647 uuuuu uuguu 2.406 8.06e-03

853 chr1:36474648 uuuuu uguuu 2.406 8.06e-03

854 chr1:36474649 uuuuu guuuu 2.406 8.06e-03

855 chr1:36474650 uuuuu uuuuu 2.971 1.48e-03

856 chr1:36474651 uuuuu uuuuu 2.971 1.48e-03

857 chr1:36474652 uuuuu uuuuc 2.594 4.74e-03

Protein: CPEB2(Hs/Mm)

Sequence Position Genomic Coordinate Motif K-mer Z-score P-value

597 chr1:36474392 chuuuuu uuuuuuu 3.694 1.10e-04

598 chr1:36474393 chuuuuu uuuuuuu 3.694 1.10e-04

599 chr1:36474394 chuuuuu uuuuuuu 3.694 1.10e-04

600 chr1:36474395 chuuuuu uuuuuuu 3.694 1.10e-04

601 chr1:36474396 chuuuuu uuuuuuu 3.694 1.10e-04

602 chr1:36474397 chuuuuu uuuuuuu 3.694 1.10e-04

603 chr1:36474398 chuuuuu uuuuuuu 3.694 1.10e-04

604 chr1:36474399 chuuuuu uuuuuuu 3.694 1.10e-04

605 chr1:36474400 chuuuuu uuuuuuu 3.694 1.10e-04

606 chr1:36474401 chuuuuu uuuuuuu 3.694 1.10e-04

607 chr1:36474402 chuuuuu uuuuuuu 3.694 1.10e-04

608 chr1:36474403 chuuuuu uuuuuuu 3.694 1.10e-04

609 chr1:36474404 chuuuuu uuuuuuu 3.694 1.10e-04

610 chr1:36474405 chuuuuu uuuuuuu 3.694 1.10e-04

611 chr1:36474406 chuuuuu uuuuuuu 3.694 1.10e-04

612 chr1:36474407 chuuuuu uuuuuuu 3.694 1.10e-04

613 chr1:36474408 chuuuuu uuuuuuu 3.694 1.10e-04

614 chr1:36474409 chuuuuu uuuuuuu 3.694 1.10e-04

615 chr1:36474410 chuuuuu uuuuuug 3.327 4.39e-04

850 chr1:36474645 chuuuuu cuuuguu 2.867 2.07e-03

852 chr1:36474647 chuuuuu uuguuuu 2.571 5.07e-03

853 chr1:36474648 chuuuuu uguuuuu 2.765 2.85e-03

854 chr1:36474649 chuuuuu guuuuuu 2.939 1.65e-03

855 chr1:36474650 chuuuuu uuuuuuc 2.643 4.11e-03

Protein: CPEB4(Hs/Mm)

Sequence Position Genomic Coordinate Motif K-mer Z-score P-value

597 chr1:36474392 uuuuuu uuuuuu 3.609 1.54e-04

598 chr1:36474393 uuuuuu uuuuuu 3.609 1.54e-04

599 chr1:36474394 uuuuuu uuuuuu 3.609 1.54e-04

600 chr1:36474395 uuuuuu uuuuuu 3.609 1.54e-04

601 chr1:36474396 uuuuuu uuuuuu 3.609 1.54e-04

602 chr1:36474397 uuuuuu uuuuuu 3.609 1.54e-04

603 chr1:36474398 uuuuuu uuuuuu 3.609 1.54e-04

604 chr1:36474399 uuuuuu uuuuuu 3.609 1.54e-04

605 chr1:36474400 uuuuuu uuuuuu 3.609 1.54e-04

606 chr1:36474401 uuuuuu uuuuuu 3.609 1.54e-04

607 chr1:36474402 uuuuuu uuuuuu 3.609 1.54e-04

608 chr1:36474403 uuuuuu uuuuuu 3.609 1.54e-04

609 chr1:36474404 uuuuuu uuuuuu 3.609 1.54e-04

610 chr1:36474405 uuuuuu uuuuuu 3.609 1.54e-04

611 chr1:36474406 uuuuuu uuuuuu 3.609 1.54e-04

612 chr1:36474407 uuuuuu uuuuuu 3.609 1.54e-04

613 chr1:36474408 uuuuuu uuuuuu 3.609 1.54e-04

614 chr1:36474409 uuuuuu uuuuuu 3.609 1.54e-04

615 chr1:36474410 uuuuuu uuuuuu 3.609 1.54e-04

616 chr1:36474411 uuuuuu uuuuug 3.026 1.24e-03

850 chr1:36474645 uuuuuu cuuugu 2.817 2.42e-03

853 chr1:36474648 uuuuuu uguuuu 2.774 2.77e-03

854 chr1:36474649 uuuuuu guuuuu 3.174 7.52e-04

855 chr1:36474650 uuuuuu uuuuuu 3.191 7.09e-04

856 chr1:36474651 uuuuuu uuuuuc 2.704 3.43e-03

Protein: DAZ3(Hs/Mm)

Sequence Position Genomic Coordinate Motif K-mer Z-score P-value

368 chr1:36474163 uuguuu guguuu 1.971 2.44e-02

375 chr1:36474170 aguuuu aguuuu 1.741 4.08e-02

597 chr1:36474392 uuguuu uuuuuu 3.529 2.09e-04

598 chr1:36474393 uuguuu uuuuuu 3.529 2.09e-04

599 chr1:36474394 uuguuu uuuuuu 3.529 2.09e-04

600 chr1:36474395 uuguuu uuuuuu 3.529 2.09e-04

601 chr1:36474396 uuguuu uuuuuu 3.529 2.09e-04

602 chr1:36474397 uuguuu uuuuuu 3.529 2.09e-04

603 chr1:36474398 uuguuu uuuuuu 3.529 2.09e-04

604 chr1:36474399 uuguuu uuuuuu 3.529 2.09e-04

605 chr1:36474400 uuguuu uuuuuu 3.529 2.09e-04

606 chr1:36474401 uuguuu uuuuuu 3.529 2.09e-04

607 chr1:36474402 uuguuu uuuuuu 3.529 2.09e-04

608 chr1:36474403 uuguuu uuuuuu 3.529 2.09e-04

609 chr1:36474404 uuguuu uuuuuu 3.529 2.09e-04

610 chr1:36474405 uuguuu uuuuuu 3.529 2.09e-04

611 chr1:36474406 uuguuu uuuuuu 3.529 2.09e-04

612 chr1:36474407 uuguuu uuuuuu 3.529 2.09e-04

613 chr1:36474408 uuguuu uuuuuu 3.529 2.09e-04

614 chr1:36474409 uuguuu uuuuuu 3.529 2.09e-04

615 chr1:36474410 uuguuu uuuuuu 3.529 2.09e-04

616 chr1:36474411 uuguuu uuuuug 2.952 1.58e-03

848 chr1:36474643 uuguuu gucuuu 2.971 1.48e-03

852 chr1:36474647 uuguuu uuguuu 3.471 2.59e-04

854 chr1:36474649 uuguuu guuuuu 3.212 6.59e-04

855 chr1:36474650 uuguuu uuuuuu 3.375 3.69e-04

856 chr1:36474651 uuguuu uuuuuc 2.894 1.90e-03

Protein: DAZAP1(Hs/Mm)

Sequence Position Genomic Coordinate Motif K-mer Z-score P-value

104 chr1:36473899 uagkwwr ugggaag 1.716 4.31e-02

1151 chr1:36474946 uauauag gauauca 1.917 2.76e-02

Protein: ELAVL4(Hs/Mm)

Sequence Position Genomic Coordinate Motif K-mer Z-score P-value

398 chr1:36474193 uuauu ucauu 2.303 1.06e-02

421 chr1:36474216 uaauu uuauu 2.212 1.35e-02

422 chr1:36474217 uaauu uauuu 2.212 1.35e-02

426 chr1:36474221 uaauu uaacu 1.770 3.84e-02

597 chr1:36474392 uuauu uuuuu 3.339 4.20e-04

598 chr1:36474393 uuauu uuuuu 3.339 4.20e-04

599 chr1:36474394 uuauu uuuuu 3.339 4.20e-04

600 chr1:36474395 uuauu uuuuu 3.339 4.20e-04

601 chr1:36474396 uuauu uuuuu 3.339 4.20e-04

602 chr1:36474397 uuauu uuuuu 3.339 4.20e-04

603 chr1:36474398 uuauu uuuuu 3.339 4.20e-04

604 chr1:36474399 uuauu uuuuu 3.339 4.20e-04

605 chr1:36474400 uuauu uuuuu 3.339 4.20e-04

606 chr1:36474401 uuauu uuuuu 3.339 4.20e-04

607 chr1:36474402 uuauu uuuuu 3.339 4.20e-04

608 chr1:36474403 uuauu uuuuu 3.339 4.20e-04

609 chr1:36474404 uuauu uuuuu 3.339 4.20e-04

610 chr1:36474405 uuauu uuuuu 3.339 4.20e-04

611 chr1:36474406 uuauu uuuuu 3.339 4.20e-04

612 chr1:36474407 uuauu uuuuu 3.339 4.20e-04

613 chr1:36474408 uuauu uuuuu 3.339 4.20e-04

614 chr1:36474409 uuauu uuuuu 3.339 4.20e-04

615 chr1:36474410 uuauu uuuuu 3.339 4.20e-04

616 chr1:36474411 uuauu uuuuu 3.339 4.20e-04

852 chr1:36474647 uuauu uuguu 2.936 1.66e-03

855 chr1:36474650 uuauu uuuuu 3.092 9.94e-04

856 chr1:36474651 uuauu uuuuu 3.092 9.94e-04

863 chr1:36474658 uuauu uuaug 2.642 4.12e-03

Protein: ENOX1(Hs/Mm)

Sequence Position Genomic Coordinate Motif K-mer Z-score P-value

1634 chr1:36475429 hrkacag caggcag 2.156 1.55e-02

Protein: ESRP1(Hs/Mm)

Sequence Position Genomic Coordinate Motif K-mer Z-score P-value

85 chr1:36473880 gggugg uggagg 2.159 1.54e-02

101 chr1:36473896 gggugg gggugg 3.307 4.72e-04

253 chr1:36474048 gggugg uggggg 1.909 2.81e-02

922 chr1:36474717 gggugg gggugg 2.580 4.94e-03

925 chr1:36474720 gggugg uggggg 2.307 1.05e-02

1536 chr1:36475331 gggugg aggagg 1.875 3.04e-02

1540 chr1:36475335 gggugg gggugu 2.330 9.90e-03

1546 chr1:36475341 gggugg uggagg 2.511 6.02e-03

Protein: ESRP2(Hs/Mm)

Sequence Position Genomic Coordinate Motif K-mer Z-score P-value

74 chr1:36473869 ugggrad uggggua 2.092 1.82e-02

104 chr1:36473899 ugggrad ugggaag 2.908 1.82e-03

253 chr1:36474048 ugggrad uggggga 2.447 7.20e-03

254 chr1:36474049 ugggrad gggggaa 2.368 8.94e-03

920 chr1:36474715 ugggrad uggggug 2.434 7.47e-03

925 chr1:36474720 ugggrad uggggga 2.526 5.77e-03

926 chr1:36474721 ugggrad gggggac 2.395 8.31e-03

Protein: EWSR1(Hs/Mm)

Sequence Position Genomic Coordinate Motif K-mer Z-score P-value

10 chr1:36473805 aggug agguc 2.679 3.69e-03

50 chr1:36473845 aggug agguu 1.991 2.32e-02

101 chr1:36473896 gggggggg ggguggga 2.366 8.99e-03

922 chr1:36474717 gggggggg gggugggg 3.324 4.44e-04

923 chr1:36474718 gggggggg gguggggg 3.070 1.07e-03

Protein: FUBP1(Hs/Mm)

Sequence Position Genomic Coordinate Motif K-mer Z-score P-value

362 chr1:36474157 uauguau aguguag 1.667 4.78e-02

367 chr1:36474162 uauguau aguguuu 1.667 4.78e-02

597 chr1:36474392 uauuu uuuuu 3.304 4.77e-04

598 chr1:36474393 uauuu uuuuu 3.304 4.77e-04

599 chr1:36474394 uauuu uuuuu 3.304 4.77e-04

600 chr1:36474395 uauuu uuuuu 3.304 4.77e-04

601 chr1:36474396 uauuu uuuuu 3.304 4.77e-04

602 chr1:36474397 uauuu uuuuu 3.304 4.77e-04

603 chr1:36474398 uauuu uuuuu 3.304 4.77e-04

604 chr1:36474399 uauuu uuuuu 3.304 4.77e-04

605 chr1:36474400 uauuu uuuuu 3.304 4.77e-04

606 chr1:36474401 uauuu uuuuu 3.304 4.77e-04

607 chr1:36474402 uauuu uuuuu 3.304 4.77e-04

608 chr1:36474403 uauuu uuuuu 3.304 4.77e-04

609 chr1:36474404 uauuu uuuuu 3.304 4.77e-04

610 chr1:36474405 uauuu uuuuu 3.304 4.77e-04

611 chr1:36474406 uauuu uuuuu 3.304 4.77e-04

612 chr1:36474407 uauuu uuuuu 3.304 4.77e-04

613 chr1:36474408 uauuu uuuuu 3.304 4.77e-04

614 chr1:36474409 uauuu uuuuu 3.304 4.77e-04

615 chr1:36474410 uauuu uuuuu 3.304 4.77e-04

616 chr1:36474411 uauuu uuuuu 3.304 4.77e-04

849 chr1:36474644 uauuu ucuuu 2.784 2.68e-03

853 chr1:36474648 uauuu uguuu 2.960 1.54e-03

855 chr1:36474650 uauuu uuuuu 3.008 1.31e-03

856 chr1:36474651 uauuu uuuuu 3.008 1.31e-03

Protein: FUBP3(Hs/Mm)

Sequence Position Genomic Coordinate Motif K-mer Z-score P-value

397 chr1:36474192 uuaau uucau 1.770 3.84e-02

420 chr1:36474215 uuuau guuau 1.793 3.65e-02

421 chr1:36474216 uuaau uuauu 1.770 3.84e-02

597 chr1:36474392 uuuau uuuuu 3.240 5.98e-04

598 chr1:36474393 uuuau uuuuu 3.240 5.98e-04

599 chr1:36474394 uuuau uuuuu 3.240 5.98e-04

600 chr1:36474395 uuuau uuuuu 3.240 5.98e-04

601 chr1:36474396 uuuau uuuuu 3.240 5.98e-04

602 chr1:36474397 uuuau uuuuu 3.240 5.98e-04

603 chr1:36474398 uuuau uuuuu 3.240 5.98e-04

604 chr1:36474399 uuuau uuuuu 3.240 5.98e-04

605 chr1:36474400 uuuau uuuuu 3.240 5.98e-04

606 chr1:36474401 uuuau uuuuu 3.240 5.98e-04

607 chr1:36474402 uuuau uuuuu 3.240 5.98e-04

608 chr1:36474403 uuuau uuuuu 3.240 5.98e-04

609 chr1:36474404 uuuau uuuuu 3.240 5.98e-04

610 chr1:36474405 uuuau uuuuu 3.240 5.98e-04

611 chr1:36474406 uuuau uuuuu 3.240 5.98e-04

612 chr1:36474407 uuuau uuuuu 3.240 5.98e-04

613 chr1:36474408 uuuau uuuuu 3.240 5.98e-04

614 chr1:36474409 uuuau uuuuu 3.240 5.98e-04

615 chr1:36474410 uuuau uuuuu 3.240 5.98e-04

616 chr1:36474411 uuuau uuuuu 3.240 5.98e-04

851 chr1:36474646 uuuau uuugu 2.727 3.20e-03

855 chr1:36474650 uuuau uuuuu 2.975 1.46e-03

856 chr1:36474651 uuuau uuuuu 2.975 1.46e-03

862 chr1:36474657 uuuau cuuau 2.612 4.50e-03

Protein: FUS(Hs/Mm)

Sequence Position Genomic Coordinate Motif K-mer Z-score P-value

102 chr1:36473897 ggggg ggugg 2.355 9.26e-03

1586 chr1:36475381 cgcgc ugcgc 2.120 1.70e-02

1588 chr1:36475383 cgcgc cgcgc 1.952 2.55e-02

Protein: G3BP2(Hs/Mm)

Sequence Position Genomic Coordinate Motif K-mer Z-score P-value

50 chr1:36473845 aggaudr agguuca 2.507 6.09e-03

63 chr1:36473858 aggaudr aggagag 1.773 3.81e-02

68 chr1:36473863 aggaudr agguugu 1.760 3.92e-02

1536 chr1:36475331 aggaudr aggaggg 2.653 3.99e-03

1539 chr1:36475334 aggaudr agggugu 2.520 5.87e-03

1561 chr1:36475356 aggaudr uggaugg 2.333 9.82e-03

1617 chr1:36475412 aggaudr aggcugu 1.973 2.42e-02

1642 chr1:36475437 aggaudr aggagag 1.733 4.15e-02

Protein: HNRNPA0(Hs/Mm)

Sequence Position Genomic Coordinate Motif K-mer Z-score P-value

400 chr1:36474195 auuagu auuagc 1.718 4.29e-02

1022 chr1:36474817 uauag uuuag 2.876 2.01e-03

1024 chr1:36474819 uauag uagag 2.090 1.83e-02

Protein: HNRNPA1(Hs/Mm)

Sequence Position Genomic Coordinate Motif K-mer Z-score P-value

64 chr1:36473859 guaguagu ggagaggu 2.803 2.53e-03

67 chr1:36473862 guaguagu gagguugu 2.459 6.97e-03

73 chr1:36473868 duagggw guggggu 1.932 2.67e-02

86 chr1:36473881 duagggw ggagggu 1.932 2.67e-02

244 chr1:36474039 rgnyag gggcag 2.109 1.75e-02

362 chr1:36474157 guaguagu aguguagu 2.590 4.80e-03

365 chr1:36474160 guaguagu guaguguu 2.738 3.09e-03

1553 chr1:36475348 guaguagu agaguagu 3.213 6.57e-04

1556 chr1:36475351 guaguagu guaguugg 3.066 1.08e-03

1635 chr1:36475430 rgnyag aggcag 2.164 1.52e-02

1647 chr1:36475442 rgnyag agaaag 2.109 1.75e-02

Protein: HNRNPA1L2(Hs/Mm)

Sequence Position Genomic Coordinate Motif K-mer Z-score P-value

73 chr1:36473868 duagggw guggggu 2.620 4.40e-03

86 chr1:36473881 duagggw ggagggu 2.141 1.61e-02

919 chr1:36474714 duagggw cuggggu 1.803 3.57e-02

Protein: HNRNPA2B1(Hs/Mm)

Sequence Position Genomic Coordinate Motif K-mer Z-score P-value

60 chr1:36473855 gguaguag ggcaggag 2.323 1.01e-02

73 chr1:36473868 duagggw guggggu 1.867 3.10e-02

74 chr1:36473869 ggggg ugggg 2.078 1.89e-02

76 chr1:36473871 gggua gggua 2.752 2.96e-03

86 chr1:36473881 duagggw ggagggu 1.960 2.50e-02

89 chr1:36473884 gggua ggguc 2.752 2.96e-03

101 chr1:36473896 gggua gggug 2.990 1.39e-03

105 chr1:36473900 gggua gggaa 2.276 1.14e-02

244 chr1:36474039 gggua gggca 1.800 3.59e-02

253 chr1:36474048 ggggg ugggg 1.853 3.19e-02

254 chr1:36474049 ggggg ggggg 2.093 1.82e-02

255 chr1:36474050 gggua gggga 1.800 3.59e-02

256 chr1:36474051 gggua gggaa 1.800 3.59e-02

463 chr1:36474258 gggua gggga 1.800 3.59e-02

465 chr1:36474260 gggua ggaua 1.800 3.59e-02

920 chr1:36474715 ggggg ugggg 2.605 4.59e-03

922 chr1:36474717 gggua gggug 1.800 3.59e-02

925 chr1:36474720 ggggg ugggg 2.605 4.59e-03

926 chr1:36474721 ggggg ggggg 2.845 2.22e-03

927 chr1:36474722 gggua gggga 1.800 3.59e-02

1533 chr1:36475328 gguaguag ggcaggag 3.554 1.90e-04

1555 chr1:36475350 gguaguag aguaguug 3.138 8.51e-04

1636 chr1:36475431 gguaguag ggcagcag 1.985 2.36e-02

Protein: HNRNPC(Hs/Mm)

Sequence Position Genomic Coordinate Motif K-mer Z-score P-value

369 chr1:36474164 uuuuu uguuu 1.747 4.03e-02

597 chr1:36474392 huuuuuk uuuuuuu 3.643 1.35e-04

598 chr1:36474393 huuuuuk uuuuuuu 3.643 1.35e-04

599 chr1:36474394 huuuuuk uuuuuuu 3.643 1.35e-04

600 chr1:36474395 huuuuuk uuuuuuu 3.643 1.35e-04

601 chr1:36474396 huuuuuk uuuuuuu 3.643 1.35e-04

602 chr1:36474397 huuuuuk uuuuuuu 3.643 1.35e-04

603 chr1:36474398 huuuuuk uuuuuuu 3.643 1.35e-04

604 chr1:36474399 huuuuuk uuuuuuu 3.643 1.35e-04

605 chr1:36474400 huuuuuk uuuuuuu 3.643 1.35e-04

606 chr1:36474401 huuuuuk uuuuuuu 3.643 1.35e-04

607 chr1:36474402 huuuuuk uuuuuuu 3.643 1.35e-04

608 chr1:36474403 huuuuuk uuuuuuu 3.643 1.35e-04

609 chr1:36474404 huuuuuk uuuuuuu 3.643 1.35e-04

610 chr1:36474405 huuuuuk uuuuuuu 3.643 1.35e-04

611 chr1:36474406 huuuuuk uuuuuuu 3.643 1.35e-04

612 chr1:36474407 huuuuuk uuuuuuu 3.643 1.35e-04

613 chr1:36474408 huuuuuk uuuuuuu 3.643 1.35e-04

614 chr1:36474409 huuuuuk uuuuuuu 3.643 1.35e-04

615 chr1:36474410 huuuuuk uuuuuug 3.482 2.49e-04

616 chr1:36474411 uuuuu uuuuu 3.363 3.85e-04

617 chr1:36474412 uuuuu uuuug 2.678 3.70e-03

849 chr1:36474644 uuuuu ucuuu 2.521 5.85e-03

850 chr1:36474645 huuuuuk cuuuguu 2.768 2.82e-03

851 chr1:36474646 huuuuuk uuuguuu 2.759 2.90e-03

852 chr1:36474647 huuuuuk uuguuuu 2.759 2.90e-03

853 chr1:36474648 huuuuuk uguuuuu 2.759 2.90e-03

854 chr1:36474649 huuuuuk guuuuuu 3.125 8.89e-04

855 chr1:36474650 uuuuu uuuuu 3.041 1.18e-03

856 chr1:36474651 uuuuu uuuuu 3.041 1.18e-03

857 chr1:36474652 uuuuu uuuuc 2.507 6.09e-03

Protein: HNRNPCL1(Hs/Mm)

Sequence Position Genomic Coordinate Motif K-mer Z-score P-value

369 chr1:36474164 uuuuu uguuu 1.925 2.71e-02

597 chr1:36474392 huuuuuk uuuuuuu 3.675 1.19e-04

598 chr1:36474393 huuuuuk uuuuuuu 3.675 1.19e-04

599 chr1:36474394 huuuuuk uuuuuuu 3.675 1.19e-04

600 chr1:36474395 huuuuuk uuuuuuu 3.675 1.19e-04

601 chr1:36474396 huuuuuk uuuuuuu 3.675 1.19e-04

602 chr1:36474397 huuuuuk uuuuuuu 3.675 1.19e-04

603 chr1:36474398 huuuuuk uuuuuuu 3.675 1.19e-04

604 chr1:36474399 huuuuuk uuuuuuu 3.675 1.19e-04

605 chr1:36474400 huuuuuk uuuuuuu 3.675 1.19e-04

606 chr1:36474401 huuuuuk uuuuuuu 3.675 1.19e-04

607 chr1:36474402 huuuuuk uuuuuuu 3.675 1.19e-04

608 chr1:36474403 huuuuuk uuuuuuu 3.675 1.19e-04

609 chr1:36474404 huuuuuk uuuuuuu 3.675 1.19e-04

610 chr1:36474405 huuuuuk uuuuuuu 3.675 1.19e-04

611 chr1:36474406 huuuuuk uuuuuuu 3.675 1.19e-04

612 chr1:36474407 huuuuuk uuuuuuu 3.675 1.19e-04

613 chr1:36474408 huuuuuk uuuuuuu 3.675 1.19e-04

614 chr1:36474409 huuuuuk uuuuuuu 3.675 1.19e-04

615 chr1:36474410 uuuuu uuuuu 3.564 1.83e-04

616 chr1:36474411 uuuuu uuuuu 3.564 1.83e-04

617 chr1:36474412 uuuuu uuuug 2.985 1.42e-03

849 chr1:36474644 uuuuu ucuuu 2.722 3.24e-03

850 chr1:36474645 huuuuuk cuuuguu 2.772 2.79e-03

851 chr1:36474646 huuuuuk uuuguuu 2.781 2.71e-03

852 chr1:36474647 huuuuuk uuguuuu 2.781 2.71e-03

853 chr1:36474648 huuuuuk uguuuuu 2.781 2.71e-03

854 chr1:36474649 huuuuuk guuuuuu 3.123 8.95e-04

855 chr1:36474650 uuuuu uuuuu 3.233 6.12e-04

856 chr1:36474651 uuuuu uuuuu 3.233 6.12e-04

857 chr1:36474652 uuuuu uuuuc 2.797 2.58e-03

Protein: HNRNPD(Hs/Mm)

Sequence Position Genomic Coordinate Motif K-mer Z-score P-value

399 chr1:36474194 uauuaa cauuag 1.768 3.85e-02

422 chr1:36474217 uauuaa uauuua 1.866 3.10e-02

1143 chr1:36474938 uauuaa uguuac 2.085 1.85e-02

1153 chr1:36474948 uauuaa uaucau 2.280 1.13e-02

Protein: HNRNPDL(Hs/Mm)

Sequence Position Genomic Coordinate Motif K-mer Z-score P-value

421 chr1:36474216 uaauu uuauu 1.943 2.60e-02

422 chr1:36474217 uaauu uauuu 1.943 2.60e-02

426 chr1:36474221 uaauu uaacu 1.686 4.59e-02

457 chr1:36474252 uaaaa uaaaa 2.066 1.94e-02

Protein: HNRNPF(Hs/Mm)

Sequence Position Genomic Coordinate Motif K-mer Z-score P-value

2 chr1:36473797 agggu ugggu 1.847 3.24e-02

44 chr1:36473839 gukgykg guuguga 2.098 1.80e-02

67 chr1:36473862 gukgykg gagguug 2.934 1.67e-03

70 chr1:36473865 gukgykg guugugg 2.705 3.42e-03

71 chr1:36473866 gggagggg uugugggg 1.864 3.12e-02

74 chr1:36473869 agggu ugggg 2.245 1.24e-02

75 chr1:36473870 agggu ggggu 2.878 2.00e-03

80 chr1:36473875 gukgykg auggcug 2.951 1.58e-03

88 chr1:36473883 agggu agggu 3.541 1.99e-04

99 chr1:36473894 gugkau gugggu 1.804 3.56e-02

100 chr1:36473895 agggu ugggu 3.735 9.39e-05

101 chr1:36473896 gggagggg ggguggga 2.515 5.95e-03

104 chr1:36473899 agggu uggga 3.490 2.42e-04

117 chr1:36473912 agggu agggc 3.112 9.29e-04

243 chr1:36474038 agggu agggc 1.918 2.76e-02

253 chr1:36474048 agggu ugggg 1.888 2.95e-02

920 chr1:36474715 agggu ugggg 2.378 8.70e-03

921 chr1:36474716 agggu ggggu 2.408 8.02e-03

922 chr1:36474717 gggagggg gggugggg 3.152 8.11e-04

923 chr1:36474718 gggagggg gguggggg 2.955 1.56e-03

924 chr1:36474719 gukgykg guggggg 2.689 3.58e-03

925 chr1:36474720 agggu ugggg 2.378 8.70e-03

1556 chr1:36475351 gukgykg guaguug 3.541 1.99e-04

1560 chr1:36475355 gugkau uuggau 2.321 1.01e-02

1564 chr1:36475359 gukgykg auggcug 3.213 6.57e-04

1661 chr1:36475456 gukgykg gugguug 3.033 1.21e-03

Protein: HNRNPH1(Hs/Mm)

Sequence Position Genomic Coordinate Motif K-mer Z-score P-value

1551 chr1:36475346 gargag gcagag 2.320 1.02e-02

1643 chr1:36475438 gargag ggagag 1.930 2.68e-02

Protein: HNRNPH2(Hs/Mm)

Sequence Position Genomic Coordinate Motif K-mer Z-score P-value

60 chr1:36473855 gggaggg ggcagga 2.633 4.23e-03

85 chr1:36473880 gggaggg uggaggg 3.233 6.12e-04

101 chr1:36473896 gggaggg ggguggg 3.433 2.98e-04

105 chr1:36473900 gggaggg gggaaga 2.856 2.15e-03

114 chr1:36473909 gggaggg gugaggg 2.956 1.56e-03

240 chr1:36474035 gggaggg ggaaggg 2.178 1.47e-02

252 chr1:36474047 gggggg cugggg 2.220 1.32e-02

253 chr1:36474048 gggggg uggggg 2.319 1.02e-02

256 chr1:36474051 gggaggg gggaagc 1.944 2.59e-02

919 chr1:36474714 gggggg cugggg 3.121 9.01e-04

922 chr1:36474717 gggaggg ggguggg 2.611 4.51e-03

924 chr1:36474719 gggggg gugggg 3.286 5.08e-04

925 chr1:36474720 gggggg uggggg 3.264 5.49e-04

1533 chr1:36475328 gggaggg ggcagga 2.044 2.05e-02

1536 chr1:36475331 gggaggg aggaggg 2.689 3.58e-03

1546 chr1:36475341 gggaggg uggaggc 2.811 2.47e-03

1570 chr1:36475365 gggaggg ggaagga 2.233 1.28e-02

Protein: HNRNPK(Hs/Mm)

Sequence Position Genomic Coordinate Motif K-mer Z-score P-value

1589 chr1:36475384 gccca gcgca 1.655 4.90e-02

1594 chr1:36475389 gccca gcuca 1.655 4.90e-02

1604 chr1:36475399 gccca gucca 2.336 9.75e-03

1625 chr1:36475420 gccca gcccc 1.655 4.90e-02

1626 chr1:36475421 gccca cccca 1.655 4.90e-02

Protein: HNRNPL(Hs/Mm)

Sequence Position Genomic Coordinate Motif K-mer Z-score P-value

522 chr1:36474317 acacrav acaugaa 2.268 1.17e-02

1014 chr1:36474809 acacrav acacugg 2.563 5.19e-03

1030 chr1:36474825 uacaca cacacu 2.474 6.68e-03

1031 chr1:36474826 acacrav acacugc 2.549 5.40e-03

Protein: HNRNPM(Hs/Mm)

Sequence Position Genomic Coordinate Motif K-mer Z-score P-value

39 chr1:36473834 gguugguu ugcugguu 2.905 1.84e-03

1558 chr1:36475353 gguugguu aguuggau 3.127 8.83e-04

1562 chr1:36475357 gguugguu ggauggcu 3.127 8.83e-04

1659 chr1:36475454 gguugguu gugugguu 2.905 1.84e-03

Protein: HNRNPU(Hs/Mm)

Sequence Position Genomic Coordinate Motif K-mer Z-score P-value

77 chr1:36473872 uguauug gguaugg 1.844 3.26e-02

364 chr1:36474159 uguauug uguagug 1.812 3.50e-02

1541 chr1:36475336 uguauug gguguug 2.484 6.50e-03

Protein: HuR(Hs/Mm)

Sequence Position Genomic Coordinate Motif K-mer Z-score P-value

372 chr1:36474167 uukruuu uugaguu 1.769 3.84e-02

373 chr1:36474168 uukruuu ugaguuu 1.648 4.97e-02

597 chr1:36474392 uukruuu uuuuuuu 3.363 3.85e-04

598 chr1:36474393 uukruuu uuuuuuu 3.363 3.85e-04

599 chr1:36474394 uukruuu uuuuuuu 3.363 3.85e-04

600 chr1:36474395 uukruuu uuuuuuu 3.363 3.85e-04

601 chr1:36474396 uukruuu uuuuuuu 3.363 3.85e-04

602 chr1:36474397 uukruuu uuuuuuu 3.363 3.85e-04

603 chr1:36474398 uukruuu uuuuuuu 3.363 3.85e-04

604 chr1:36474399 uukruuu uuuuuuu 3.363 3.85e-04

605 chr1:36474400 uukruuu uuuuuuu 3.363 3.85e-04

606 chr1:36474401 uukruuu uuuuuuu 3.363 3.85e-04

607 chr1:36474402 uukruuu uuuuuuu 3.363 3.85e-04

608 chr1:36474403 uukruuu uuuuuuu 3.363 3.85e-04

609 chr1:36474404 uukruuu uuuuuuu 3.363 3.85e-04

610 chr1:36474405 uukruuu uuuuuuu 3.363 3.85e-04

611 chr1:36474406 uukruuu uuuuuuu 3.363 3.85e-04

612 chr1:36474407 uukruuu uuuuuuu 3.363 3.85e-04

613 chr1:36474408 uukruuu uuuuuuu 3.363 3.85e-04

614 chr1:36474409 uukruuu uuuuuuu 3.363 3.85e-04

851 chr1:36474646 uukruuu uuuguuu 3.187 7.19e-04

852 chr1:36474647 uukruuu uuguuuu 3.110 9.35e-04

854 chr1:36474649 uukruuu guuuuuu 2.516 5.93e-03

Protein: ILF2(Hs/Mm)

Sequence Position Genomic Coordinate Motif K-mer Z-score P-value

76 chr1:36473871 gggua gggua 2.752 2.96e-03

89 chr1:36473884 gggua ggguc 2.752 2.96e-03

101 chr1:36473896 gggugggg ggguggga 3.109 9.39e-04

105 chr1:36473900 gggua gggaa 2.276 1.14e-02

244 chr1:36474039 gggua gggca 1.800 3.59e-02

255 chr1:36474050 gggua gggga 1.800 3.59e-02

256 chr1:36474051 gggua gggaa 1.800 3.59e-02

463 chr1:36474258 gggua gggga 1.800 3.59e-02

465 chr1:36474260 gggua ggaua 1.800 3.59e-02

922 chr1:36474717 gggugggg gggugggg 2.719 3.27e-03

923 chr1:36474718 gggugggg gguggggg 2.359 9.16e-03

927 chr1:36474722 gggua gggga 1.800 3.59e-02

Protein: KHDRBS1(Hs/Mm)

Sequence Position Genomic Coordinate Motif K-mer Z-score P-value

456 chr1:36474251 auaaaav guaaaau 2.082 1.87e-02

Protein: KHDRBS2(Hs/Mm)

Sequence Position Genomic Coordinate Motif K-mer Z-score P-value

455 chr1:36474250 aauaaa uguaaa 1.954 2.54e-02

466 chr1:36474261 rauaaam gauaaug 1.871 3.07e-02

Protein: KHDRBS3(Hs/Mm)

Sequence Position Genomic Coordinate Motif K-mer Z-score P-value

455 chr1:36474250 aauaaa uguaaa 2.067 1.94e-02

466 chr1:36474261 auaaav gauaau 2.160 1.54e-02

Protein: KHSRP(Hs/Mm)

Sequence Position Genomic Coordinate Motif K-mer Z-score P-value

421 chr1:36474216 uuuuu uuauu 1.779 3.76e-02

597 chr1:36474392 uuuuu uuuuu 3.282 5.15e-04

598 chr1:36474393 uuuuu uuuuu 3.282 5.15e-04

599 chr1:36474394 uuuuu uuuuu 3.282 5.15e-04

600 chr1:36474395 uuuuu uuuuu 3.282 5.15e-04

601 chr1:36474396 uuuuu uuuuu 3.282 5.15e-04

602 chr1:36474397 uuuuu uuuuu 3.282 5.15e-04

603 chr1:36474398 uuuuu uuuuu 3.282 5.15e-04

604 chr1:36474399 uuuuu uuuuu 3.282 5.15e-04

605 chr1:36474400 uuuuu uuuuu 3.282 5.15e-04

606 chr1:36474401 uuuuu uuuuu 3.282 5.15e-04

607 chr1:36474402 uuuuu uuuuu 3.282 5.15e-04

608 chr1:36474403 uuuuu uuuuu 3.282 5.15e-04

609 chr1:36474404 uuuuu uuuuu 3.282 5.15e-04

610 chr1:36474405 uuuuu uuuuu 3.282 5.15e-04

611 chr1:36474406 uuuuu uuuuu 3.282 5.15e-04

612 chr1:36474407 uuuuu uuuuu 3.282 5.15e-04

613 chr1:36474408 uuuuu uuuuu 3.282 5.15e-04

614 chr1:36474409 uuuuu uuuuu 3.282 5.15e-04

615 chr1:36474410 uuuuu uuuuu 3.282 5.15e-04

616 chr1:36474411 uuuuu uuuuu 3.282 5.15e-04

852 chr1:36474647 uuuuu uuguu 2.725 3.22e-03

853 chr1:36474648 uguau uguuu 2.271 1.16e-02

855 chr1:36474650 uuuuu uuuuu 2.977 1.46e-03

856 chr1:36474651 uuuuu uuuuu 2.977 1.46e-03

Protein: LIN28A(Hs/Mm)

Sequence Position Genomic Coordinate Motif K-mer Z-score P-value

63 chr1:36473858 hggagwa aggagag 2.270 1.16e-02

Protein: MATR3(Hs/Mm)

Sequence Position Genomic Coordinate Motif K-mer Z-score P-value

871 chr1:36474666 maucuur cagcuug 2.486 6.46e-03

888 chr1:36474683 maucuur cauccug 2.446 7.22e-03

Protein: MBNL1(Hs/Mm)

Sequence Position Genomic Coordinate Motif K-mer Z-score P-value

94 chr1:36473889 gcuugc ugcaug 2.013 2.21e-02

119 chr1:36473914 gcuugc ggcuuu 2.208 1.36e-02

244 chr1:36474039 gcgcagc gggcagc 3.056 1.12e-03

1114 chr1:36474909 ugcua ggcua 2.132 1.65e-02

1143 chr1:36474938 ugcua uguua 2.132 1.65e-02

1150 chr1:36474945 ugcua ugaua 2.368 8.94e-03

1589 chr1:36475384 gcgcagc gcgcagc 1.917 2.76e-02

1635 chr1:36475430 gcgcagc aggcagc 2.264 1.18e-02

Protein: MSI1(Hs/Mm)

Sequence Position Genomic Coordinate Motif K-mer Z-score P-value

897 chr1:36474692 uaguwrg caguuug 1.848 3.23e-02

909 chr1:36474704 uaguwrg aaguuug 1.848 3.23e-02

Protein: NOVA1(Hs/Mm)

Sequence Position Genomic Coordinate Motif K-mer Z-score P-value

379 chr1:36474174 uucauaa uucaucc 2.567 5.13e-03

397 chr1:36474192 uucauaa uucauua 2.667 3.83e-03

449 chr1:36474244 uucauaa cucaucu 2.133 1.65e-02

Protein: NUPL2(Hs/Mm)

Sequence Position Genomic Coordinate Motif K-mer Z-score P-value

458 chr1:36474253 caaagg aaaaug 2.409 8.00e-03

Protein: PABPC4(Hs/Mm)

Sequence Position Genomic Coordinate Motif K-mer Z-score P-value

834 chr1:36474629 aaaaaar aaacaag 1.909 2.81e-02

Protein: PABPN1(Hs/Mm)

Sequence Position Genomic Coordinate Motif K-mer Z-score P-value

1642 chr1:36475437 araaga aggaga 1.704 4.42e-02

Protein: PABPN1L(Hs/Mm)

Sequence Position Genomic Coordinate Motif K-mer Z-score P-value

458 chr1:36474253 aaaaa aaaau 2.283 1.12e-02

Protein: PCBP2(Hs/Mm)

Sequence Position Genomic Coordinate Motif K-mer Z-score P-value

956 chr1:36474751 uuccc cuccc 1.890 2.94e-02

Protein: PCBP4(Hs/Mm)

Sequence Position Genomic Coordinate Motif K-mer Z-score P-value

1 chr1:36473796 gucgg guggg 1.971 2.44e-02

91 chr1:36473886 gucgg gucug 2.461 6.93e-03

99 chr1:36473894 gucgg guggg 1.971 2.44e-02

103 chr1:36473898 gucgg guggg 2.461 6.93e-03

Protein: PPRC1(Hs/Mm)

Sequence Position Genomic Coordinate Motif K-mer Z-score P-value

1585 chr1:36475380 ssgcgcs cugcgcg 1.705 4.41e-02

1587 chr1:36475382 ssgcgcs gcgcgca 1.744 4.06e-02

Protein: PRR3(Hs/Mm)

Sequence Position Genomic Coordinate Motif K-mer Z-score P-value

400 chr1:36474195 auuac auuag 2.157 1.55e-02

1138 chr1:36474933 auuac acuac 1.750 4.01e-02

1144 chr1:36474939 auuac guuac 1.750 4.01e-02

Protein: PTB3(Hs/Mm)

Sequence Position Genomic Coordinate Motif K-mer Z-score P-value

850 chr1:36474645 cuuucu cuuugu 2.185 1.44e-02

857 chr1:36474652 cuuucu uuuucc 2.160 1.54e-02

Protein: PTBP3(Hs/Mm)

Sequence Position Genomic Coordinate Motif K-mer Z-score P-value

850 chr1:36474645 cuuucu cuuugu 2.213 1.34e-02

857 chr1:36474652 cuuucu uuuucc 2.188 1.43e-02

Protein: PUF60(Hs/Mm)

Sequence Position Genomic Coordinate Motif K-mer Z-score P-value

597 chr1:36474392 uuuuu uuuuu 3.403 3.33e-04

598 chr1:36474393 uuuuu uuuuu 3.403 3.33e-04

599 chr1:36474394 uuuuu uuuuu 3.403 3.33e-04

600 chr1:36474395 uuuuu uuuuu 3.403 3.33e-04

601 chr1:36474396 uuuuu uuuuu 3.403 3.33e-04

602 chr1:36474397 uuuuu uuuuu 3.403 3.33e-04

603 chr1:36474398 uuuuu uuuuu 3.403 3.33e-04

604 chr1:36474399 uuuuu uuuuu 3.403 3.33e-04

605 chr1:36474400 uuuuu uuuuu 3.403 3.33e-04

606 chr1:36474401 uuuuu uuuuu 3.403 3.33e-04

607 chr1:36474402 uuuuu uuuuu 3.403 3.33e-04

608 chr1:36474403 uuuuu uuuuu 3.403 3.33e-04

609 chr1:36474404 uuuuu uuuuu 3.403 3.33e-04

610 chr1:36474405 uuuuu uuuuu 3.403 3.33e-04

611 chr1:36474406 uuuuu uuuuu 3.403 3.33e-04

612 chr1:36474407 uuuuu uuuuu 3.403 3.33e-04

613 chr1:36474408 uuuuu uuuuu 3.403 3.33e-04

614 chr1:36474409 uuuuu uuuuu 3.403 3.33e-04

615 chr1:36474410 uuuuu uuuuu 3.403 3.33e-04

616 chr1:36474411 uuuuu uuuuu 3.403 3.33e-04

630 chr1:36474425 ucucu ucuca 1.897 2.89e-02

632 chr1:36474427 ucucu ucacu 1.897 2.89e-02

634 chr1:36474429 ucucu acucu 1.897 2.89e-02

636 chr1:36474431 ucucu ucugu 1.897 2.89e-02

851 chr1:36474646 uuuuu uuugu 2.882 1.98e-03

855 chr1:36474650 uuuuu uuuuu 3.076 1.05e-03

856 chr1:36474651 uuuuu uuuuu 3.076 1.05e-03

Protein: PUM1(Hs/Mm)

Sequence Position Genomic Coordinate Motif K-mer Z-score P-value

457 chr1:36474252 uaaau uaaaa 2.195 1.41e-02

458 chr1:36474253 uaaau aaaau 2.195 1.41e-02

Protein: PUM2(Hs/Mm)

Sequence Position Genomic Coordinate Motif K-mer Z-score P-value

455 chr1:36474250 uguanaua uguaaaau 2.034 2.10e-02

Protein: RALY(Hs/Mm)

Sequence Position Genomic Coordinate Motif K-mer Z-score P-value

597 chr1:36474392 uuuuuub uuuuuuu 3.955 3.83e-05

598 chr1:36474393 uuuuuub uuuuuuu 3.955 3.83e-05

599 chr1:36474394 uuuuuub uuuuuuu 3.955 3.83e-05

600 chr1:36474395 uuuuuub uuuuuuu 3.955 3.83e-05

601 chr1:36474396 uuuuuub uuuuuuu 3.955 3.83e-05

602 chr1:36474397 uuuuuub uuuuuuu 3.955 3.83e-05

603 chr1:36474398 uuuuuub uuuuuuu 3.955 3.83e-05

604 chr1:36474399 uuuuuub uuuuuuu 3.955 3.83e-05

605 chr1:36474400 uuuuuub uuuuuuu 3.955 3.83e-05

606 chr1:36474401 uuuuuub uuuuuuu 3.955 3.83e-05

607 chr1:36474402 uuuuuub uuuuuuu 3.955 3.83e-05

608 chr1:36474403 uuuuuub uuuuuuu 3.955 3.83e-05

609 chr1:36474404 uuuuuub uuuuuuu 3.955 3.83e-05

610 chr1:36474405 uuuuuub uuuuuuu 3.955 3.83e-05

611 chr1:36474406 uuuuuub uuuuuuu 3.955 3.83e-05

612 chr1:36474407 uuuuuub uuuuuuu 3.955 3.83e-05

613 chr1:36474408 uuuuuub uuuuuuu 3.955 3.83e-05

614 chr1:36474409 uuuuuub uuuuuuu 3.955 3.83e-05

615 chr1:36474410 uuuuuub uuuuuug 4.062 2.43e-05

616 chr1:36474411 uuuuu uuuuu 3.487 2.44e-04

851 chr1:36474646 uuuuuub uuuguuu 2.902 1.85e-03

852 chr1:36474647 uuuuu uuguu 2.913 1.79e-03

853 chr1:36474648 uuuuuub uguuuuu 2.902 1.85e-03

854 chr1:36474649 uuuuuub guuuuuu 3.009 1.31e-03

855 chr1:36474650 uuuuuub uuuuuuc 3.259 5.59e-04

856 chr1:36474651 uuuuu uuuuu 3.183 7.29e-04

Protein: RBFOX1(Hs/Mm)

Sequence Position Genomic Coordinate Motif K-mer Z-score P-value

1639 chr1:36475434 wgcaugm agcagga 2.355 9.26e-03

Protein: RBFOX2(Hs/Mm)

Sequence Position Genomic Coordinate Motif K-mer Z-score P-value

54 chr1:36473849 gcaug ucaug 1.649 4.96e-02

61 chr1:36473856 gcaug gcagg 1.730 4.18e-02

78 chr1:36473873 gcaug guaug 2.495 6.30e-03

1640 chr1:36475435 gcaug gcagg 1.802 3.58e-02

1653 chr1:36475448 gcaug gccug 1.694 4.51e-02

Protein: RBFOX3(Hs/Mm)

Sequence Position Genomic Coordinate Motif K-mer Z-score P-value

54 chr1:36473849 gcaug ucaug 1.664 4.81e-02

61 chr1:36473856 gcaug gcagg 1.745 4.05e-02

78 chr1:36473873 gcaug guaug 2.518 5.90e-03

1640 chr1:36475435 gcaug gcagg 1.827 3.38e-02

1653 chr1:36475448 gcaug gccug 1.709 4.37e-02

Protein: RBM15B(Hs/Mm)

Sequence Position Genomic Coordinate Motif K-mer Z-score P-value

424 chr1:36474219 uuuaa uuuaa 1.653 4.92e-02

597 chr1:36474392 uuuuauuu uuuuuuuu 3.931 4.23e-05

598 chr1:36474393 uuuuauuu uuuuuuuu 3.931 4.23e-05

599 chr1:36474394 uuuuauuu uuuuuuuu 3.931 4.23e-05

600 chr1:36474395 uuuuauuu uuuuuuuu 3.931 4.23e-05

601 chr1:36474396 uuuuauuu uuuuuuuu 3.931 4.23e-05

602 chr1:36474397 uuuuauuu uuuuuuuu 3.931 4.23e-05

603 chr1:36474398 uuuuauuu uuuuuuuu 3.931 4.23e-05

604 chr1:36474399 uuuuauuu uuuuuuuu 3.931 4.23e-05

605 chr1:36474400 uuuuauuu uuuuuuuu 3.931 4.23e-05

606 chr1:36474401 uuuuauuu uuuuuuuu 3.931 4.23e-05

607 chr1:36474402 uuuuauuu uuuuuuuu 3.931 4.23e-05

608 chr1:36474403 uuuuauuu uuuuuuuu 3.931 4.23e-05

609 chr1:36474404 uuuuauuu uuuuuuuu 3.931 4.23e-05

610 chr1:36474405 uuuuauuu uuuuuuuu 3.931 4.23e-05

611 chr1:36474406 uuuuauuu uuuuuuuu 3.931 4.23e-05

612 chr1:36474407 uuuuauuu uuuuuuuu 3.931 4.23e-05

613 chr1:36474408 uuuuauuu uuuuuuuu 3.931 4.23e-05

614 chr1:36474409 uuuuauuu uuuuuuug 3.681 1.16e-04

615 chr1:36474410 uuuuauuu uuuuuuga 3.319 4.52e-04

850 chr1:36474645 uuuuauuu cuuuguuu 2.625 4.33e-03

851 chr1:36474646 uuuuauuu uuuguuuu 2.653 3.99e-03

852 chr1:36474647 uuuuauuu uuguuuuu 2.722 3.24e-03

853 chr1:36474648 uuuuauuu uguuuuuu 2.778 2.73e-03

854 chr1:36474649 uuuuauuu guuuuuuc 2.681 3.67e-03

855 chr1:36474650 uuuuauuu uuuuuucc 2.736 3.11e-03

857 chr1:36474652 uuuuauuu uuuuccuu 2.708 3.38e-03

900 chr1:36474695 uuuaa uuuga 2.076 1.89e-02

901 chr1:36474696 uuuaa uugaa 2.076 1.89e-02

912 chr1:36474707 uuuaa uuuga 2.076 1.89e-02

913 chr1:36474708 uuuaa uugaa 2.076 1.89e-02

Protein: RBM24(Hs/Mm)

Sequence Position Genomic Coordinate Motif K-mer Z-score P-value

78 chr1:36473873 gugug guaug 2.720 3.26e-03

91 chr1:36473886 gugug gucug 3.220 6.41e-04

98 chr1:36473893 wgwgugd ugugggu 3.442 2.89e-04

100 chr1:36473895 wgwgugd ugggugg 3.688 1.13e-04

101 chr1:36473896 gugug gggug 3.340 4.19e-04

102 chr1:36473897 wgwgugd gguggga 3.545 1.96e-04

109 chr1:36473904 wgwgugd agagugu 4.052 2.54e-05

110 chr1:36473905 gugug gagug 3.420 3.13e-04

111 chr1:36473906 wgwgugd aguguga 4.364 6.39e-06

112 chr1:36473907 gugug gugug 3.740 9.20e-05

254 chr1:36474049 ggggg ggggg 2.292 1.10e-02

362 chr1:36474157 wgwgugd aguguag 2.429 7.57e-03

363 chr1:36474158 gugug gugua 2.090 1.83e-02

367 chr1:36474162 wgwgugd aguguuu 2.494 6.32e-03

368 chr1:36474163 gugug guguu 2.200 1.39e-02

369 chr1:36474164 wgwgugd uguuuga 2.571 5.07e-03

370 chr1:36474165 gugug guuug 2.120 1.70e-02

597 chr1:36474392 uuuuuuuuu uuuuuuuuu 4.339 7.16e-06

598 chr1:36474393 uuuuuuuuu uuuuuuuuu 4.339 7.16e-06

599 chr1:36474394 uuuuuuuuu uuuuuuuuu 4.339 7.16e-06

600 chr1:36474395 uuuuuuuuu uuuuuuuuu 4.339 7.16e-06

601 chr1:36474396 uuuuuuuuu uuuuuuuuu 4.339 7.16e-06

602 chr1:36474397 uuuuuuuuu uuuuuuuuu 4.339 7.16e-06

603 chr1:36474398 uuuuuuuuu uuuuuuuuu 4.339 7.16e-06

604 chr1:36474399 uuuuuuuuu uuuuuuuuu 4.339 7.16e-06

605 chr1:36474400 uuuuuuuuu uuuuuuuuu 4.339 7.16e-06

606 chr1:36474401 uuuuuuuuu uuuuuuuuu 4.339 7.16e-06

607 chr1:36474402 uuuuuuuuu uuuuuuuuu 4.339 7.16e-06

608 chr1:36474403 uuuuuuuuu uuuuuuuuu 4.339 7.16e-06

609 chr1:36474404 uuuuuuuuu uuuuuuuuu 4.339 7.16e-06

610 chr1:36474405 uuuuuuuuu uuuuuuuuu 4.339 7.16e-06

611 chr1:36474406 uuuuuuuuu uuuuuuuuu 4.339 7.16e-06

612 chr1:36474407 uuuuuuuuu uuuuuuuuu 4.339 7.16e-06

613 chr1:36474408 uuuuuuuuu uuuuuuuug 3.935 4.16e-05

614 chr1:36474409 uuuuuuuuu uuuuuuuga 3.532 2.06e-04

849 chr1:36474644 uuuuuuuuu ucuuuguuu 2.871 2.05e-03

850 chr1:36474645 uuuuuuuuu cuuuguuuu 3.161 7.86e-04

851 chr1:36474646 uuuuuuuuu uuuguuuuu 3.226 6.28e-04

852 chr1:36474647 uuuuuuuuu uuguuuuuu 3.226 6.28e-04

853 chr1:36474648 uuuuuuuuu uguuuuuuc 2.871 2.05e-03

855 chr1:36474650 uuuuuuuuu uuuuuuccu 2.968 1.50e-03

856 chr1:36474651 uuuuuuuuu uuuuuccuu 2.968 1.50e-03

857 chr1:36474652 uuuuuuuuu uuuuccuua 2.952 1.58e-03

858 chr1:36474653 uuuuuuuuu uuuccuuau 2.871 2.05e-03

898 chr1:36474693 wgwgugd aguuuga 2.299 1.08e-02

899 chr1:36474694 gugug guuug 2.470 6.76e-03

911 chr1:36474706 gugug guuug 2.470 6.76e-03

922 chr1:36474717 gugug gggug 2.470 6.76e-03

926 chr1:36474721 ggggg ggggg 3.038 1.19e-03

1656 chr1:36475451 wgwgugd ugagugu 2.909 1.81e-03

1657 chr1:36475452 gugug gagug 2.960 1.54e-03

1658 chr1:36475453 wgwgugd agugugg 3.130 8.74e-04

1659 chr1:36475454 gugug gugug 3.280 5.19e-04

Protein: RBM25(Hs/Mm)

Sequence Position Genomic Coordinate Motif K-mer Z-score P-value

254 chr1:36474049 ggggag ggggga 2.103 1.77e-02

255 chr1:36474050 ggggag ggggaa 2.126 1.68e-02

921 chr1:36474716 ggggag ggggug 2.931 1.69e-03

926 chr1:36474721 ggggag ggggga 2.759 2.90e-03

927 chr1:36474722 ggggag ggggac 2.793 2.61e-03

Protein: RBM28(Hs/Mm)

Sequence Position Genomic Coordinate Motif K-mer Z-score P-value

1554 chr1:36475349 gwguagd gaguagu 2.167 1.51e-02

Protein: RBM3(Hs/Mm)

Sequence Position Genomic Coordinate Motif K-mer Z-score P-value

903 chr1:36474698 radacka gaaagga 1.982 2.37e-02

Protein: RBM38(Hs/Mm)

Sequence Position Genomic Coordinate Motif K-mer Z-score P-value

45 chr1:36473840 kkguguk uugugag 2.077 1.89e-02

71 chr1:36473866 kkguguk uuguggg 2.692 3.55e-03

97 chr1:36473892 kkguguk auguggg 3.538 2.02e-04

99 chr1:36473894 kkguguk gugggug 3.756 8.63e-05

101 chr1:36473896 kkguguk ggguggg 3.641 1.36e-04

110 chr1:36473905 kkguguk gagugug 3.833 6.33e-05

112 chr1:36473907 kkguguk gugugag 3.833 6.33e-05

922 chr1:36474717 kkguguk ggguggg 2.103 1.77e-02

924 chr1:36474719 kkguguk guggggg 1.923 2.72e-02

1540 chr1:36475335 kkguguk ggguguu 2.128 1.67e-02

1657 chr1:36475452 kkguguk gagugug 3.244 5.89e-04

1659 chr1:36475454 kkguguk guguggu 3.115 9.20e-04

Protein: RBM4(Hs/Mm)

Sequence Position Genomic Coordinate Motif K-mer Z-score P-value

1587 chr1:36475382 gcgcgu gcgcgc 2.096 1.80e-02

1589 chr1:36475384 gcgcgu gcgcag 1.822 3.42e-02

Protein: RBM41(Hs/Mm)

Sequence Position Genomic Coordinate Motif K-mer Z-score P-value

396 chr1:36474191 wuacwuk auucauu 1.926 2.71e-02

1116 chr1:36474911 wuacwuk cuacaua 2.025 2.14e-02

1140 chr1:36474935 uacuu uacug 2.500 6.21e-03

1146 chr1:36474941 uacuu uaccu 2.433 7.49e-03

Protein: RBM45(Hs/Mm)

Sequence Position Genomic Coordinate Motif K-mer Z-score P-value

1014 chr1:36474809 acacc acacu 1.748 4.02e-02

1031 chr1:36474826 acacc acacu 1.748 4.02e-02

1138 chr1:36474933 acgac acuac 2.340 9.64e-03

Protein: RBM47(Hs/Mm)

Sequence Position Genomic Coordinate Motif K-mer Z-score P-value

398 chr1:36474193 ugauu ucauu 2.092 1.82e-02

1662 chr1:36475457 ugauu ugguu 2.092 1.82e-02

Protein: RBM4B(Hs/Mm)

Sequence Position Genomic Coordinate Motif K-mer Z-score P-value

1587 chr1:36475382 gcgcgg gcgcgc 1.674 4.71e-02

Protein: RBM5(Hs/Mm)

Sequence Position Genomic Coordinate Motif K-mer Z-score P-value

103 chr1:36473898 garggwr gugggaa 1.705 4.41e-02

241 chr1:36474036 garggwr gaagggc 2.026 2.14e-02

254 chr1:36474049 garggwr gggggaa 2.346 9.49e-03

258 chr1:36474053 garggwr gaagcua 2.359 9.16e-03

1534 chr1:36475329 garggwr gcaggag 1.872 3.06e-02

1538 chr1:36475333 garggwr gagggug 1.859 3.15e-02

1571 chr1:36475366 garggwr gaaggac 2.449 7.16e-03

Protein: RBM8A(Hs/Mm)

Sequence Position Genomic Coordinate Motif K-mer Z-score P-value

1585 chr1:36475380 rygcgcb cugcgcg 1.878 3.02e-02

1587 chr1:36475382 rygcgcb gcgcgca 2.027 2.13e-02

Protein: RBMS1(Hs/Mm)

Sequence Position Genomic Coordinate Motif K-mer Z-score P-value

1151 chr1:36474946 kauauas gauauca 1.890 2.94e-02

Protein: RBMS2(Hs/Mm)

Sequence Position Genomic Coordinate Motif K-mer Z-score P-value

1152 chr1:36474947 auauaa auauca 2.571 5.07e-03

Protein: RBMS3(Hs/Mm)

Sequence Position Genomic Coordinate Motif K-mer Z-score P-value

1150 chr1:36474945 auauau ugauau 2.250 1.22e-02

1151 chr1:36474946 hauaua gauauc 1.937 2.64e-02

Protein: SAMD4A(Hs/Mm)

Sequence Position Genomic Coordinate Motif K-mer Z-score P-value

40 chr1:36473835 gckgghm gcugguu 2.284 1.12e-02

61 chr1:36473856 gckgghm gcaggag 2.420 7.76e-03

1615 chr1:36475410 gckgghm gcaggcu 2.062 1.96e-02

Protein: SF1(Hs/Mm)

Sequence Position Genomic Coordinate Motif K-mer Z-score P-value

456 chr1:36474251 uuaaca guaaaa 1.854 3.19e-02

Protein: SFPQ(Hs/Mm)

Sequence Position Genomic Coordinate Motif K-mer Z-score P-value

355 chr1:36474150 uagug cagug 2.250 1.22e-02

361 chr1:36474156 uagug cagug 2.250 1.22e-02

366 chr1:36474161 uagug uagug 2.731 3.16e-03

408 chr1:36474203 uguaag ugugau 1.687 4.58e-02

455 chr1:36474250 uguaag uguaaa 1.892 2.92e-02

902 chr1:36474697 uguaag ugaaag 1.771 3.83e-02

1140 chr1:36474935 uagug uacug 1.769 3.84e-02

1661 chr1:36475456 kurrukk gugguug 2.037 2.08e-02

Protein: SNRPA(Hs/Mm)

Sequence Position Genomic Coordinate Motif K-mer Z-score P-value

876 chr1:36474671 wugcacr uggcaca 1.817 3.46e-02

877 chr1:36474672 ugcaca ggcaca 1.658 4.87e-02

Protein: SRSF1(Hs/Mm)

Sequence Position Genomic Coordinate Motif K-mer Z-score P-value

86 chr1:36473881 gragga ggaggg 2.946 1.61e-03

106 chr1:36473901 gragga ggaaga 2.699 3.48e-03

241 chr1:36474036 gragga gaaggg 1.925 2.71e-02

254 chr1:36474049 gragga ggggga 1.978 2.40e-02

926 chr1:36474721 gragga ggggga 2.441 7.32e-03

946 chr1:36474741 gragga agagga 2.032 2.11e-02

1534 chr1:36475329 gragga gcagga 2.462 6.91e-03

1537 chr1:36475332 gragga ggaggg 2.957 1.55e-03

1547 chr1:36475342 gragga ggaggc 3.301 4.82e-04

1571 chr1:36475366 gragga gaagga 2.914 1.78e-03

Protein: SRSF10(Hs/Mm)

Sequence Position Genomic Coordinate Motif K-mer Z-score P-value

244 chr1:36474039 cagcag gggcag 2.425 7.65e-03

1549 chr1:36475344 cagcag aggcag 2.562 5.20e-03

1589 chr1:36475384 cagcag gcgcag 1.650 4.95e-02

1613 chr1:36475408 cagcag cugcag 3.450 2.80e-04

1635 chr1:36475430 cagcag aggcag 3.062 1.10e-03

1638 chr1:36475433 cagcag cagcag 3.475 2.55e-04

1643 chr1:36475438 agagavm ggagaga 2.398 8.24e-03

1645 chr1:36475440 agagavm agagaaa 2.892 1.91e-03

Protein: SRSF11(Hs/Mm)

Sequence Position Genomic Coordinate Motif K-mer Z-score P-value

63 chr1:36473858 agggg aggag 1.811 3.51e-02

66 chr1:36473861 agggg agagg 1.802 3.58e-02

Protein: SRSF2(Hs/Mm)

Sequence Position Genomic Coordinate Motif K-mer Z-score P-value

63 chr1:36473858 ggagwd aggaga 1.897 2.89e-02

85 chr1:36473880 ggagwd uggagg 2.322 1.01e-02

245 chr1:36474040 agcagc ggcagc 2.648 4.05e-03

1536 chr1:36475331 ggagwd aggagg 2.322 1.01e-02

1546 chr1:36475341 ggagwd uggagg 2.092 1.82e-02

1590 chr1:36475385 agcagc cgcagc 1.648 4.97e-02

1605 chr1:36475400 uccag uccag 2.391 8.40e-03

1614 chr1:36475409 agcagc ugcagg 3.318 4.53e-04

1627 chr1:36475422 uccag cccag 2.391 8.40e-03

1636 chr1:36475431 agcagc ggcagc 2.830 2.33e-03

1639 chr1:36475434 agcagc agcagg 2.920 1.75e-03

Protein: SRSF4(Hs/Mm)

Sequence Position Genomic Coordinate Motif K-mer Z-score P-value

1533 chr1:36475328 agcag ggcag 1.842 3.27e-02

1536 chr1:36475331 agcag aggag 1.842 3.27e-02

1550 chr1:36475345 agcag ggcag 1.842 3.27e-02

1555 chr1:36475350 agcag aguag 1.842 3.27e-02

1614 chr1:36475409 agcag ugcag 2.281 1.13e-02

1636 chr1:36475431 agcag ggcag 2.281 1.13e-02

1639 chr1:36475434 agcag agcag 2.719 3.27e-03

1642 chr1:36475437 agcag aggag 1.842 3.27e-02

Protein: SRSF5(Hs/Mm)

Sequence Position Genomic Coordinate Motif K-mer Z-score P-value

246 chr1:36474041 gcagc gcagc 2.763 2.86e-03

258 chr1:36474053 gcagc gaagc 2.325 1.00e-02

1591 chr1:36475386 gcagc gcagc 2.325 1.00e-02

1615 chr1:36475410 gcagc gcagg 2.544 5.48e-03

1637 chr1:36475432 gcagc gcagc 2.325 1.00e-02

1640 chr1:36475435 gcagc gcagg 1.886 2.96e-02

Protein: SRSF8(Hs/Mm)

Sequence Position Genomic Coordinate Motif K-mer Z-score P-value

36 chr1:36473831 agcagc ggcugc 2.085 1.85e-02

245 chr1:36474040 agcagc ggcagc 2.707 3.39e-03

1590 chr1:36475385 agcagc cgcagc 1.695 4.50e-02

1614 chr1:36475409 agcagc ugcagg 3.159 7.92e-04

1636 chr1:36475431 agcagc ggcagc 2.756 2.93e-03

1639 chr1:36475434 agcagc agcagg 2.561 5.22e-03

Protein: SRSF9(Hs/Mm)

Sequence Position Genomic Coordinate Motif K-mer Z-score P-value

63 chr1:36473858 aggag aggag 1.683 4.62e-02

82 chr1:36473877 kgrwgsm ggcugga 3.000 1.35e-03

85 chr1:36473880 aggag uggag 2.356 9.24e-03

101 chr1:36473896 kgrwgsm ggguggg 2.803 2.53e-03

240 chr1:36474035 kgrwgsm ggaaggg 1.958 2.51e-02

244 chr1:36474039 gggaa gggca 2.377 8.73e-03

255 chr1:36474050 gggaa gggga 2.377 8.73e-03

256 chr1:36474051 gggaa gggaa 2.816 2.43e-03

903 chr1:36474698 kgrwgsm gaaagga 1.845 3.25e-02

922 chr1:36474717 kgrwgsm ggguggg 1.817 3.46e-02

961 chr1:36474756 aggag uggag 2.129 1.66e-02

1533 chr1:36475328 kgrwgsm ggcagga 2.169 1.50e-02

1536 chr1:36475331 aggag aggag 1.683 4.62e-02

1546 chr1:36475341 aggag uggag 2.347 9.46e-03

1547 chr1:36475342 kgrwgsm ggaggca 3.324 4.44e-04

1562 chr1:36475357 kgrwgsm ggauggc 3.408 3.27e-04

1566 chr1:36475361 kgrwgsm ggcugga 3.352 4.01e-04

1570 chr1:36475365 kgrwgsm ggaagga 3.704 1.06e-04

Protein: TAF15(Hs/Mm)

Sequence Position Genomic Coordinate Motif K-mer Z-score P-value

72 chr1:36473867 ggggggg ugugggg 2.035 2.09e-02

76 chr1:36473871 gggua gggua 2.752 2.96e-03

89 chr1:36473884 gggua ggguc 2.752 2.96e-03

101 chr1:36473896 gggua gggug 2.990 1.39e-03

105 chr1:36473900 gggua gggaa 2.276 1.14e-02

244 chr1:36474039 gggua gggca 1.800 3.59e-02

251 chr1:36474046 ggggggg ccugggg 1.894 2.91e-02

252 chr1:36474047 ggggggg cuggggg 1.953 2.54e-02

255 chr1:36474050 gggua gggga 1.800 3.59e-02

256 chr1:36474051 gggua gggaa 1.800 3.59e-02

463 chr1:36474258 gggua gggga 1.800 3.59e-02

465 chr1:36474260 gggua ggaua 1.800 3.59e-02

918 chr1:36474713 ggggggg acugggg 3.012 1.30e-03

922 chr1:36474717 gggua gggug 1.800 3.59e-02

923 chr1:36474718 ggggggg ggugggg 3.341 4.17e-04

924 chr1:36474719 ggggggg guggggg 3.271 5.36e-04

927 chr1:36474722 gggua gggga 1.800 3.59e-02

Protein: TARDBP(Hs/Mm)

Sequence Position Genomic Coordinate Motif K-mer Z-score P-value

78 chr1:36473873 guaug guaug 3.051 1.14e-03

87 chr1:36473882 gagug gaggg 2.761 2.88e-03

91 chr1:36473886 guaug gucug 3.173 7.54e-04

95 chr1:36473890 guaug gcaug 3.122 8.98e-04

101 chr1:36473896 gagug gggug 2.881 1.98e-03

110 chr1:36473905 gagug gagug 3.330 4.34e-04

112 chr1:36473907 guaug gugug 3.031 1.22e-03

116 chr1:36473911 gagug gaggg 2.761 2.88e-03

355 chr1:36474150 gagug cagug 2.073 1.91e-02

361 chr1:36474156 gagug cagug 2.073 1.91e-02

366 chr1:36474161 gagug uagug 2.073 1.91e-02

374 chr1:36474169 gagug gaguu 2.073 1.91e-02

899 chr1:36474694 guaug guuug 1.837 3.31e-02

911 chr1:36474706 guaug guuug 1.837 3.31e-02

1644 chr1:36475439 gagug gagag 2.073 1.91e-02

1657 chr1:36475452 gagug gagug 2.991 1.39e-03

1659 chr1:36475454 gagug gugug 2.532 5.67e-03

Protein: TIA1(Hs/Mm)

Sequence Position Genomic Coordinate Motif K-mer Z-score P-value

369 chr1:36474164 uuuuu uguuu 1.924 2.72e-02

377 chr1:36474172 uuuuu uuuuc 1.674 4.71e-02

597 chr1:36474392 uuuuubk uuuuuuu 3.922 4.39e-05

598 chr1:36474393 uuuuubk uuuuuuu 3.922 4.39e-05

599 chr1:36474394 uuuuubk uuuuuuu 3.922 4.39e-05

600 chr1:36474395 uuuuubk uuuuuuu 3.922 4.39e-05

601 chr1:36474396 uuuuubk uuuuuuu 3.922 4.39e-05

602 chr1:36474397 uuuuubk uuuuuuu 3.922 4.39e-05

603 chr1:36474398 uuuuubk uuuuuuu 3.922 4.39e-05

604 chr1:36474399 uuuuubk uuuuuuu 3.922 4.39e-05

605 chr1:36474400 uuuuubk uuuuuuu 3.922 4.39e-05

606 chr1:36474401 uuuuubk uuuuuuu 3.922 4.39e-05

607 chr1:36474402 uuuuubk uuuuuuu 3.922 4.39e-05

608 chr1:36474403 uuuuubk uuuuuuu 3.922 4.39e-05

609 chr1:36474404 uuuuubk uuuuuuu 3.922 4.39e-05

610 chr1:36474405 uuuuubk uuuuuuu 3.922 4.39e-05

611 chr1:36474406 uuuuubk uuuuuuu 3.922 4.39e-05

612 chr1:36474407 uuuuubk uuuuuuu 3.922 4.39e-05

613 chr1:36474408 uuuuubk uuuuuuu 3.922 4.39e-05

614 chr1:36474409 uuuuubk uuuuuuu 3.922 4.39e-05

615 chr1:36474410 uuuuubk uuuuuug 4.029 2.80e-05

616 chr1:36474411 uuuuubk uuuuuga 3.539 2.01e-04

617 chr1:36474412 uuuuu uuuug 2.985 1.42e-03

849 chr1:36474644 uuuuu ucuuu 2.689 3.58e-03

851 chr1:36474646 uuuuubk uuuguuu 2.843 2.23e-03

852 chr1:36474647 uuuuubk uuguuuu 2.843 2.23e-03

853 chr1:36474648 uuuuubk uguuuuu 2.843 2.23e-03

854 chr1:36474649 uuuuubk guuuuuu 3.010 1.31e-03

855 chr1:36474650 uuuuubk uuuuuuc 3.196 6.97e-04

856 chr1:36474651 uuuuu uuuuu 3.174 7.52e-04

857 chr1:36474652 uuuuu uuuuc 2.803 2.53e-03

Protein: TRNAU1AP(Hs/Mm)

Sequence Position Genomic Coordinate Motif K-mer Z-score P-value

419 chr1:36474214 auuua aguua 2.310 1.04e-02

423 chr1:36474218 auuua auuua 1.876 3.03e-02

597 chr1:36474392 uuuuauu uuuuuuu 3.628 1.43e-04

598 chr1:36474393 uuuuauu uuuuuuu 3.628 1.43e-04

599 chr1:36474394 uuuuauu uuuuuuu 3.628 1.43e-04

600 chr1:36474395 uuuuauu uuuuuuu 3.628 1.43e-04

601 chr1:36474396 uuuuauu uuuuuuu 3.628 1.43e-04

602 chr1:36474397 uuuuauu uuuuuuu 3.628 1.43e-04

603 chr1:36474398 uuuuauu uuuuuuu 3.628 1.43e-04

604 chr1:36474399 uuuuauu uuuuuuu 3.628 1.43e-04

605 chr1:36474400 uuuuauu uuuuuuu 3.628 1.43e-04

606 chr1:36474401 uuuuauu uuuuuuu 3.628 1.43e-04

607 chr1:36474402 uuuuauu uuuuuuu 3.628 1.43e-04

608 chr1:36474403 uuuuauu uuuuuuu 3.628 1.43e-04

609 chr1:36474404 uuuuauu uuuuuuu 3.628 1.43e-04

610 chr1:36474405 uuuuauu uuuuuuu 3.628 1.43e-04

611 chr1:36474406 uuuuauu uuuuuuu 3.628 1.43e-04

612 chr1:36474407 uuuuauu uuuuuuu 3.628 1.43e-04

613 chr1:36474408 uuuuauu uuuuuuu 3.628 1.43e-04

614 chr1:36474409 uuuuauu uuuuuuu 3.628 1.43e-04

615 chr1:36474410 uuuuauu uuuuuug 3.384 3.57e-04

616 chr1:36474411 uuuuauu uuuuuga 3.047 1.16e-03

851 chr1:36474646 uuuuauu uuuguuu 2.488 6.42e-03

852 chr1:36474647 uuuuauu uuguuuu 2.488 6.42e-03

853 chr1:36474648 uuuuauu uguuuuu 2.616 4.45e-03

854 chr1:36474649 uuuuauu guuuuuu 2.674 3.75e-03

855 chr1:36474650 uuuuauu uuuuuuc 2.802 2.54e-03

856 chr1:36474651 uuuuauu uuuuucc 2.616 4.45e-03

857 chr1:36474652 uuuuauu uuuuccu 2.547 5.43e-03

Protein: U2AF2(Hs/Mm)

Sequence Position Genomic Coordinate Motif K-mer Z-score P-value

597 chr1:36474392 uuuuuyc uuuuuuu 3.581 1.71e-04

598 chr1:36474393 uuuuuyc uuuuuuu 3.581 1.71e-04

599 chr1:36474394 uuuuuyc uuuuuuu 3.581 1.71e-04

600 chr1:36474395 uuuuuyc uuuuuuu 3.581 1.71e-04

601 chr1:36474396 uuuuuyc uuuuuuu 3.581 1.71e-04

602 chr1:36474397 uuuuuyc uuuuuuu 3.581 1.71e-04

603 chr1:36474398 uuuuuyc uuuuuuu 3.581 1.71e-04

604 chr1:36474399 uuuuuyc uuuuuuu 3.581 1.71e-04

605 chr1:36474400 uuuuuyc uuuuuuu 3.581 1.71e-04

606 chr1:36474401 uuuuuyc uuuuuuu 3.581 1.71e-04

607 chr1:36474402 uuuuuyc uuuuuuu 3.581 1.71e-04

608 chr1:36474403 uuuuuyc uuuuuuu 3.581 1.71e-04

609 chr1:36474404 uuuuuyc uuuuuuu 3.581 1.71e-04

610 chr1:36474405 uuuuuyc uuuuuuu 3.581 1.71e-04

611 chr1:36474406 uuuuuyc uuuuuuu 3.581 1.71e-04

612 chr1:36474407 uuuuuyc uuuuuuu 3.581 1.71e-04

613 chr1:36474408 uuuuuyc uuuuuuu 3.581 1.71e-04

614 chr1:36474409 uuuuuyc uuuuuuu 3.581 1.71e-04

615 chr1:36474410 uuuuuyc uuuuuug 3.505 2.28e-04

616 chr1:36474411 uuuuuyc uuuuuga 3.057 1.12e-03

851 chr1:36474646 uuuuuyc uuuguuu 2.952 1.58e-03

852 chr1:36474647 uuuuuyc uuguuuu 2.952 1.58e-03

853 chr1:36474648 uuuuuyc uguuuuu 2.952 1.58e-03

854 chr1:36474649 uuuuuyc guuuuuu 3.162 7.83e-04

855 chr1:36474650 uuuuuyc uuuuuuc 3.705 1.06e-04

856 chr1:36474651 uuuuuyc uuuuucc 3.590 1.65e-04

Protein: UNK(Hs/Mm)

Sequence Position Genomic Coordinate Motif K-mer Z-score P-value

364 chr1:36474159 auuaguu uguagug 1.721 4.26e-02

1022 chr1:36474817 uauaga uuuaga 1.866 3.10e-02

Protein: YBX2(Hs/Mm)

Sequence Position Genomic Coordinate Motif K-mer Z-score P-value

835 chr1:36474630 aacawcd aacaaga 2.311 1.04e-02

838 chr1:36474633 aacawcd aagaacc 2.284 1.12e-02

841 chr1:36474636 aacawcd aaccucg 2.203 1.38e-02

Protein: ZC3H10(Hs/Mm)

Sequence Position Genomic Coordinate Motif K-mer Z-score P-value

246 chr1:36474041 ssagcgm gcagccc 2.178 1.47e-02

1584 chr1:36475379 ssagcgm ccugcgc 2.274 1.15e-02

1591 chr1:36475386 ssagcgm gcagcuc 2.356 9.24e-03

Protein: ZC3H14(Hs/Mm)

Sequence Position Genomic Coordinate Motif K-mer Z-score P-value

597 chr1:36474392 uuuduuu uuuuuuu 3.887 5.07e-05

598 chr1:36474393 uuuduuu uuuuuuu 3.887 5.07e-05

599 chr1:36474394 uuuduuu uuuuuuu 3.887 5.07e-05

600 chr1:36474395 uuuduuu uuuuuuu 3.887 5.07e-05

601 chr1:36474396 uuuduuu uuuuuuu 3.887 5.07e-05

602 chr1:36474397 uuuduuu uuuuuuu 3.887 5.07e-05

603 chr1:36474398 uuuduuu uuuuuuu 3.887 5.07e-05

604 chr1:36474399 uuuduuu uuuuuuu 3.887 5.07e-05

605 chr1:36474400 uuuduuu uuuuuuu 3.887 5.07e-05

606 chr1:36474401 uuuduuu uuuuuuu 3.887 5.07e-05

607 chr1:36474402 uuuduuu uuuuuuu 3.887 5.07e-05

608 chr1:36474403 uuuduuu uuuuuuu 3.887 5.07e-05

609 chr1:36474404 uuuduuu uuuuuuu 3.887 5.07e-05

610 chr1:36474405 uuuduuu uuuuuuu 3.887 5.07e-05

611 chr1:36474406 uuuduuu uuuuuuu 3.887 5.07e-05

612 chr1:36474407 uuuduuu uuuuuuu 3.887 5.07e-05

613 chr1:36474408 uuuduuu uuuuuuu 3.887 5.07e-05

614 chr1:36474409 uuuduuu uuuuuuu 3.887 5.07e-05

615 chr1:36474410 uuuduuu uuuuuug 3.268 5.42e-04

851 chr1:36474646 uuuduuu uuuguuu 3.454 2.76e-04

852 chr1:36474647 uuuduuu uuguuuu 3.113 9.26e-04

853 chr1:36474648 uuuduuu uguuuuu 2.938 1.65e-03

854 chr1:36474649 uuuduuu guuuuuu 3.052 1.14e-03

855 chr1:36474650 uuuduuu uuuuuuc 3.000 1.35e-03

858 chr1:36474653 uuuduuu uuuccuu 2.990 1.39e-03

Protein: ZCRB1(Hs/Mm)

Sequence Position Genomic Coordinate Motif K-mer Z-score P-value

396 chr1:36474191 auuaau auucau 1.937 2.64e-02

424 chr1:36474219 auuaau uuuaac 2.448 7.18e-03

Protein: ZFP36(Hs/Mm)

Sequence Position Genomic Coordinate Motif K-mer Z-score P-value

457 chr1:36474252 uaaua uaaaa 1.918 2.76e-02

597 chr1:36474392 uauuu uuuuu 2.276 1.14e-02

598 chr1:36474393 uauuu uuuuu 2.276 1.14e-02

599 chr1:36474394 uauuu uuuuu 2.276 1.14e-02

600 chr1:36474395 uauuu uuuuu 2.276 1.14e-02

601 chr1:36474396 uauuu uuuuu 2.276 1.14e-02

602 chr1:36474397 uauuu uuuuu 2.276 1.14e-02

603 chr1:36474398 uauuu uuuuu 2.276 1.14e-02

604 chr1:36474399 uauuu uuuuu 2.276 1.14e-02

605 chr1:36474400 uauuu uuuuu 2.276 1.14e-02

606 chr1:36474401 uauuu uuuuu 2.276 1.14e-02

607 chr1:36474402 uauuu uuuuu 2.276 1.14e-02

608 chr1:36474403 uauuu uuuuu 2.276 1.14e-02

609 chr1:36474404 uauuu uuuuu 2.276 1.14e-02

610 chr1:36474405 uauuu uuuuu 2.276 1.14e-02

611 chr1:36474406 uauuu uuuuu 2.276 1.14e-02

612 chr1:36474407 uauuu uuuuu 2.276 1.14e-02

613 chr1:36474408 uauuu uuuuu 2.276 1.14e-02

614 chr1:36474409 uauuu uuuuu 2.276 1.14e-02

615 chr1:36474410 uauuu uuuuu 2.276 1.14e-02

616 chr1:36474411 uauuu uuuuu 2.276 1.14e-02

849 chr1:36474644 uauuu ucuuu 1.888 2.95e-02

853 chr1:36474648 uauuu uguuu 1.888 2.95e-02

855 chr1:36474650 uauuu uuuuu 1.888 2.95e-02

856 chr1:36474651 uauuu uuuuu 1.888 2.95e-02

1150 chr1:36474945 uaaua ugaua 1.918 2.76e-02

Protein: ZNF326(Hs/Mm)

Sequence Position Genomic Coordinate Motif K-mer Z-score P-value

865 chr1:36474660 auucc augcc 1.667 4.78e-02

1152 chr1:36474947 cuauaa auauca 2.580 4.94e-03

Protein: ZNF638(Hs/Mm)

Sequence Position Genomic Coordinate Motif K-mer Z-score P-value

39 chr1:36473834 bguusku ugcuggu 2.508 6.07e-03

51 chr1:36473846 bguusku gguucau 2.954 1.57e-03

369 chr1:36474164 bguusku uguuuga 1.738 4.11e-02

853 chr1:36474648 bguusku uguuuuu 2.046 2.04e-02

1543 chr1:36475338 bguusku uguugga 2.185 1.44e-02
