# Supplementary material for: CSF3R-AS promotes hepatocellular carcinoma progression and sorafenib resistance through the CSF3R/JAK2/STAT3 positive feedback loop
Source: Cell Death Dis. 2025 Mar 28;16(1):217. doi: 10.1038/s41419-025-07558-4 (PMC11953311; doi:10.1038/s41419-025-07558-4)
Supplement: Supplementary file 11 — Table S6 [file 41419_2025_7558_MOESM11_ESM.docx]

Predictions for sequence: CSF3R

Calculation parameters:

Genome: Human (hg38)

Selected motifs: All Human/Mouse motifs

Stringency level: High

Conservation filter: On

======================================================================================================================

Protein: ANKHD1(Hs/Mm)

Sequence Position Motif K-mer Z-score P-value

50 agacgww ugacgag 2.217 1.33e-02

62 agacgww agaccaa 2.246 1.24e-02

Protein: BOLL(Hs/Mm)

Sequence Position Motif K-mer Z-score P-value

851 guguua auguug 2.427 7.61e-03

854 guguua uuguga 2.247 1.23e-02

2929 guguua uuguuu 2.427 7.61e-03

2950 guguua uuguaa 2.562 5.20e-03

2959 uuuguuu aguuuuu 2.645 4.08e-03

2963 uuuguuu uuugguu 2.671 3.78e-03

2964 uuuguuu uugguuu 2.645 4.08e-03

2973 uuuguuu aguuuuu 2.645 4.08e-03

2975 uuuguuu uuuuugu 2.671 3.78e-03

2976 uuuguuu uuuuguu 2.618 4.42e-03

2977 uuuguuu uuuguug 2.816 2.43e-03

2978 guguua uuguug 2.966 1.51e-03

2981 guguua uuguug 2.966 1.51e-03

2983 uuuguuu guuguuu 2.947 1.60e-03

Protein: BRUNOL4(Hs/Mm)

Sequence Position Motif K-mer Z-score P-value

416 kgugukk ugucuga 1.662 4.83e-02

833 kgugukk ugugucu 2.216 1.33e-02

1558 kgugukk uauguga 1.743 4.07e-02

1560 kgugukk ugugauu 1.757 3.95e-02

1566 kgugukk ugagugg 1.730 4.18e-02

2614 kgugukk ugugucu 2.338 9.69e-03

2616 kgugukk ugucuuu 2.122 1.69e-02

2922 kgugukk uuugugc 2.311 1.04e-02

2924 kgugukk ugugcuu 2.230 1.29e-02

2975 kgugukk uuuuugu 2.716 3.30e-03

2977 kgugukk uuuguug 2.824 2.37e-03

Protein: BRUNOL5(Hs/Mm)

Sequence Position Motif K-mer Z-score P-value

412 ugugukk cgucugu 1.788 3.69e-02

416 ugugukk ugucuga 1.813 3.49e-02

831 ugugukk acugugu 2.387 8.49e-03

833 ugugukk ugugucu 2.750 2.98e-03

1558 ugugukk uauguga 1.788 3.69e-02

1560 ugugukk ugugauu 1.863 3.12e-02

1566 ugugukk ugagugg 1.850 3.22e-02

2612 ugugukk acugugu 2.050 2.02e-02

2614 ugugukk ugugucu 2.475 6.66e-03

2616 ugugukk ugucuuu 2.237 1.26e-02

2922 ugugukk uuugugc 1.838 3.30e-02

2924 ugugukk ugugcuu 1.850 3.22e-02

2926 ugugukk ugcuugu 1.738 4.11e-02

2975 ugugukk uuuuugu 2.525 5.78e-03

Protein: BRUNOL6(Hs/Mm)

Sequence Position Motif K-mer Z-score P-value

833 ugugdkg ugugucu 2.480 6.57e-03

835 ugugdkg ugucuug 2.640 4.15e-03

852 ugugdkg uguugug 2.693 3.54e-03

979 ugugdkg ugugagc 1.853 3.19e-02

1560 ugugdkg ugugauu 2.253 1.21e-02

1566 ugugdkg ugagugg 2.120 1.70e-02

1568 ugugdkg agugggg 2.253 1.21e-02

2108 ugugdkg ucugugg 1.933 2.66e-02

2126 ugugdkg ucuguug 1.947 2.58e-02

2977 ugugdkg uuuguug 1.733 4.15e-02

Protein: CELF1(Hs/Mm)

Sequence Position Motif K-mer Z-score P-value

819 uauguuu caugucc 2.129 1.66e-02

831 aaugu acugu 1.657 4.88e-02

833 uauguuu ugugucu 3.114 9.23e-04

850 uauguuu gauguug 2.586 4.85e-03

2108 uauguuu ucugugg 1.786 3.70e-02

2928 uauguuu cuuguuu 2.114 1.73e-02

2977 uauguuu uuuguug 3.071 1.07e-03

2980 uauguuu guuguug 2.800 2.56e-03

2983 uauguuu guuguuu 2.957 1.55e-03

Protein: CNOT4(Hs/Mm)

Sequence Position Motif K-mer Z-score P-value

358 acacag ccacag 2.397 8.26e-03

360 acacag acagau 2.359 9.16e-03

382 acacag gcagag 2.103 1.77e-02

965 acacag acauaa 2.077 1.89e-02

1189 gacaga gucaga 2.696 3.51e-03

1198 gacaga gacaca 2.859 2.12e-03

1211 gacaga ggcaga 3.207 6.71e-04

1354 acacag acagag 1.756 3.95e-02

1791 acacag gcacau 2.077 1.89e-02

Protein: CPEB1(Hs/Mm)

Sequence Position Motif K-mer Z-score P-value

2920 uuuuu cuuuu 1.790 3.67e-02

2921 uuuuu uuuug 1.906 2.83e-02

2922 uuuuu uuugu 1.790 3.67e-02

2929 uuuuu uuguu 1.790 3.67e-02

2930 uuuuu uguuu 1.790 3.67e-02

2960 uuuuu guuuu 2.428 7.59e-03

2961 uuuuu uuuuu 3.014 1.29e-03

2962 uuuuu uuuug 2.645 4.08e-03

2974 uuuuu guuuu 2.428 7.59e-03

2975 uuuuu uuuuu 3.014 1.29e-03

2976 uuuuu uuuug 2.645 4.08e-03

2977 uuuuu uuugu 2.428 7.59e-03

2978 uuuuu uuguu 2.428 7.59e-03

2981 uuuuu uuguu 2.428 7.59e-03

Protein: CPEB2(Hs/Mm)

Sequence Position Motif K-mer Z-score P-value

2919 chuuuuu ccuuuug 2.276 1.14e-02

2920 chuuuuu cuuuugu 2.204 1.38e-02

2928 chuuuuu cuuguuu 2.204 1.38e-02

2959 chuuuuu aguuuuu 2.653 3.99e-03

2960 chuuuuu guuuuug 2.643 4.11e-03

2972 chuuuuu caguuuu 2.745 3.03e-03

2973 chuuuuu aguuuuu 2.653 3.99e-03

2974 chuuuuu guuuuug 2.643 4.11e-03

Protein: CPEB4(Hs/Mm)

Sequence Position Motif K-mer Z-score P-value

2919 uuuuuu ccuuuu 2.487 6.44e-03

2920 uuuuuu cuuuug 2.452 7.10e-03

2928 uuuuuu cuuguu 2.365 9.02e-03

2959 uuuuuu aguuuu 2.730 3.17e-03

2960 uuuuuu guuuuu 3.165 7.75e-04

2961 uuuuuu uuuuug 2.757 2.92e-03

2973 uuuuuu aguuuu 2.730 3.17e-03

2974 uuuuuu guuuuu 3.174 7.52e-04

2975 uuuuuu uuuuug 2.757 2.92e-03

Protein: DAZ3(Hs/Mm)

Sequence Position Motif K-mer Z-score P-value

2918 acguuu gccuuu 1.925 2.71e-02

2919 acguuu ccuuuu 1.925 2.71e-02

2929 uuguuu uuguuu 2.558 5.26e-03

2958 uuguuu uaguuu 2.654 3.98e-03

2959 aguuuu aguuuu 3.556 1.88e-04

2960 uuguuu guuuuu 3.240 5.98e-04

2961 uuguuu uuuuug 2.913 1.79e-03

2964 uuguuu uugguu 2.885 1.96e-03

2965 uuguuu ugguuu 2.885 1.96e-03

2966 aguuuu gguuug 3.321 4.48e-04

2972 acguuu caguuu 2.425 7.65e-03

2973 aguuuu aguuuu 3.802 7.18e-05

2974 uuguuu guuuuu 3.240 5.98e-04

2975 uuguuu uuuuug 2.913 1.79e-03

2978 uuguuu uuguug 3.106 9.48e-04

2979 aguuuu uguugu 3.309 4.68e-04

2981 uuguuu uuguug 3.106 9.48e-04

2982 aguuuu uguugu 3.309 4.68e-04

Protein: EIF4G2(Hs/Mm)

Sequence Position Motif K-mer Z-score P-value

608 gcgag gccag 1.813 3.49e-02

614 gcgag gggag 1.813 3.49e-02

618 gcgag gccag 1.813 3.49e-02

764 gguugc uguugu 2.130 1.66e-02

988 cgccg cgcca 2.067 1.94e-02

995 cgccg agccg 2.067 1.94e-02

998 cgccg cgcag 2.067 1.94e-02

1002 gcgag gcgug 1.813 3.49e-02

1591 gcgag gcgag 1.813 3.49e-02

2223 gguugc ggaugc 2.130 1.66e-02

2967 gguugc guuugc 2.221 1.32e-02

2979 gguugc uguugu 2.221 1.32e-02

2982 gguugc uguugu 2.221 1.32e-02

Protein: ELAVL4(Hs/Mm)

Sequence Position Motif K-mer Z-score P-value

2929 uuauu uuguu 2.248 1.23e-02

2947 uuauu guauu 2.422 7.72e-03

2961 uuauu uuuuu 3.183 7.29e-04

2975 uuauu uuuuu 3.183 7.29e-04

2978 uuauu uuguu 2.982 1.43e-03

2981 uuauu uuguu 2.982 1.43e-03

Protein: ENOX1(Hs/Mm)

Sequence Position Motif K-mer Z-score P-value

927 hrkacag ccuacag 2.438 7.38e-03

1191 hrkacag cagacug 2.172 1.49e-02

1231 hrkacag aggacag 2.781 2.71e-03

1252 hrkacag aagccag 2.891 1.92e-03

1271 hrkacag aagacag 3.234 6.10e-04

1992 hrkacag cagccag 2.375 8.77e-03

2009 hrkacag ccaacag 2.234 1.27e-02

2014 hrkacag aguacag 2.375 8.77e-03

Protein: ESRP1(Hs/Mm)

Sequence Position Motif K-mer Z-score P-value

1025 gggugg uggugg 2.193 1.42e-02

2201 gggugg gggugc 1.909 2.81e-02

2216 gggugg uggagg 1.875 3.04e-02

2219 gggugg aggagg 1.875 3.04e-02

2279 gggugg uggagg 1.784 3.72e-02

2282 gggugg aggagg 1.784 3.72e-02

Protein: ESRP2(Hs/Mm)

Sequence Position Motif K-mer Z-score P-value

105 ugggrad uggggaa 2.737 3.10e-03

297 ugggrad gggggau 1.697 4.48e-02

368 ugggrad uguggag 1.855 3.18e-02

1844 ugggrad uggggaa 2.658 3.93e-03

2651 ugggrad aggggau 2.303 1.06e-02

2681 ugggrad uggggag 3.724 9.80e-05

2702 ugggrad ugggguu 3.342 4.16e-04

Protein: EWSR1(Hs/Mm)

Sequence Position Motif K-mer Z-score P-value

80 aggug agggg 1.991 2.32e-02

159 aggug agcug 1.991 2.32e-02

162 aggug uggug 1.991 2.32e-02

174 aggug aguug 1.991 2.32e-02

177 aggug uggug 1.991 2.32e-02

2756 gggggggg gggagggu 2.577 4.98e-03

Protein: FMR1(Hs/Mm)

Sequence Position Motif K-mer Z-score P-value

50 kgacarg ugacgag 2.403 8.13e-03

72 kgacarg ggccaag 2.417 7.82e-03

Protein: FUBP1(Hs/Mm)

Sequence Position Motif K-mer Z-score P-value

831 uauguau acugugu 2.613 4.49e-03

833 uauguau ugugucu 2.813 2.45e-03

846 uauguau cauggau 2.373 8.82e-03

1732 uauguau caugucu 2.080 1.88e-02

1734 uauguau ugucuau 1.880 3.01e-02

2928 uauguau cuuguuu 2.173 1.49e-02

2930 uauuu uguuu 1.952 2.55e-02

2944 uauguau ucaguau 2.173 1.49e-02

2948 uauuu uauug 2.368 8.94e-03

2949 uauguau auuguaa 1.707 4.39e-02

2961 uauuu uuuuu 3.120 9.04e-04

2975 uauuu uuuuu 3.104 9.55e-04

2983 uauguau guuguuu 1.667 4.78e-02

Protein: FUBP3(Hs/Mm)

Sequence Position Motif K-mer Z-score P-value

2961 uuuau uuuuu 2.909 1.81e-03

2975 uuuau uuuuu 3.157 7.97e-04

2977 uuuau uuugu 2.868 2.07e-03

Protein: FUS(Hs/Mm)

Sequence Position Motif K-mer Z-score P-value

533 cgcgc ugcgc 2.120 1.70e-02

535 cgcgc cgcgc 1.952 2.55e-02

986 cgcgc ugcgc 1.892 2.92e-02

Protein: FXR1(Hs/Mm)

Sequence Position Motif K-mer Z-score P-value

1708 aygacr caggac 1.943 2.60e-02

1755 aygacr aauggc 2.786 2.67e-03

2773 aygacr caugac 1.700 4.46e-02

Protein: FXR2(Hs/Mm)

Sequence Position Motif K-mer Z-score P-value

723 dgacrrr ggacggg 1.929 2.69e-02

1174 dgacrrr gaacggg 2.143 1.61e-02

1192 dgacrrr agacugg 2.143 1.61e-02

Protein: G3BP2(Hs/Mm)

Sequence Position Motif K-mer Z-score P-value

1268 aggaudr aggaaga 2.147 1.59e-02

1615 aggaudr aggaugg 2.893 1.91e-03

1625 aggaudr agaaugg 2.427 7.61e-03

2219 aggaudr aggagga 2.613 4.49e-03

2222 aggaudr aggaugc 2.787 2.66e-03

2282 aggaudr aggagga 2.907 1.82e-03

2285 aggaudr aggauga 3.360 3.90e-04

Protein: HNRNPA0(Hs/Mm)

Sequence Position Motif K-mer Z-score P-value

2937 uauag uauaa 1.820 3.44e-02

2948 uauag uauug 1.820 3.44e-02

2956 uauag acuag 1.865 3.11e-02

Protein: HNRNPA1(Hs/Mm)

Sequence Position Motif K-mer Z-score P-value

1232 rgnyag ggacag 2.291 1.10e-02

1253 rgnyag agccag 2.982 1.43e-03

1268 rgnyag aggaag 2.200 1.39e-02

1272 rgnyag agacag 2.382 8.61e-03

2451 rgnyag gggcag 2.091 1.83e-02

2680 duagggw cugggga 2.419 7.78e-03

2691 duagggw cuagggc 3.135 8.59e-04

2954 guaguagu aaacuagu 2.689 3.58e-03

2968 guaguagu uuugcagu 3.164 7.78e-04

2980 guaguagu guuguugu 3.279 5.21e-04

Protein: HNRNPA1L2(Hs/Mm)

Sequence Position Motif K-mer Z-score P-value

2680 duagggw cugggga 2.296 1.08e-02

2691 duagggw cuagggc 3.070 1.07e-03

2701 duagggw cuggggu 2.718 3.28e-03

Protein: HNRNPA2B1(Hs/Mm)

Sequence Position Motif K-mer Z-score P-value

80 ggggg agggg 2.256 1.20e-02

105 ggggg ugggg 2.109 1.75e-02

296 ggggg ugggg 1.853 3.19e-02

297 ggggg ggggg 2.093 1.82e-02

396 ggggg cgggg 2.202 1.38e-02

397 ggggg ggggg 2.442 7.30e-03

673 ggggg cgggg 2.109 1.75e-02

693 ggggg agggg 2.256 1.20e-02

788 gggua gggug 1.800 3.59e-02

1615 aggwuhgr aggaugga 1.873 3.05e-02

1823 gggua gggug 2.276 1.14e-02

1844 ggggg ugggg 1.814 3.48e-02

1845 gggua gggga 1.800 3.59e-02

1846 gggua gggaa 1.800 3.59e-02

2048 ggggg agggg 1.752 3.99e-02

2372 ggggg agggg 2.140 1.62e-02

2373 ggggg ggggg 2.240 1.25e-02

2451 gggua gggca 1.800 3.59e-02

2651 ggggg agggg 1.752 3.99e-02

2660 gggua ggguc 1.800 3.59e-02

2680 duagggw cugggga 2.360 9.14e-03

2681 ggggg ugggg 2.225 1.30e-02

2682 gggua gggga 1.800 3.59e-02

2691 duagggw cuagggc 2.547 5.43e-03

2702 ggggg ugggg 2.132 1.65e-02

2754 ggggg agggg 2.372 8.85e-03

Protein: HNRNPC(Hs/Mm)

Sequence Position Motif K-mer Z-score P-value

2920 huuuuuk cuuuugu 2.152 1.57e-02

2921 uuuuu uuuug 1.945 2.59e-02

2922 uuuuu uuugu 1.945 2.59e-02

2928 huuuuuk cuuguuu 2.152 1.57e-02

2929 uuuuu uuguu 1.945 2.59e-02

2930 uuuuu uguuu 1.945 2.59e-02

2959 huuuuuk aguuuuu 2.973 1.47e-03

2960 huuuuuk guuuuug 3.089 1.00e-03

2961 uuuuu uuuuu 3.075 1.05e-03

2962 uuuuu uuuug 2.534 5.64e-03

2973 huuuuuk aguuuuu 2.973 1.47e-03

2974 huuuuuk guuuuug 3.089 1.00e-03

2975 uuuuu uuuuu 3.075 1.05e-03

2976 huuuuuk uuuuguu 2.741 3.06e-03

2977 uuuuu uuugu 2.534 5.64e-03

2978 uuuuu uuguu 2.534 5.64e-03

2981 uuuuu uuguu 2.534 5.64e-03

2983 huuuuuk guuguuu 2.679 3.69e-03

Protein: HNRNPCL1(Hs/Mm)

Sequence Position Motif K-mer Z-score P-value

2920 huuuuuk cuuuugu 2.149 1.58e-02

2921 uuuuu uuuug 2.143 1.61e-02

2922 uuuuu uuugu 2.098 1.80e-02

2928 huuuuuk cuuguuu 2.149 1.58e-02

2929 uuuuu uuguu 2.098 1.80e-02

2930 uuuuu uguuu 2.098 1.80e-02

2959 huuuuuk aguuuuu 2.939 1.65e-03

2960 huuuuuk guuuuug 3.035 1.20e-03

2961 uuuuu uuuuu 3.263 5.51e-04

2962 uuuuu uuuug 2.827 2.35e-03

2973 huuuuuk aguuuuu 2.939 1.65e-03

2974 huuuuuk guuuuug 3.035 1.20e-03

2975 uuuuu uuuuu 3.263 5.51e-04

2976 uuuuu uuuug 2.827 2.35e-03

2977 uuuuu uuugu 2.737 3.10e-03

2978 uuuuu uuguu 2.737 3.10e-03

2981 uuuuu uuguu 2.737 3.10e-03

2983 huuuuuk guuguuu 2.667 3.83e-03

Protein: HNRNPDL(Hs/Mm)

Sequence Position Motif K-mer Z-score P-value

2779 uaaaa uaaaa 1.653 4.92e-02

2939 uaauu uaacu 1.676 4.69e-02

Protein: HNRNPF(Hs/Mm)

Sequence Position Motif K-mer Z-score P-value

80 agggu agggg 1.980 2.39e-02

86 agggu ugggc 2.367 8.97e-03

105 agggu ugggg 2.367 8.97e-03

272 gugkau gugucu 1.768 3.85e-02

787 agggu ugggu 2.898 1.88e-03

809 agggu ugggg 2.143 1.61e-02

847 gugkau auggau 2.232 1.28e-02

853 gukgykg guuguga 1.918 2.76e-02

1519 agggu ugggu 1.704 4.42e-02

1528 agggu uggga 2.469 6.77e-03

1553 agggu agggc 2.551 5.37e-03

1570 agggu ugggg 1.949 2.56e-02

1577 agggu ugggc 1.949 2.56e-02

1614 gugkau gaggau 2.161 1.53e-02

1822 agggu ugggu 2.898 1.88e-03

1841 gggagggg agcugggg 1.758 3.94e-02

1844 agggu ugggg 2.143 1.61e-02

1931 gugkau guggcu 2.036 2.09e-02

2048 agggu agggg 2.010 2.22e-02

2049 agggu ggggu 2.010 2.22e-02

2072 agggu ugggc 1.969 2.45e-02

2221 gugkau gaggau 2.089 1.84e-02

2284 gugkau gaggau 2.179 1.47e-02

2450 agggu ugggc 2.408 8.02e-03

2469 agggu agggc 2.000 2.27e-02

2475 agggu agggc 2.000 2.27e-02

2651 agggu agggg 2.541 5.53e-03

2659 agggu cgggu 2.898 1.88e-03

2667 agggu uggga 2.990 1.39e-03

2681 agggu ugggg 2.918 1.76e-03

2693 agggu agggc 2.786 2.67e-03

2702 agggu ugggg 2.755 2.93e-03

2703 agggu ggggu 2.796 2.59e-03

2716 agggu ugggc 2.418 7.80e-03

2751 gggagggg aagagggg 2.076 1.89e-02

2754 agggu agggg 2.214 1.34e-02

2756 gggagggg gggagggu 2.091 1.83e-02

2759 agggu agggu 2.490 6.39e-03

2977 gukgykg uuuguug 3.311 4.65e-04

2980 gukgykg guuguug 3.689 1.13e-04

2983 gukgykg guuguuu 3.262 5.53e-04

Protein: HNRNPH1(Hs/Mm)

Sequence Position Motif K-mer Z-score P-value

51 gargag gacgag 3.150 8.16e-04

56 gargag gaagag 3.460 2.70e-04

382 gargag gcagag 1.910 2.81e-02

657 gargag gaagag 2.100 1.79e-02

666 gargag caagag 1.700 4.46e-02

1621 gargag gaacag 2.160 1.54e-02

1631 gargag ggagag 2.750 2.98e-03

2281 gargag gaggag 3.020 1.26e-03

2290 gargag gaaaag 2.840 2.26e-03

2750 gargag gaagag 3.210 6.64e-04

Protein: HNRNPH2(Hs/Mm)

Sequence Position Motif K-mer Z-score P-value

68 gggaggg aggaggc 2.178 1.47e-02

295 gggggg cugggg 2.220 1.32e-02

296 gggggg uggggg 2.319 1.02e-02

396 gggggg cggggg 2.187 1.44e-02

400 gggaggg ggcaggc 1.733 4.15e-02

1612 gggaggg uggagga 1.967 2.46e-02

1630 gggaggg gggagag 2.089 1.84e-02

2371 gggggg cagggg 2.143 1.61e-02

2372 gggggg aggggg 2.275 1.15e-02

2668 gggaggg gggaugg 2.667 3.83e-03

2672 gggaggg uggaggc 2.456 7.02e-03

2680 gggggg cugggg 2.099 1.79e-02

2701 gggggg cugggg 2.099 1.79e-02

2753 gggggg gagggg 2.495 6.30e-03

2756 gggaggg gggaggg 2.678 3.70e-03

Protein: HNRNPK(Hs/Mm)

Sequence Position Motif K-mer Z-score P-value

63 gccca gacca 2.109 1.75e-02

72 gccca ggcca 2.109 1.75e-02

73 gccca gccaa 2.336 9.75e-03

88 gccca ggcca 2.336 9.75e-03

98 gccca gccaa 1.655 4.90e-02

264 gccca gcaca 2.109 1.75e-02

280 gccca gcccc 2.336 9.75e-03

282 gccca cccca 2.336 9.75e-03

289 gccca gucca 2.336 9.75e-03

303 gccca uccca 2.109 1.75e-02

427 gccca accca 1.655 4.90e-02

444 cacgc caccc 2.433 7.49e-03

447 ccawmcc ccugccc 1.739 4.10e-02

450 gccca gcccc 1.655 4.90e-02

451 gccca cccca 1.655 4.90e-02

463 cacgc cacac 2.433 7.49e-03

465 cacgc cacuc 2.433 7.49e-03

469 cacgc caggc 2.433 7.49e-03

536 gccca gcgca 1.655 4.90e-02

550 ccawmcc ccagcca 2.754 2.94e-03

554 ccawmcc ccauacc 3.087 1.01e-03

824 gccca cccca 1.655 4.90e-02

843 gccca uccca 1.655 4.90e-02

867 gccca gcccc 2.336 9.75e-03

870 gccca cccca 2.109 1.75e-02

882 gccca gacca 2.455 7.04e-03

891 gccca cccca 2.509 6.05e-03

893 ccawmcc ccagccc 3.362 3.87e-04

896 gccca gcccu 2.336 9.75e-03

907 gccca gcccc 2.455 7.04e-03

909 ccawmcc cccuccc 2.783 2.69e-03

913 gccca cccca 2.336 9.75e-03

926 gccca gccua 1.655 4.90e-02

1078 ccawmcc ccagcca 1.841 3.28e-02

1083 cacgc cacgg 1.952 2.55e-02

1092 cacgc caccc 1.952 2.55e-02

1103 cacgc uacgc 1.952 2.55e-02

1129 gccca ggcca 2.109 1.75e-02

1148 gccca gcccc 1.655 4.90e-02

1149 gccca cccca 1.655 4.90e-02

1318 gccca ggcca 1.655 4.90e-02

1329 gccca ggcca 1.655 4.90e-02

1331 ccawmcc ccauccu 2.072 1.91e-02

1335 ccawmcc ccugccc 1.928 2.69e-02

1338 gccca gcccc 2.109 1.75e-02

1390 gccca gccca 2.564 5.17e-03

1402 gccca gcccu 2.109 1.75e-02

1411 gccca gccua 2.109 1.75e-02

1469 gccca gccca 1.655 4.90e-02

1471 ccawmcc ccagcuc 2.043 2.05e-02

1479 gccca gacca 2.109 1.75e-02

1488 ccawmcc ccaugcc 2.768 2.82e-03

1586 ccawmcc ccagcgc 1.725 4.23e-02

1741 gccca gccua 1.655 4.90e-02

1762 ccawmcc cccuccc 2.594 4.74e-03

1765 gccca uccca 2.109 1.75e-02

1767 ccawmcc ccaugcc 2.304 1.06e-02

1771 gccca gcccc 2.109 1.75e-02

1772 gccca cccca 2.109 1.75e-02

1791 gccca gcaca 2.109 1.75e-02

1810 gccca gcaca 1.655 4.90e-02

1833 gccca gcccc 1.655 4.90e-02

1853 gccca gcccc 1.655 4.90e-02

1857 ccawmcc ccuuacc 1.681 4.64e-02

1861 gccca accca 1.655 4.90e-02

1881 gccca gacca 1.655 4.90e-02

1883 ccawmcc ccaacgc 2.971 1.48e-03

1896 ccawmcc ccagucc 2.551 5.37e-03

1991 ccawmcc ccagcca 1.841 3.28e-02

2135 gccca gcccc 2.109 1.75e-02

2136 gccca cccca 2.109 1.75e-02

2159 gccca ggcca 2.509 6.05e-03

2160 gccca gccaa 2.509 6.05e-03

2168 gccca uccca 2.336 9.75e-03

2174 gccca accca 2.336 9.75e-03

2179 gccca gcuca 2.336 9.75e-03

2250 cacgc cacgc 2.913 1.79e-03

2254 ccawmcc ccaccca 1.971 2.44e-02

2255 cacgc caccc 2.433 7.49e-03

2256 gccca accca 1.655 4.90e-02

2258 ccawmcc ccaucac 2.087 1.84e-02

2264 ccawmcc ccaagcu 2.058 1.98e-02

2265 cacgc caagc 2.433 7.49e-03

2304 gccca gcccu 1.655 4.90e-02

2314 gccca uccca 1.655 4.90e-02

2324 gccca gcuca 2.109 1.75e-02

2343 gccca cccca 1.655 4.90e-02

2353 gccca gucca 1.655 4.90e-02

2378 gccca accca 2.336 9.75e-03

2393 ccawmcc ccaccca 3.275 5.28e-04

2395 gccca accca 2.336 9.75e-03

2397 ccawmcc ccagccc 3.870 5.44e-05

2400 gccca gcccc 2.336 9.75e-03

2401 gccca cccca 2.336 9.75e-03

2403 ccawmcc ccaaucc 3.942 4.04e-05

2404 ccawmcc caauccc 3.507 2.27e-04

2407 gccca uccca 2.109 1.75e-02

2456 gccca gcccc 2.564 5.17e-03

2457 gccca cccca 2.564 5.17e-03

2461 ccawmcc acaagcc 1.884 2.98e-02

2462 ccawmcc caagccc 1.913 2.79e-02

2465 gccca gccca 3.018 1.27e-03

2471 gccca ggcca 2.564 5.17e-03

2526 gccca cccca 2.109 1.75e-02

2528 ccawmcc ccagccc 3.072 1.06e-03

2531 gccca gcccc 1.655 4.90e-02

2532 gccca cccca 1.655 4.90e-02

2534 ccawmcc ccaaguc 3.058 1.11e-03

2559 ccawmcc ccaggcc 2.507 6.09e-03

2564 ccawmcc ccagccc 2.725 3.22e-03

2567 gccca gcccc 1.655 4.90e-02

2588 gccca cccca 2.109 1.75e-02

2590 ccawmcc ccagccc 2.986 1.41e-03

2593 gccca gcccc 1.655 4.90e-02

2594 gccca cccca 1.655 4.90e-02

2596 ccawmcc ccaagcc 3.000 1.35e-03

2770 gccca gccca 3.473 2.57e-04

2785 ccawmcc cuacccc 2.754 2.94e-03

2788 gccca cccca 2.336 9.75e-03

2790 ccawmcc ccagccc 3.188 7.16e-04

2793 gccca gccca 3.018 1.27e-03

2806 ccawmcc ccaucuc 2.899 1.87e-03

2859 gccca uccca 1.655 4.90e-02

2884 gccca gacca 2.109 1.75e-02

2898 ccawmcc ccaucca 3.348 4.07e-04

2902 ccawmcc ccagccc 3.623 1.46e-04

2905 gccca gcccc 2.109 1.75e-02

2906 gccca cccca 2.109 1.75e-02

2907 ccawmcc cccaccc 3.435 2.96e-04

2908 ccawmcc ccaccca 3.101 9.64e-04

2910 gccca accca 1.655 4.90e-02

Protein: HNRNPL(Hs/Mm)

Sequence Position Motif K-mer Z-score P-value

152 uacaca aucaca 1.789 3.68e-02

153 acacrav ucacaaa 2.155 1.56e-02

154 uacaca cacaaa 1.908 2.82e-02

168 acacrav acaucaa 2.028 2.13e-02

461 uacaca accaca 1.789 3.68e-02

462 amayama ccacacu 2.160 1.54e-02

463 uacaca cacacu 1.895 2.90e-02

464 amayama acacuca 2.387 8.49e-03

750 amayama acgcaaa 2.467 6.81e-03

752 amayama gcaaaca 2.573 5.04e-03

753 uacaca caaaca 1.816 3.47e-02

754 amayama aaacacc 2.680 3.68e-03

755 uacaca aacacc 1.855 3.18e-02

964 uacaca cacaua 2.461 6.93e-03

1348 uacaca aacacc 1.974 2.42e-02

1351 uacaca accaca 1.882 2.99e-02

1353 uacaca cacaga 2.039 2.07e-02

1790 uacaca agcaca 1.684 4.61e-02

1809 uacaca ggcaca 1.684 4.61e-02

2060 uacaca uacaca 1.829 3.37e-02

2061 acacrav acacauc 2.169 1.50e-02

Protein: HNRNPM(Hs/Mm)

Sequence Position Motif K-mer Z-score P-value

1299 gguugguu gguuucuu 2.841 2.25e-03

2962 gguugguu uuuugguu 3.079 1.04e-03

2982 gguugguu uguuguuu 3.333 4.30e-04

Protein: HNRNPU(Hs/Mm)

Sequence Position Motif K-mer Z-score P-value

835 uguauug ugucuug 1.797 3.62e-02

2965 uguauug ugguuug 2.734 3.13e-03

Protein: HNRPLL(Hs/Mm)

Sequence Position Motif K-mer Z-score P-value

338 rcahaca gcagcca 1.873 3.05e-02

462 rcahaca ccacacu 2.190 1.43e-02

464 rcahaca acacuca 2.557 5.28e-03

585 rcahaca ccucaca 2.013 2.21e-02

589 rcahaca acaacca 2.215 1.34e-02

748 rcahaca ccacgca 1.646 4.99e-02

752 rcahaca gcaaaca 1.823 3.42e-02

792 rcahaca gcaggca 1.848 3.23e-02

951 rcahaca gcagcca 2.456 7.02e-03

963 rcahaca gcacaua 2.139 1.62e-02

965 rcahaca acauaaa 2.177 1.47e-02

Protein: HuR(Hs/Mm)

Sequence Position Motif K-mer Z-score P-value

2957 uukruuu cuaguuu 3.231 6.17e-04

2958 uukruuu uaguuuu 3.044 1.17e-03

2963 uukruuu uuugguu 3.330 4.34e-04

2964 uukruuu uugguuu 3.780 7.84e-05

2977 uukruuu uuuguug 3.275 5.28e-04

2978 uukruuu uuguugu 3.055 1.13e-03

2981 uukruuu uuguugu 3.055 1.13e-03

2983 uukruuu guuguuu 3.374 3.70e-04

Protein: IGF2BP2(Hs/Mm)

Sequence Position Motif K-mer Z-score P-value

754 caacaca aaacacc 2.014 2.20e-02

1347 caacaca caacacc 2.571 5.07e-03

1350 caacaca caccaca 2.414 7.89e-03

1352 caacaca ccacaga 2.414 7.89e-03

1621 caacaca gaacaga 1.971 2.44e-02

Protein: ILF2(Hs/Mm)

Sequence Position Motif K-mer Z-score P-value

788 gggua gggug 1.800 3.59e-02

1823 gggua gggug 2.276 1.14e-02

1845 gggua gggga 1.800 3.59e-02

1846 gggua gggaa 1.800 3.59e-02

2451 gggua gggca 1.800 3.59e-02

2660 gggua ggguc 1.800 3.59e-02

2682 gggua gggga 1.800 3.59e-02

2756 gggugggg gggagggu 2.078 1.89e-02

Protein: KHDRBS2(Hs/Mm)

Sequence Position Motif K-mer Z-score P-value

2777 rauaaam acuaaaa 2.329 9.93e-03

Protein: KHDRBS3(Hs/Mm)

Sequence Position Motif K-mer Z-score P-value

2765 aauaaa cauaag 1.856 3.17e-02

2777 aauaaa acuaaa 2.067 1.94e-02

2779 auaaav uaaaaa 1.717 4.30e-02

Protein: KHSRP(Hs/Mm)

Sequence Position Motif K-mer Z-score P-value

833 uguau ugugu 1.748 4.02e-02

835 uguau ugucu 1.748 4.02e-02

2614 uguau ugugu 1.738 4.11e-02

2616 uguau ugucu 1.738 4.11e-02

2929 uuuuu uuguu 2.023 2.15e-02

2930 uguau uguuu 1.907 2.83e-02

2961 uuuuu uuuuu 3.053 1.13e-03

2975 uuuuu uuuuu 3.053 1.13e-03

2978 uuuuu uuguu 2.748 3.00e-03

2981 uuuuu uuguu 2.748 3.00e-03

Protein: LIN28A(Hs/Mm)

Sequence Position Motif K-mer Z-score P-value

52 hggagwa acgagaa 2.486 6.46e-03

131 hggagwa aggugaa 1.959 2.51e-02

370 hggagwa uggagac 2.824 2.37e-03

378 hggagwa gggagca 2.784 2.68e-03

1162 hggagwa cugagaa 2.041 2.06e-02

1630 hggagwa gggagag 2.878 2.00e-03

1655 hggagwa aggagaa 2.149 1.58e-02

2746 hggagwa uggagaa 3.149 8.19e-04

Protein: MATR3(Hs/Mm)

Sequence Position Motif K-mer Z-score P-value

835 maucuur ugucuug 2.095 1.81e-02

850 maucuur gauguug 2.054 2.00e-02

Protein: MBNL1(Hs/Mm)

Sequence Position Motif K-mer Z-score P-value

28 gcuugc agcugg 2.558 5.26e-03

140 gcuugc aacuug 2.636 4.19e-03

180 cgcuu ugcua 1.975 2.41e-02

205 gcuugc agccug 2.013 2.21e-02

210 gcuugc gacuug 2.013 2.21e-02

215 gcgcagc gggcugc 2.194 1.41e-02

233 cgcuu ugcug 1.925 2.71e-02

336 gcgcagc cugcagc 2.736 3.11e-03

380 gcgcagc gagcaga 2.319 1.02e-02

399 gcgcagc gggcagg 2.931 1.69e-03

403 gcgcagc aggcagc 3.194 7.02e-04

406 gcgcagc cagcagc 2.681 3.67e-03

498 gcgcagc gggcaac 1.889 2.94e-02

505 gcuugc agccug 2.091 1.83e-02

529 gcgcagc gagcugc 2.181 1.46e-02

727 gcgcagc gggcaga 1.889 2.94e-02

780 gcgcagc gggcauc 2.056 1.99e-02

790 gcgcagc gugcagg 2.542 5.51e-03

817 gcuugc agcaug 2.000 2.27e-02

932 cgcuu agcug 1.987 2.35e-02

937 cgcuu ugcug 2.012 2.21e-02

982 gcgcagc gagcugc 3.181 7.34e-04

987 gcgcagc gcgccac 3.111 9.32e-04

994 gcgcagc aagccgc 3.153 8.08e-04

997 gcgcagc ccgcagc 3.528 2.09e-04

1015 gcuugc agcugg 2.091 1.83e-02

1104 gcgcagc acgcugc 1.861 3.14e-02

1105 cgcuu cgcug 1.725 4.23e-02

1114 cgcuu cgcug 1.725 4.23e-02

1216 gcgcagc aggcagc 3.236 6.06e-04

1237 gcgcagc gugcagc 1.944 2.59e-02

1359 gcgcagc gcucagc 2.375 8.77e-03

1826 gcuugc ugccug 2.000 2.27e-02

2184 gcgcagc cagcagc 2.264 1.18e-02

2440 gcgcagc gggcagc 3.556 1.88e-04

2451 gcgcagc gggcagc 3.069 1.07e-03

2678 cgcuu cgcug 2.750 2.98e-03

2686 cgcuu agcuu 2.700 3.47e-03

2695 gcuugc ggcuuc 3.000 1.35e-03

2712 gcuugc uucuug 2.636 4.19e-03

2739 gcuugc agcuag 2.519 5.88e-03

2743 gcuugc agcugg 2.519 5.88e-03

Protein: MSI1(Hs/Mm)

Sequence Position Motif K-mer Z-score P-value

1599 uagca uagca 2.567 5.13e-03

Protein: NOVA1(Hs/Mm)

Sequence Position Motif K-mer Z-score P-value

152 aucac aucac 1.781 3.75e-02

302 aucac auccc 2.733 3.14e-03

304 uucauaa cccauca 2.767 2.83e-03

307 aucac aucac 3.210 6.64e-04

319 uucauaa ugcauca 2.767 2.83e-03

322 uucauaa aucauca 2.983 1.43e-03

325 aucac aucaa 2.733 3.14e-03

439 aucac aucau 2.257 1.20e-02

442 aucac aucac 2.733 3.14e-03

461 aucac accac 2.257 1.20e-02

577 uucauaa cucauga 3.000 1.35e-03

601 uucauaa cucaucu 2.383 8.59e-03

1781 uucauaa ugcaucu 1.667 4.78e-02

1981 uucauaa cucaugg 1.833 3.34e-02

2260 aucac aucac 1.781 3.75e-02

2802 aucac cucac 2.257 1.20e-02

2808 aucac aucuc 2.257 1.20e-02

2815 aucac gucac 2.257 1.20e-02

2824 aucac aucuc 2.495 6.30e-03

2890 uucauaa aucaugc 2.233 1.28e-02

Protein: NUPL2(Hs/Mm)

Sequence Position Motif K-mer Z-score P-value

690 caaagg ccaagg 1.830 3.36e-02

691 caaagg caaggg 1.830 3.36e-02

2781 aaaauaa aaaacua 1.701 4.45e-02

Protein: PABPC1(Hs/Mm)

Sequence Position Motif K-mer Z-score P-value

2291 araaaam aaaagaa 2.049 2.02e-02

2778 araaaam cuaaaaa 2.078 1.89e-02

Protein: PABPC4(Hs/Mm)

Sequence Position Motif K-mer Z-score P-value

2290 aaaaaar gaaaaga 1.927 2.70e-02

2291 aaaaaar aaaagaa 2.018 2.18e-02

2292 aaaaaar aaagaag 1.827 3.38e-02

2778 aaaaaar cuaaaaa 2.236 1.27e-02

2779 aaaaaar uaaaaac 2.291 1.10e-02

2780 aaaaaar aaaaacu 2.318 1.02e-02

Protein: PABPN1(Hs/Mm)

Sequence Position Motif K-mer Z-score P-value

55 araaga agaaga 1.750 4.01e-02

2291 araaga aaaaga 2.324 1.01e-02

2294 araaga agaagc 2.519 5.88e-03

Protein: PABPN1L(Hs/Mm)

Sequence Position Motif K-mer Z-score P-value

2291 aaaaa aaaag 2.036 2.09e-02

2780 aaaaa aaaaa 2.159 1.54e-02

2781 aaaaa aaaac 1.942 2.61e-02

Protein: PCBP1(Hs/Mm)

Sequence Position Motif K-mer Z-score P-value

279 agcccc agcccc 2.400 8.20e-03

280 agcccc gccccc 2.089 1.84e-02

446 ccwwhcc cccugcc 2.636 4.19e-03

447 ccwwhcc ccugccc 2.662 3.88e-03

451 ccwwhcc ccccacc 2.753 2.95e-03

473 ccwwhcc ccuuucu 1.935 2.65e-02

554 ccwwhcc ccauacc 3.234 6.10e-04

567 ccwwhcc ccucucc 3.117 9.14e-04

866 agcccc agcccc 3.189 7.14e-04

867 agcccc gccccc 2.822 2.39e-03

868 agcccc cccccc 2.722 3.24e-03

889 agcccc gacccc 3.244 5.89e-04

892 ccwwhcc cccagcc 2.818 2.42e-03

893 ccwwhcc ccagccc 2.818 2.42e-03

895 agcccc agcccu 2.689 3.58e-03

906 agcccc ggcccc 2.867 2.07e-03

908 ccwwhcc ccccucc 2.844 2.23e-03

909 ccwwhcc cccuccc 3.078 1.04e-03

910 ccwwhcc ccucccc 3.026 1.24e-03

911 agcccc cucccc 2.756 2.93e-03

1030 agcccc ggcccc 3.011 1.30e-03

1031 agcccc gccccc 2.733 3.14e-03

1033 ccwwhcc ccccucc 2.130 1.66e-02

1034 ccwwhcc cccuccc 2.364 9.04e-03

1035 ccwwhcc ccucccc 2.312 1.04e-02

1036 agcccc cucccc 2.900 1.87e-03

1047 agcccc ggcccu 2.700 3.47e-03

1126 ccwwhcc ccuggcc 2.052 2.01e-02

1532 agcccc agcccc 2.900 1.87e-03

1533 agcccc gccccc 2.567 5.13e-03

1534 agcccc cccccc 2.644 4.10e-03

1579 agcccc ggcccc 2.789 2.64e-03

1580 agcccc gccccc 2.522 5.83e-03

1581 agcccc cccccc 2.622 4.37e-03

1582 agcccc cccccc 2.622 4.37e-03

1762 ccwwhcc cccuccc 2.338 9.69e-03

1767 ccwwhcc ccaugcc 2.338 9.69e-03

1832 agcccc agcccc 3.189 7.14e-04

1833 agcccc gccccc 2.722 3.24e-03

1852 agcccc agcccc 3.389 3.51e-04

1853 agcccc gccccc 2.933 1.68e-03

1857 ccwwhcc ccuuacc 1.792 3.66e-02

2134 agcccc agcccc 1.711 4.35e-02

2150 agcccc aucccc 2.333 9.82e-03

2228 ccwwhcc ccuucca 1.662 4.83e-02

2340 ccwwhcc ccucccc 2.169 1.50e-02

2396 ccwwhcc cccagcc 2.571 5.07e-03

2397 ccwwhcc ccagccc 2.558 5.26e-03

2399 agcccc agcccc 2.311 1.04e-02

2403 ccwwhcc ccaaucc 2.831 2.32e-03

2455 agcccc agcccc 1.667 4.78e-02

2504 agcccc agcccc 2.611 4.51e-03

2520 ccwwhcc ccucacc 2.299 1.08e-02

2523 agcccc cacccc 3.000 1.35e-03

2524 agcccc accccc 2.922 1.74e-03

2527 ccwwhcc cccagcc 2.065 1.95e-02

2528 ccwwhcc ccagccc 2.065 1.95e-02

2530 agcccc agcccc 2.611 4.51e-03

2564 ccwwhcc ccagccc 1.805 3.55e-02

2566 agcccc agcccc 1.733 4.15e-02

2586 agcccc aacccc 2.600 4.66e-03

2589 ccwwhcc cccagcc 2.403 8.13e-03

2590 ccwwhcc ccagccc 1.922 2.73e-02

2592 agcccc agcccc 1.733 4.15e-02

2596 ccwwhcc ccaagcc 2.013 2.21e-02

2769 agcccc agccca 2.178 1.47e-02

2786 agcccc uacccc 2.222 1.31e-02

2789 ccwwhcc cccagcc 2.494 6.32e-03

2790 ccwwhcc ccagccc 2.481 6.55e-03

2792 agcccc agccca 2.178 1.47e-02

2829 ccwwhcc ccucucc 2.143 1.61e-02

2902 ccwwhcc ccagccc 1.935 2.65e-02

2906 ccwwhcc ccccacc 1.935 2.65e-02

2907 ccwwhcc cccaccc 2.013 2.21e-02

Protein: PCBP2(Hs/Mm)

Sequence Position Motif K-mer Z-score P-value

219 uuccc ugccc 1.743 4.07e-02

238 uuccc cuccc 1.734 4.15e-02

239 uuccc ucccc 1.743 4.07e-02

444 aaccc caccc 1.752 3.99e-02

446 ccyycch cccugcc 2.363 9.06e-03

447 ccyycch ccugccc 2.587 4.84e-03

451 ccccacccc ccccaccuc 2.393 8.36e-03

544 aaccc uaccc 2.211 1.35e-02

546 ccyycch cccucca 2.662 3.88e-03

557 aaccc uaccc 2.211 1.35e-02

558 aaccc acccc 2.440 7.34e-03

565 aaccc aaccu 2.440 7.34e-03

567 ccyycch ccucucc 2.775 2.76e-03

572 ccyycch ccugccu 2.250 1.22e-02

583 aaccc aaccu 2.211 1.35e-02

636 ccyycch ccuaccc 2.125 1.68e-02

638 uuccc uaccc 1.826 3.39e-02

822 uuccc guccc 1.881 3.00e-02

823 uuccc ucccc 1.890 2.94e-02

842 uuccc auccc 2.413 7.91e-03

866 aaccc agccc 2.211 1.35e-02

889 aaccc gaccc 2.211 1.35e-02

890 aaccc acccc 2.211 1.35e-02

891 ccccacccc ccccagccc 3.607 1.55e-04

895 aaccc agccc 1.752 3.99e-02

908 ccccacccc ccccucccc 3.574 1.76e-04

909 ccyycch cccuccc 3.137 8.53e-04

910 ccyycch ccucccc 3.175 7.49e-04

911 uuccc cuccc 2.229 1.29e-02

912 uuccc ucccc 2.239 1.26e-02

1032 ccccacccc cccccuccc 3.098 9.74e-04

1033 ccccacccc ccccucccc 3.311 4.65e-04

1034 ccyycch cccuccc 2.700 3.47e-03

1035 ccyycch ccucccc 2.737 3.10e-03

1121 ccyycch cccugcc 1.713 4.34e-02

1122 ccyycch ccugccu 1.725 4.23e-02

1536 ccccacccc ccccaaucc 1.934 2.66e-02

1581 ccccacccc cccccccag 2.951 1.58e-03

1582 ccccacccc ccccccagc 2.951 1.58e-03

1584 ccccacccc ccccagcgc 2.918 1.76e-03

1760 uuccc cuccc 1.734 4.15e-02

1764 uuccc cuccc 1.734 4.15e-02

1770 uuccc ugccc 1.743 4.07e-02

1852 aaccc agccc 1.752 3.99e-02

1855 ccccacccc ccccuuacc 1.902 2.86e-02

1860 uuccc uaccc 1.817 3.46e-02

1885 aaccc aacgc 1.752 3.99e-02

1901 ccyycch ccuucuc 2.637 4.18e-03

1924 ccyycch uccuccc 2.587 4.84e-03

1925 ccyycch ccucccg 2.687 3.60e-03

2025 aaccc caccc 1.752 3.99e-02

2040 aaccc gaccc 1.752 3.99e-02

2041 aaccc acccc 1.752 3.99e-02

2134 aaccc agccc 1.752 3.99e-02

2149 aaccc aaucc 2.211 1.35e-02

2150 uuccc auccc 2.606 4.58e-03

2151 uuccc ucccc 2.459 6.97e-03

2167 uuccc guccc 2.266 1.17e-02

2173 aaccc gaccc 1.752 3.99e-02

2377 aaccc gaccc 1.752 3.99e-02

2394 aaccc caccc 2.440 7.34e-03

2396 ccccacccc cccagcccc 1.934 2.66e-02

2399 aaccc agccc 2.440 7.34e-03

2401 ccccacccc ccccaaucc 2.000 2.27e-02

2405 aaccc aaucc 2.211 1.35e-02

2406 uuccc auccc 2.376 8.75e-03

2455 aaccc agccc 1.752 3.99e-02

2463 aaccc aagcc 1.752 3.99e-02

2464 aaccc agccc 1.752 3.99e-02

2504 aaccc agccc 1.752 3.99e-02

2520 ccccacccc ccucacccc 3.148 8.22e-04

2523 aaccc caccc 2.440 7.34e-03

2524 aaccc acccc 2.440 7.34e-03

2525 ccccacccc cccccagcc 3.082 1.03e-03

2526 ccccacccc ccccagccc 3.262 5.53e-04

2527 ccccacccc cccagcccc 3.033 1.21e-03

2530 aaccc agccc 2.211 1.35e-02

2548 aaccc aaccu 2.440 7.34e-03

2566 aaccc agccc 2.899 1.87e-03

2577 aaccc gaccc 3.018 1.27e-03

2586 aaccc aaccc 3.468 2.62e-04

2587 aaccc acccc 3.018 1.27e-03

2588 ccccacccc ccccagccc 2.361 9.11e-03

2589 ccccacccc cccagcccc 2.131 1.65e-02

2592 aaccc agccc 2.899 1.87e-03

2594 ccccacccc ccccaagcc 2.230 1.29e-02

2598 aaccc aagcc 2.899 1.87e-03

2636 ccyycch acuuccc 2.600 4.66e-03

2637 ccyycch cuucccc 2.562 5.20e-03

2640 ccccacccc cccccuccu 2.082 1.87e-02

2641 ccccacccc ccccuccug 2.164 1.52e-02

2642 ccyycch cccuccu 2.575 5.01e-03

2768 aaccc aagcc 2.440 7.34e-03

2769 aaccc agccc 2.440 7.34e-03

2786 aaccc uaccc 2.440 7.34e-03

2787 aaccc acccc 2.440 7.34e-03

2792 aaccc agccc 2.440 7.34e-03

2829 ccyycch ccucucc 2.500 6.21e-03

2833 ccyycch uccuccc 2.812 2.46e-03

2834 ccyycch ccuccca 3.000 1.35e-03

2835 uuccc cuccc 1.734 4.15e-02

2857 ccyycch ccuccca 2.637 4.18e-03

2907 ccyycch cccaccc 2.363 9.06e-03

Protein: PCBP3(Hs/Mm)

Sequence Position Motif K-mer Z-score P-value

478 uuuycc cucucc 1.703 4.43e-02

2830 uuuycc cucucc 2.176 1.48e-02

Protein: PCBP4(Hs/Mm)

Sequence Position Motif K-mer Z-score P-value

279 auccccc agccccc 2.319 1.02e-02

302 auccccc aucccau 2.389 8.45e-03

556 auccccc auacccc 1.819 3.45e-02

557 auccccc uacccca 1.847 3.24e-02

558 auccccc accccac 1.819 3.45e-02

842 auccccc aucccau 3.083 1.02e-03

866 auccccc agccccc 3.153 8.08e-04

911 auccccc cucccca 2.653 3.99e-03

1532 auccccc agccccc 2.028 2.13e-02

1760 auccccc cucccuc 2.528 5.74e-03

1764 auccccc cucccau 2.431 7.53e-03

1769 auccccc augcccc 2.403 8.13e-03

1770 auccccc ugcccca 2.458 6.99e-03

1926 auccccc cucccgu 2.236 1.27e-02

1955 auccccc agcccgc 1.833 3.34e-02

2150 auccccc auccccu 2.750 2.98e-03

2399 auccccc agcccca 2.306 1.06e-02

2406 auccccc aucccag 2.403 8.13e-03

2504 auccccc agccccu 2.528 5.74e-03

2524 auccccc accccca 2.750 2.98e-03

2530 auccccc agcccca 2.208 1.36e-02

2566 auccccc agccccu 2.042 2.06e-02

2586 auccccc aacccca 2.736 3.11e-03

2592 auccccc agcccca 2.194 1.41e-02

2786 auccccc uacccca 2.500 6.21e-03

2824 auccccc aucuccc 2.792 2.62e-03

2826 auccccc cucccuc 2.944 1.62e-03

2835 auccccc cucccaa 2.944 1.62e-03

2858 auccccc cucccag 1.903 2.85e-02

Protein: PPRC1(Hs/Mm)

Sequence Position Motif K-mer Z-score P-value

532 ssgcgcs cugcgcg 1.705 4.41e-02

534 ssgcgcs gcgcgca 1.744 4.06e-02

Protein: PRR3(Hs/Mm)

Sequence Position Motif K-mer Z-score P-value

966 uauaag cauaaa 2.595 4.73e-03

970 uauaag aaucag 2.393 8.36e-03

Protein: PTB3(Hs/Mm)

Sequence Position Motif K-mer Z-score P-value

44 cuuucu uucucu 1.679 4.66e-02

474 cuuucu cuuucu 2.568 5.11e-03

476 cuuucu uucucu 2.160 1.54e-02

2710 cuuucu ccuucu 1.753 3.98e-02

Protein: PTBP3(Hs/Mm)

Sequence Position Motif K-mer Z-score P-value

33 cuuucu guuuca 1.650 4.95e-02

44 cuuucu uucucu 1.700 4.46e-02

474 cuuucu cuuucu 2.600 4.66e-03

476 cuuucu uucucu 2.188 1.43e-02

2710 cuuucu ccuucu 1.775 3.79e-02

Protein: PUF60(Hs/Mm)

Sequence Position Motif K-mer Z-score P-value

475 ucucu uuucu 1.897 2.89e-02

477 ucucu ucucu 2.328 9.96e-03

479 ucucu ucucc 1.897 2.89e-02

1454 ucucu ucuca 1.897 2.89e-02

1734 ucucu ugucu 1.897 2.89e-02

1736 ucucu ucuau 2.112 1.73e-02

1745 ucucu acucu 2.112 1.73e-02

1747 ucucu ucuca 2.112 1.73e-02

1761 ucucu ucccu 1.897 2.89e-02

2106 ucucu ccucu 1.897 2.89e-02

2108 ucucu ucugu 1.897 2.89e-02

2124 ucucu gcucu 1.897 2.89e-02

2126 ucucu ucugu 1.897 2.89e-02

2801 ucucu ucuca 1.897 2.89e-02

2809 ucucu ucucc 2.276 1.14e-02

2825 ucucu ucucc 2.276 1.14e-02

2827 ucucu ucccu 2.224 1.31e-02

2829 ucucu ccucu 2.224 1.31e-02

2831 ucucu ucucc 2.224 1.31e-02

2842 ucucu ucucc 2.112 1.73e-02

2922 uuuuu uuugu 1.739 4.10e-02

2961 uuuuu uuuuu 3.076 1.05e-03

2975 uuuuu uuuuu 3.151 8.14e-04

2977 uuuuu uuugu 2.908 1.82e-03

Protein: PUM1(Hs/Mm)

Sequence Position Motif K-mer Z-score P-value

2937 uguau uauaa 2.695 3.52e-03

2951 uguau uguaa 2.916 1.77e-03

Protein: QKI(Hs/Mm)

Sequence Position Motif K-mer Z-score P-value

2777 acuaay acuaaa 2.359 9.16e-03

2784 acuaay acuacc 2.283 1.12e-02

Protein: RALY(Hs/Mm)

Sequence Position Motif K-mer Z-score P-value

2921 uuuuuub uuuugug 2.321 1.01e-02

2929 uuuuuub uuguuuc 2.205 1.37e-02

2960 uuuuuub guuuuug 3.241 5.96e-04

2961 uuuuuub uuuuugg 3.259 5.59e-04

2974 uuuuuub guuuuug 3.250 5.77e-04

2975 uuuuu uuuuu 3.243 5.91e-04

2976 uuuuuub uuuuguu 2.929 1.70e-03

2977 uuuuuub uuuguug 3.143 8.36e-04

2978 uuuuu uuguu 2.904 1.84e-03

2981 uuuuu uuguu 2.904 1.84e-03

Protein: RBFOX1(Hs/Mm)

Sequence Position Motif K-mer Z-score P-value

800 wgcaugm agaaugc 2.842 2.24e-03

817 wgcaugm agcaugu 1.947 2.58e-02

871 wgcaugm cccaugc 2.105 1.76e-02

1730 wgcaugm agcaugu 1.763 3.89e-02

1781 wgcaugm ugcaucu 2.171 1.50e-02

Protein: RBFOX2(Hs/Mm)

Sequence Position Motif K-mer Z-score P-value

782 gcaug gcauc 1.649 4.96e-02

792 gcaug gcagg 1.730 4.18e-02

801 gcaug gaaug 2.495 6.30e-03

1782 gcaug gcauc 1.649 4.96e-02

2649 gcaug gcagg 1.730 4.18e-02

2664 gcaug ccaug 1.649 4.96e-02

2669 gcaug ggaug 1.649 4.96e-02

Protein: RBFOX3(Hs/Mm)

Sequence Position Motif K-mer Z-score P-value

782 gcaug gcauc 1.664 4.81e-02

792 gcaug gcagg 1.745 4.05e-02

801 gcaug gaaug 2.518 5.90e-03

1782 gcaug gcauc 1.664 4.81e-02

2649 gcaug gcagg 1.745 4.05e-02

2664 gcaug ccaug 1.664 4.81e-02

2669 gcaug ggaug 1.664 4.81e-02

Protein: RBM15B(Hs/Mm)

Sequence Position Motif K-mer Z-score P-value

2962 uuuuauuu uuuugguu 2.583 4.90e-03

2974 uuuuauuu guuuuugu 2.542 5.51e-03

2975 uuuuauuu uuuuuguu 2.778 2.73e-03

2976 uuuuauuu uuuuguug 2.736 3.11e-03

Protein: RBM22(Hs/Mm)

Sequence Position Motif K-mer Z-score P-value

350 accgg acccg 2.306 1.06e-02

351 accgg cccgg 2.408 8.02e-03

375 accgg acugg 2.827 2.35e-03

1171 accgg accga 2.857 2.14e-03

1175 accgg aacgg 2.765 2.85e-03

1176 accgg acggg 2.765 2.85e-03

1194 accgg acugg 2.765 2.85e-03

1423 accgg gccgg 1.684 4.61e-02

1443 accgg uccgg 1.796 3.62e-02

Protein: RBM23(Hs/Mm)

Sequence Position Motif K-mer Z-score P-value

281 ccccc ccccc 2.385 8.54e-03

291 ccccc ccacc 2.500 6.21e-03

453 ccccc ccacc 1.954 2.54e-02

547 ccccc ccucc 2.285 1.12e-02

633 ccccc ccacc 1.946 2.58e-02

641 ccccc ccacc 1.946 2.58e-02

868 ccccc ccccc 2.862 2.10e-03

869 ccccc ccccc 2.862 2.10e-03

910 ccccc ccucc 2.446 7.22e-03

1032 ccccc ccccc 2.877 2.01e-03

1035 ccccc ccucc 2.815 2.44e-03

1074 ccccc ccucc 1.769 3.84e-02

1181 ccccc ccccc 2.223 1.31e-02

1534 ccccc ccccc 2.608 4.55e-03

1535 ccccc ccccc 2.608 4.55e-03

1581 ccccc ccccc 2.969 1.49e-03

1582 ccccc ccccc 2.969 1.49e-03

1583 ccccc ccccc 2.969 1.49e-03

1724 ccccc ccucc 1.769 3.84e-02

1763 ccccc ccucc 2.369 8.92e-03

1834 ccccc ccccc 2.862 2.10e-03

1854 ccccc ccccc 2.908 1.82e-03

1907 ccccc ccgcc 2.277 1.14e-02

1922 ccccc ccucc 2.815 2.44e-03

1925 ccccc ccucc 2.815 2.44e-03

1941 ccccc ccucc 2.900 1.87e-03

1958 ccccc ccgcc 2.192 1.42e-02

2340 ccccc ccucc 2.285 1.12e-02

2393 ccccc ccacc 1.785 3.71e-02

2525 ccccc ccccc 2.554 5.32e-03

2640 ccccc ccccc 2.723 3.23e-03

2643 ccccc ccucc 2.669 3.80e-03

2834 ccccc ccucc 2.700 3.47e-03

2857 ccccc ccucc 2.438 7.38e-03

2908 ccccc ccacc 1.954 2.54e-02

Protein: RBM24(Hs/Mm)

Sequence Position Motif K-mer Z-score P-value

247 wgwgugd agucugg 2.351 9.36e-03

248 gugug gucug 2.400 8.20e-03

255 wgwgugd ggagugc 2.403 8.13e-03

256 gugug gagug 2.540 5.54e-03

257 wgwgugd agugcgg 2.351 9.36e-03

271 wgwgugd agugucu 2.416 7.85e-03

272 gugug guguc 2.460 6.95e-03

297 ggggg ggggg 2.292 1.10e-02

397 ggggg ggggg 2.677 3.71e-03

831 wgwgugd acugugu 1.883 2.99e-02

833 wgwgugd ugugucu 1.831 3.36e-02

834 gugug guguc 1.820 3.44e-02

853 wgwgugd guuguga 1.831 3.36e-02

977 wgwgugd aguguga 3.130 8.74e-04

978 gugug gugug 2.800 2.56e-03

1001 wgwgugd agcgugg 2.636 4.19e-03

1002 gugug gcgug 2.900 1.87e-03

1558 wgwgugd uauguga 2.740 3.07e-03

1566 wgwgugd ugagugg 2.831 2.32e-03

1567 gugug gagug 1.980 2.39e-02

1568 wgwgugd agugggg 2.558 5.26e-03

1818 wgwgugd ggagugg 2.026 2.14e-02

1819 gugug gagug 2.100 1.79e-02

1820 wgwgugd agugggu 1.987 2.35e-02

1822 wgwgugd ugggugc 1.974 2.42e-02

1823 gugug gggug 2.040 2.07e-02

2373 ggggg ggggg 2.292 1.10e-02

2961 uuuuuuuuu uuuuugguu 2.935 1.67e-03

2962 uuuuuuuuu uuuugguuu 3.177 7.44e-04

2974 uuuuuuuuu guuuuuguu 2.855 2.15e-03

2975 uuuuuuuuu uuuuuguug 2.935 1.67e-03

2976 uuuuuuuuu uuuuguugu 3.177 7.44e-04

2977 uuuuuuuuu uuuguuguu 2.855 2.15e-03

2981 uuuuuuuuu uuguuguuu 2.855 2.15e-03

Protein: RBM25(Hs/Mm)

Sequence Position Motif K-mer Z-score P-value

297 ggggag ggggga 2.103 1.77e-02

298 ggggag ggggau 2.126 1.68e-02

397 ggggag gggggc 2.483 6.51e-03

398 ggggag ggggca 2.448 7.18e-03

2373 ggggag ggggga 2.103 1.77e-02

2374 ggggag ggggac 2.126 1.68e-02

Protein: RBM28(Hs/Mm)

Sequence Position Motif K-mer Z-score P-value

1004 gwguagd guggaga 2.194 1.41e-02

1007 gwguagd gagaagc 2.083 1.86e-02

Protein: RBM38(Hs/Mm)

Sequence Position Motif K-mer Z-score P-value

256 kkguguk gagugcg 1.744 4.06e-02

1565 kkguguk uugagug 1.962 2.49e-02

1567 kkguguk gaguggg 1.936 2.64e-02

Protein: RBM4(Hs/Mm)

Sequence Position Motif K-mer Z-score P-value

534 gcgcgu gcgcgc 2.096 1.80e-02

536 gcgcgu gcgcag 1.822 3.42e-02

1589 gcgcgu gcgcga 1.932 2.67e-02

Protein: RBM41(Hs/Mm)

Sequence Position Motif K-mer Z-score P-value

2875 uacuu uacuu 2.423 7.70e-03

Protein: RBM42(Hs/Mm)

Sequence Position Motif K-mer Z-score P-value

136 aacuamg aaguaac 2.351 9.36e-03

2776 aacuamg gacuaaa 1.689 4.56e-02

2783 aacuamg aacuacc 1.919 2.75e-02

Protein: RBM45(Hs/Mm)

Sequence Position Motif K-mer Z-score P-value

51 gacgamv gacgaga 2.700 3.47e-03

56 gacgamv gaagaga 2.350 9.39e-03

208 cuuac cugac 2.307 1.05e-02

291 acacc ccacc 1.748 4.02e-02

443 acacc ucacc 1.748 4.02e-02

453 acacc ccacc 1.748 4.02e-02

464 acacc acacu 1.748 4.02e-02

556 acacc auacc 1.748 4.02e-02

558 acacc acccc 1.748 4.02e-02

563 acacc acaac 1.748 4.02e-02

586 cuuac cucac 1.970 2.44e-02

629 acacc agacc 1.748 4.02e-02

633 acacc ccacc 1.748 4.02e-02

641 acacc ccacc 1.748 4.02e-02

722 acgac aggac 1.940 2.62e-02

750 acgca acgca 2.157 1.55e-02

1104 acgca acgcu 1.747 4.03e-02

1230 gacgamv caggaca 1.933 2.66e-02

1270 gacgamv gaagaca 3.283 5.14e-04

1349 acacc acacc 2.682 3.66e-03

1367 acacc gcacc 2.215 1.34e-02

1374 acacc ccacc 2.215 1.34e-02

1503 acacc agacc 1.748 4.02e-02

1712 acacc acacc 1.748 4.02e-02

1858 cuuac cuuac 2.327 9.98e-03

1861 acgca accca 2.518 5.90e-03

1886 acgca acgcu 2.819 2.41e-03

2023 cuuac cucac 2.584 4.88e-03

2024 acacc ucacc 1.748 4.02e-02

2041 acacc acccc 2.215 1.34e-02

2061 acacc acaca 1.748 4.02e-02

2063 acacc acauc 1.748 4.02e-02

2098 cuuac cucac 2.386 8.52e-03

2180 cuuac cucac 1.970 2.44e-02

2251 acgca acgcc 1.867 3.10e-02

2254 acacc ccacc 1.748 4.02e-02

2261 acacc ucacc 1.748 4.02e-02

2605 gacgamv gaggacg 1.867 3.10e-02

2606 acgac aggac 2.100 1.79e-02

2608 gacgamv gacgacu 2.167 1.51e-02

2609 acgac acgac 2.600 4.66e-03

2774 acgac augac 2.020 2.17e-02

2784 acgac acuac 2.430 7.55e-03

2802 cuuac cucac 1.941 2.61e-02

2803 acacc ucacc 1.748 4.02e-02

Protein: RBM46(Hs/Mm)

Sequence Position Motif K-mer Z-score P-value

324 rausawd caucaag 1.882 2.99e-02

Protein: RBM4B(Hs/Mm)

Sequence Position Motif K-mer Z-score P-value

125 acggga ucggaa 2.078 1.89e-02

260 acggga gcgggc 2.156 1.55e-02

534 gcgcgg gcgcgc 1.674 4.71e-02

2658 acggga ccgggu 2.688 3.59e-03

Protein: RBM5(Hs/Mm)

Sequence Position Motif K-mer Z-score P-value

1266 garggwr ggaggaa 2.692 3.55e-03

1280 garggwr gacggau 2.103 1.77e-02

Protein: RBM6(Hs/Mm)

Sequence Position Motif K-mer Z-score P-value

281 ccacc ccccc 2.535 5.62e-03

284 ccacc ccauc 2.535 5.62e-03

288 cgucca cgucca 1.797 3.62e-02

291 ccacc ccacc 3.193 7.04e-04

301 cgucca gauccc 2.342 9.59e-03

305 ccacc ccauc 2.754 2.94e-03

314 ccacc ccucc 2.096 1.80e-02

324 hauccar caucaag 2.169 1.50e-02

434 hauccar aaucuau 2.028 2.13e-02

443 ccacc ucacc 2.535 5.62e-03

453 ccacc ccacc 2.974 1.47e-03

459 ccacc caacc 2.535 5.62e-03

462 ccacc ccaca 2.535 5.62e-03

543 ccacc cuacc 2.316 1.03e-02

547 ccacc ccucc 2.316 1.03e-02

550 ccacc ccagc 2.316 1.03e-02

561 ccacc ccaca 2.316 1.03e-02

564 ccacc caacc 2.316 1.03e-02

633 ccacc ccacc 3.412 3.22e-04

637 ccacc cuacc 2.754 2.94e-03

641 ccacc ccacc 3.412 3.22e-04

644 ccacc ccagc 2.754 2.94e-03

698 hauccar acuccau 2.211 1.35e-02

702 hauccar cauccug 2.310 1.04e-02

735 ccacc ccacu 1.658 4.87e-02

748 ccacc ccacg 1.658 4.87e-02

756 ccacc acacc 1.658 4.87e-02

868 ccacc ccccc 1.658 4.87e-02

869 ccacc ccccc 1.658 4.87e-02

893 ccacc ccagc 2.096 1.80e-02

1013 ccacc ccagc 2.096 1.80e-02

1032 ccacc ccccc 1.658 4.87e-02

1035 ccacc ccucc 1.658 4.87e-02

1074 ccacc ccucc 2.096 1.80e-02

1078 ccacc ccagc 2.096 1.80e-02

1082 ccacc ccacg 2.096 1.80e-02

1091 ccacc acacc 2.096 1.80e-02

1151 ccacc ccagc 1.658 4.87e-02

1169 ccacc cuacc 2.096 1.80e-02

1181 ccacc ccccc 1.658 4.87e-02

1184 ccacc ccacu 1.658 4.87e-02

1331 ccacc ccauc 1.658 4.87e-02

1349 ccacc acacc 2.754 2.94e-03

1352 ccacc ccaca 2.754 2.94e-03

1367 ccacc gcacc 2.535 5.62e-03

1374 ccacc ccacc 2.974 1.47e-03

1581 ccacc ccccc 2.096 1.80e-02

1582 ccacc ccccc 2.096 1.80e-02

1583 ccacc ccccc 2.096 1.80e-02

1586 ccacc ccagc 2.096 1.80e-02

1712 ccacc acacc 1.658 4.87e-02

1724 ccacc ccucc 1.658 4.87e-02

1728 ccacc ccagc 1.658 4.87e-02

1854 ccacc ccccc 2.316 1.03e-02

1863 ccacc ccacu 2.316 1.03e-02

1868 ccacc acacc 2.316 1.03e-02

1871 ccacc ccauc 2.316 1.03e-02

1883 ccacc ccaac 2.316 1.03e-02

1898 cgucca aguccu 2.582 4.91e-03

1907 ccacc ccgcc 2.316 1.03e-02

1910 ccacc ccauc 2.096 1.80e-02

1911 cgucca cauccu 3.089 1.00e-03

1922 cgucca ccuccu 3.430 3.02e-04

1925 cgucca ccuccc 3.532 2.06e-04

1938 cgucca uguccu 2.873 2.03e-03

1941 cgucca ccucca 3.215 6.52e-04

1958 ccacc ccgcc 2.096 1.80e-02

1974 hauccar cauccac 1.845 3.25e-02

1977 ccacc ccacc 2.535 5.62e-03

1991 ccacc ccagc 2.754 2.94e-03

2006 ccacc ccacc 2.974 1.47e-03

2009 ccacc ccaac 2.535 5.62e-03

2024 ccacc ucacc 2.096 1.80e-02

2232 ccacc ccagc 2.096 1.80e-02

2254 ccacc ccacc 2.974 1.47e-03

2258 ccacc ccauc 2.096 1.80e-02

2261 ccacc ucacc 2.096 1.80e-02

2352 cgucca ggucca 2.557 5.28e-03

2367 hauccar gcuccag 2.789 2.64e-03

2390 hauccar uuuccac 3.056 1.12e-03

2393 ccacc ccacc 2.535 5.62e-03

2394 hauccar cacccag 3.141 8.42e-04

2397 ccacc ccagc 2.535 5.62e-03

2405 hauccar aauccca 2.831 2.32e-03

2417 ccacc gcacc 2.535 5.62e-03

2420 ccacc ccagc 1.658 4.87e-02

2522 ccacc ucacc 2.096 1.80e-02

2525 ccacc ccccc 1.658 4.87e-02

2528 ccacc ccagc 1.658 4.87e-02

2626 ccacc ccacu 1.658 4.87e-02

2637 cgucca cuuccc 2.873 2.03e-03

2640 ccacc ccccc 1.658 4.87e-02

2643 cgucca ccuccu 2.835 2.29e-03

2661 cgucca ggucca 2.962 1.53e-03

2785 ccacc cuacc 2.096 1.80e-02

2790 ccacc ccagc 2.096 1.80e-02

2803 ccacc ucacc 2.430 7.55e-03

2806 ccacc ccauc 2.430 7.55e-03

2809 cgucca ucucca 2.633 4.23e-03

2816 ccacc ucacc 2.316 1.03e-02

2819 ccacc ccagc 2.316 1.03e-02

2825 cgucca ucuccc 2.797 2.58e-03

2834 cgucca ccuccc 3.405 3.31e-04

2842 cgucca ucucca 3.000 1.35e-03

2857 cgucca ccuccc 2.544 5.48e-03

2898 ccacc ccauc 2.096 1.80e-02

2899 hauccar cauccag 3.183 7.29e-04

2902 ccacc ccagc 2.096 1.80e-02

2908 ccacc ccacc 2.535 5.62e-03

2909 hauccar cacccaa 2.577 4.98e-03

Protein: RBM8A(Hs/Mm)

Sequence Position Motif K-mer Z-score P-value

258 rygcgcb gugcggg 2.176 1.48e-02

260 rygcgcb gcgggca 2.081 1.87e-02

532 rygcgcb cugcgcg 1.878 3.02e-02

534 rygcgcb gcgcgca 2.027 2.13e-02

1587 rygcgcb cagcgcg 2.432 7.51e-03

1589 rygcgcb gcgcgag 2.568 5.11e-03

Protein: RC3H1(Hs/Mm)

Sequence Position Motif K-mer Z-score P-value

1475 cuaug cucug 1.705 4.41e-02

1488 cuaug ccaug 1.705 4.41e-02

1493 cuaug ccaug 2.181 1.46e-02

1557 cuaug cuaug 1.705 4.41e-02

1737 cuaug cuaug 1.705 4.41e-02

2107 cuaug cucug 1.705 4.41e-02

2109 cuaug cugug 1.705 4.41e-02

2125 cuaug cucug 1.705 4.41e-02

2348 cuaug cucug 2.181 1.46e-02

2361 cuaug cuaug 1.705 4.41e-02

2541 cuaug cuaug 1.705 4.41e-02

Protein: SAMD4A(Hs/Mm)

Sequence Position Motif K-mer Z-score P-value

29 gckgghm gcugguu 1.827 3.38e-02

84 gckgghm gcugggc 1.667 4.78e-02

217 gckgghm gcugccc 2.259 1.19e-02

516 gckgghm ccuggac 2.123 1.69e-02

538 gckgghm gcaggcu 2.086 1.85e-02

919 gckgghm gcaggcu 2.667 3.83e-03

938 gckgghm gcuggga 2.358 9.19e-03

1016 gckgghm gcugggc 2.617 4.44e-03

1115 gckgghm gcuggcc 2.037 2.08e-02

1221 gckgghm gcuggac 1.951 2.55e-02

Protein: SART3(Hs/Mm)

Sequence Position Motif K-mer Z-score P-value

2291 araaaam aaaagaa 2.059 1.97e-02

2778 araaaam cuaaaaa 2.078 1.89e-02

Protein: SFPQ(Hs/Mm)

Sequence Position Motif K-mer Z-score P-value

2947 kurrukk guauugu 2.981 1.44e-03

2948 uagug uauug 1.769 3.84e-02

2951 uguaag uguaaa 2.482 6.53e-03

2957 kurrukk cuaguuu 3.111 9.32e-04

2964 kurrukk uugguuu 3.333 4.30e-04

2983 kurrukk guuguuu 2.852 2.17e-03

Protein: SNRNP70(Hs/Mm)

Sequence Position Motif K-mer Z-score P-value

132 rwucaag ggugaag 2.000 2.27e-02

145 rwucaag guccaag 3.233 6.12e-04

169 rwucaag caucaag 3.123 8.95e-04

1662 rwucaag caucagg 2.027 2.13e-02

2367 rwucaag gcuccag 1.836 3.32e-02

2889 rwucaag gaucaug 2.521 5.85e-03

2899 rwucaag cauccag 2.329 9.93e-03

Protein: SNRPA(Hs/Mm)

Sequence Position Motif K-mer Z-score P-value

307 ugcaca aucaca 1.868 3.09e-02

1365 wugcacr cugcacc 2.493 6.33e-03

1366 ugcaca ugcacc 3.447 2.83e-04

1372 wugcacr uuccacc 2.268 1.17e-02

1789 wugcacr aagcaca 2.789 2.64e-03

1790 ugcaca agcaca 2.171 1.50e-02

1809 ugcaca ggcaca 2.079 1.88e-02

2204 ugcaca ugccca 1.947 2.58e-02

2247 wugcacr uggcacg 2.268 1.17e-02

2248 ugcaca ggcacg 2.842 2.24e-03

2477 ugcaca ggcacu 2.750 2.98e-03

Protein: SRSF1(Hs/Mm)

Sequence Position Motif K-mer Z-score P-value

69 gragga ggaggc 1.978 2.40e-02

1044 gragga ggaggc 1.925 2.71e-02

1266 gragga ggagga 3.312 4.63e-04

1269 gragga ggaaga 2.968 1.50e-03

1613 gragga ggagga 2.699 3.48e-03

2217 gragga ggagga 2.387 8.49e-03

2220 gragga ggagga 2.387 8.49e-03

2280 gragga ggagga 3.366 3.81e-04

2283 gragga ggagga 3.366 3.81e-04

2286 gragga ggauga 2.935 1.67e-03

2604 gragga ggagga 2.086 1.85e-02

2607 gragga ggacga 1.796 3.62e-02

2673 gragga ggaggc 2.441 7.32e-03

Protein: SRSF10(Hs/Mm)

Sequence Position Motif K-mer Z-score P-value

53 agagavm cgagaag 2.422 7.72e-03

58 agagavm agagaga 2.867 2.07e-03

60 agagavm agagacc 2.759 2.90e-03

328 cagcag aagcag 2.750 2.98e-03

336 cagcag cugcag 2.675 3.74e-03

380 cagcag gagcag 2.912 1.80e-03

399 cagcag gggcag 3.350 4.04e-04

403 cagcag aggcag 3.212 6.59e-04

406 cagcag cagcag 2.925 1.72e-03

508 cagcag cugcag 2.700 3.47e-03

536 cagcag gcgcag 2.212 1.35e-02

790 cagcag gugcag 2.412 7.93e-03

794 cagcag aggcag 2.800 2.56e-03

816 cagcag cagcau 2.037 2.08e-02

949 cagcag uggcag 2.375 8.77e-03

997 cagcag ccgcag 2.937 1.66e-03

1210 cagcag cggcag 2.262 1.18e-02

1216 cagcag aggcag 3.100 9.68e-04

1237 cagcag gugcag 2.887 1.94e-03

1633 agagavm agagcca 2.434 7.47e-03

1656 agagavm ggagaac 2.012 2.21e-02

2440 cagcag gggcag 3.037 1.19e-03

2451 cagcag gggcag 2.337 9.72e-03

Protein: SRSF11(Hs/Mm)

Sequence Position Motif K-mer Z-score P-value

68 agggg aggag 2.104 1.77e-02

80 agggg agggg 2.755 2.93e-03

92 agggg agagg 2.509 6.05e-03

2752 agggg agagg 2.038 2.08e-02

2754 agggg agggg 2.283 1.12e-02

Protein: SRSF2(Hs/Mm)

Sequence Position Motif K-mer Z-score P-value

52 ggagwd acgaga 2.655 3.97e-03

55 ggagwd agaaga 2.655 3.97e-03

57 ggagwd aagaga 2.655 3.97e-03

68 ggagwd aggagg 2.874 2.03e-03

329 agcagc agcaga 1.966 2.46e-02

337 agcagc ugcagc 1.977 2.40e-02

353 ggagwd cggagc 2.540 5.54e-03

370 ggagwd uggaga 2.839 2.26e-03

378 ggagwd gggagc 2.540 5.54e-03

381 agcagc agcaga 2.864 2.09e-03

400 agcagc ggcagg 3.284 5.12e-04

404 agcagc ggcagc 3.523 2.13e-04

407 agcagc agcagc 3.136 8.56e-04

509 agcagc ugcaga 2.330 9.90e-03

530 agcagc agcugc 2.159 1.54e-02

571 uccag uccug 1.936 2.64e-02

595 agcagc agcagc 1.886 2.96e-02

791 agcagc ugcagg 2.432 7.51e-03

795 agcagc ggcaga 2.330 9.90e-03

814 agcagc accagc 2.159 1.54e-02

983 agcagc agcugc 2.614 4.47e-03

995 agcagc agccgc 2.614 4.47e-03

998 agcagc cgcagc 2.852 2.17e-03

1008 agcagc agaagc 2.614 4.47e-03

1135 ggagwd uggagc 1.782 3.74e-02

1144 ggagwd uggagc 1.782 3.74e-02

1157 ggagwd uggagc 1.782 3.74e-02

1217 agcagc ggcagc 2.784 2.68e-03

1238 agcagc ugcagc 1.886 2.96e-02

1630 ggagwd gggaga 2.839 2.26e-03

1655 ggagwd aggaga 2.126 1.68e-02

1976 uccag uccac 1.936 2.64e-02

1994 uccag gccag 1.936 2.64e-02

2354 uccag uccag 1.936 2.64e-02

2369 uccag uccag 2.845 2.22e-03

2392 uccag uccac 2.391 8.40e-03

2396 uccag cccag 1.936 2.64e-02

2408 uccag cccag 1.936 2.64e-02

2419 agcagc accagc 2.136 1.63e-02

2441 agcagc ggcagc 3.091 9.97e-04

2444 agcagc agcugc 2.727 3.20e-03

2452 agcagc ggcagc 2.625 4.33e-03

2645 uccag uccug 1.936 2.64e-02

2648 uccag ugcag 1.936 2.64e-02

2657 uccag uccgg 1.936 2.64e-02

2663 uccag uccau 1.936 2.64e-02

2672 ggagwd uggagg 1.966 2.46e-02

2746 ggagwd uggaga 2.920 1.75e-03

2749 ggagwd agaaga 2.678 3.70e-03

2754 ggagwd agggga 2.736 3.11e-03

2756 ggagwd gggagg 2.655 3.97e-03

2789 uccag cccag 1.936 2.64e-02

2794 uccag cccag 2.391 8.40e-03

2811 uccag uccag 2.845 2.22e-03

2818 uccag accag 1.936 2.64e-02

2885 uccag accag 2.391 8.40e-03

2897 uccag uccau 1.936 2.64e-02

2901 uccag uccag 2.391 8.40e-03

Protein: SRSF4(Hs/Mm)

Sequence Position Motif K-mer Z-score P-value

28 agcag agcug 1.842 3.27e-02

55 agcag agaag 1.842 3.27e-02

68 agcag aggag 1.842 3.27e-02

77 agcag agcag 2.281 1.13e-02

381 agcag agcag 2.281 1.13e-02

400 agcag ggcag 2.500 6.21e-03

404 agcag ggcag 2.500 6.21e-03

407 agcag agcag 2.281 1.13e-02

509 agcag ugcag 1.842 3.27e-02

521 agcag accag 2.061 1.97e-02

530 agcag agcug 1.842 3.27e-02

791 agcag ugcag 1.842 3.27e-02

795 agcag ggcag 2.061 1.97e-02

814 agcag accag 1.842 3.27e-02

950 agcag ggcag 1.842 3.27e-02

971 agcag aucag 2.061 1.97e-02

974 agcag agaag 2.175 1.48e-02

983 agcag agcug 2.175 1.48e-02

995 agcag agccg 2.228 1.29e-02

998 agcag cgcag 2.175 1.48e-02

1008 agcag agaag 2.061 1.97e-02

1015 agcag agcug 1.842 3.27e-02

1217 agcag ggcag 2.061 1.97e-02

1220 agcag agcug 2.061 1.97e-02

1238 agcag ugcag 1.842 3.27e-02

1241 agcag agcug 1.842 3.27e-02

2297 agcag agccg 1.842 3.27e-02

2419 agcag accag 1.842 3.27e-02

2426 agcag aucag 1.842 3.27e-02

2441 agcag ggcag 2.061 1.97e-02

2444 agcag agcug 2.061 1.97e-02

Protein: SRSF5(Hs/Mm)

Sequence Position Motif K-mer Z-score P-value

217 gcagc gcugc 1.886 2.96e-02

320 gcagc gcauc 2.325 1.00e-02

330 gcagc gcaga 2.544 5.48e-03

338 gcagc gcagc 2.763 2.86e-03

354 gcagc ggagc 2.325 1.00e-02

379 gcagc ggagc 2.105 1.76e-02

382 gcagc gcaga 2.544 5.48e-03

390 gcagc ucagc 2.877 2.01e-03

401 gcagc gcagg 2.877 2.01e-03

405 gcagc gcagc 3.421 3.12e-04

408 gcagc gcagc 3.202 6.82e-04

485 gcagc gcugc 1.886 2.96e-02

500 gcagc gcaac 1.886 2.96e-02

503 gcagc acagc 1.886 2.96e-02

510 gcagc gcaga 2.105 1.76e-02

531 gcagc gcugc 1.886 2.96e-02

593 gcagc ccagc 1.886 2.96e-02

596 gcagc gcagc 2.325 1.00e-02

615 gcagc ggagc 1.886 2.96e-02

792 gcagc gcagg 1.886 2.96e-02

796 gcagc gcaga 1.886 2.96e-02

867 gcccc gcccc 2.652 4.00e-03

876 gcagc gcugc 1.886 2.96e-02

893 gcagc ccagc 1.886 2.96e-02

901 gcagc gaagc 2.219 1.32e-02

904 gcagc gcggc 2.105 1.76e-02

907 gcccc gcccc 2.748 3.00e-03

919 gcagc gcagg 2.219 1.32e-02

923 gcagc gcugc 2.219 1.32e-02

930 gcagc acagc 2.544 5.48e-03

942 gcagc ggagc 2.544 5.48e-03

951 gcagc gcagc 2.325 1.00e-02

984 gcagc gcugc 2.325 1.00e-02

987 gcccc gcgcc 2.043 2.05e-02

996 gcagc gccgc 2.544 5.48e-03

999 gcagc gcagc 2.982 1.43e-03

1009 gcagc gaagc 2.544 5.48e-03

1013 gcagc ccagc 2.325 1.00e-02

1031 gcccc gcccc 2.748 3.00e-03

1071 gcccc gcucc 2.009 2.23e-02

1098 gcagc gcaga 1.886 2.96e-02

1119 gcccc gcccc 2.078 1.89e-02

1136 gcagc ggagc 1.886 2.96e-02

1145 gcagc ggagc 1.886 2.96e-02

1151 gcagc ccagc 1.886 2.96e-02

1158 gcagc ggagc 1.886 2.96e-02

1180 gcccc gcccc 2.078 1.89e-02

1209 gcagc gcggc 1.886 2.96e-02

1212 gcagc gcaga 1.886 2.96e-02

1218 gcagc gcagc 3.202 6.82e-04

1239 gcagc gcagc 2.763 2.86e-03

1251 gcagc gaagc 1.886 2.96e-02

1346 gcagc gcaac 1.886 2.96e-02

1361 gcagc ucagc 1.886 2.96e-02

1364 gcagc gcugc 2.105 1.76e-02

1367 gcagc gcacc 2.105 1.76e-02

1533 gcccc gcccc 2.652 4.00e-03

1580 gcccc gcccc 2.652 4.00e-03

1833 gcccc gcccc 2.896 1.89e-03

1853 gcccc gcccc 2.896 1.89e-03

2176 gcagc ccagc 1.886 2.96e-02

2183 gcagc acagc 1.886 2.96e-02

2186 gcagc gcagc 2.325 1.00e-02

2397 gcagc ccagc 1.886 2.96e-02

2400 gcccc gcccc 2.287 1.11e-02

2417 gcagc gcacc 2.325 1.00e-02

2420 gcagc ccagc 2.544 5.48e-03

2442 gcagc gcagc 3.421 3.12e-04

2445 gcagc gcugc 2.544 5.48e-03

2453 gcagc gcagc 2.763 2.86e-03

2505 gcccc gcccc 2.461 6.93e-03

2531 gcccc gcccc 2.461 6.93e-03

2567 gcccc gcccc 2.078 1.89e-02

2593 gcccc gcccc 2.078 1.89e-02

Protein: SRSF7(Hs/Mm)

Sequence Position Motif K-mer Z-score P-value

2605 acgacg gaggac 3.231 6.17e-04

2608 acgacg gacgac 3.744 9.06e-05

Protein: SRSF8(Hs/Mm)

Sequence Position Motif K-mer Z-score P-value

202 agcagc ugcagc 1.756 3.95e-02

216 agcagc ggcugc 2.451 7.12e-03

337 agcagc ugcagc 1.841 3.28e-02

381 agcagc agcaga 2.537 5.59e-03

400 agcagc ggcagg 3.110 9.35e-04

404 agcagc ggcagc 3.537 2.02e-04

407 agcagc agcagc 3.073 1.06e-03

484 agcagc ugcugc 2.195 1.41e-02

509 agcagc ugcaga 2.793 2.61e-03

530 agcagc agcugc 2.305 1.06e-02

739 agcagc ugcugc 2.159 1.54e-02

791 agcagc ugcagg 2.146 1.59e-02

795 agcagc ggcaga 2.085 1.85e-02

903 agcagc agcggc 2.073 1.91e-02

918 agcagc ggcagg 2.573 5.04e-03

922 agcagc ggcugc 2.561 5.22e-03

983 agcagc agcugc 3.000 1.35e-03

995 agcagc agccgc 2.976 1.46e-03

998 agcagc cgcagc 3.195 6.99e-04

1211 agcagc ggcaga 2.329 9.93e-03

1217 agcagc ggcagc 3.695 1.10e-04

1238 agcagc ugcagc 2.988 1.40e-03

2441 agcagc ggcagc 3.402 3.34e-04

2444 agcagc agcugc 3.110 9.35e-04

2452 agcagc ggcagc 2.951 1.58e-03

Protein: SRSF9(Hs/Mm)

Sequence Position Motif K-mer Z-score P-value

38 akgavmr aggaacu 2.123 1.69e-02

52 akgavmr acgagaa 2.046 2.04e-02

106 gggaa gggga 2.596 4.72e-03

107 gggaa gggaa 3.035 1.20e-03

108 gggaa ggaaa 2.711 3.35e-03

126 gggaa cggaa 2.711 3.35e-03

127 gggaa ggaaa 2.711 3.35e-03

133 gggaa gugaa 1.939 2.63e-02

186 gggaa ggcaa 2.377 8.73e-03

195 gggaa gggaa 2.377 8.73e-03

196 gggaa ggaaa 1.939 2.63e-02

251 aggag uggag 2.307 1.05e-02

254 aggag aggag 2.505 6.12e-03

353 aggag cggag 2.050 2.02e-02

370 aggag uggag 2.188 1.43e-02

378 aggag gggag 2.208 1.36e-02

675 gggaa gggca 1.939 2.63e-02

676 gggaa ggcaa 1.939 2.63e-02

694 gggaa gggga 1.939 2.63e-02

695 gggaa gggac 1.939 2.63e-02

799 gggaa gagaa 1.939 2.63e-02

1135 aggag uggag 2.218 1.33e-02

1144 aggag uggag 2.218 1.33e-02

1157 aggag uggag 2.218 1.33e-02

1250 kgrwgsm ggaagcc 2.479 6.59e-03

1265 kgrwgsm uggagga 3.127 8.83e-04

1268 aggag aggaa 2.297 1.08e-02

1279 kgrwgsm ggacgga 2.958 1.55e-03

1612 aggag uggag 2.644 4.10e-03

1615 aggag aggau 2.554 5.32e-03

1616 kgrwgsm ggaugga 1.761 3.91e-02

1630 aggag gggag 3.119 9.07e-04

1655 aggag aggag 1.703 4.43e-02

2216 kgrwgsm uggagga 2.690 3.57e-03

2219 kgrwgsm aggagga 2.648 4.05e-03

2222 aggag aggau 2.297 1.08e-02

2279 aggag uggag 2.386 8.52e-03

2282 akgavmr aggagga 2.892 1.91e-03

2285 aggag aggau 2.772 2.79e-03

2286 kgrwgsm ggaugaa 2.254 1.21e-02

2668 gggaa gggau 2.158 1.55e-02

Protein: TAF15(Hs/Mm)

Sequence Position Motif K-mer Z-score P-value

294 ggggggg ccugggg 1.894 2.91e-02

295 ggggggg cuggggg 1.953 2.54e-02

395 ggggggg ccggggg 1.824 3.41e-02

788 gggua gggug 1.800 3.59e-02

1823 gggua gggug 2.276 1.14e-02

1845 gggua gggga 1.800 3.59e-02

1846 gggua gggaa 1.800 3.59e-02

2370 ggggggg ccagggg 1.894 2.91e-02

2371 ggggggg caggggg 1.953 2.54e-02

2451 gggua gggca 1.800 3.59e-02

2660 gggua ggguc 1.800 3.59e-02

2679 ggggggg gcugggg 2.541 5.53e-03

2682 gggua gggga 1.800 3.59e-02

2700 ggggggg ccugggg 1.965 2.47e-02

2752 ggggggg agagggg 2.753 2.95e-03

Protein: TARDBP(Hs/Mm)

Sequence Position Motif K-mer Z-score P-value

801 guaug gaaug 2.459 6.97e-03

Protein: TIA1(Hs/Mm)

Sequence Position Motif K-mer Z-score P-value

2920 uuuuu cuuuu 2.091 1.83e-02

2921 uuuuubk uuuugug 2.225 1.30e-02

2929 uuuuubk uuguuuc 2.039 2.07e-02

2930 uuuuu uguuu 2.091 1.83e-02

2960 uuuuubk guuuuug 3.373 3.72e-04

2961 uuuuubk uuuuugg 3.471 2.59e-04

2962 uuuuu uuuug 2.841 2.25e-03

2974 uuuuubk guuuuug 3.373 3.72e-04

2975 uuuuubk uuuuugu 3.363 3.85e-04

2976 uuuuubk uuuuguu 2.980 1.44e-03

2977 uuuuubk uuuguug 3.186 7.21e-04

Protein: TRA2A(Hs/Mm)

Sequence Position Motif K-mer Z-score P-value

2288 cugaaga augaaaa 2.104 1.77e-02

2293 cugaaga aagaagc 2.015 2.20e-02

Protein: TRNAU1AP(Hs/Mm)

Sequence Position Motif K-mer Z-score P-value

2961 uuuuauu uuuuugg 2.605 4.59e-03

2962 uuuuauu uuuuggu 2.523 5.82e-03

2975 uuuuauu uuuuugu 2.791 2.63e-03

2976 uuuuauu uuuuguu 2.791 2.63e-03

Protein: U2AF2(Hs/Mm)

Sequence Position Motif K-mer Z-score P-value

2929 uuuuuyc uuguuuc 2.486 6.46e-03

2930 uuuuuyc uguuucc 2.371 8.87e-03

2960 uuuuuyc guuuuug 2.524 5.80e-03

2961 uuuuuyc uuuuugg 2.514 5.97e-03

2974 uuuuuyc guuuuug 2.524 5.80e-03

2975 uuuuuyc uuuuugu 2.562 5.20e-03

2976 uuuuuyc uuuuguu 2.438 7.38e-03

Protein: UNK(Hs/Mm)

Sequence Position Motif K-mer Z-score P-value

1597 uauaga aauagc 2.183 1.45e-02

2956 auuaguu acuaguu 2.197 1.40e-02

Protein: YBX1(Hs/Mm)

Sequence Position Motif K-mer Z-score P-value

149 aacaucd aagauca 2.286 1.11e-02

167 aacaucd aacauca 2.740 3.07e-03

304 aacaucd cccauca 3.026 1.24e-03

319 aacaucd ugcauca 3.117 9.14e-04

322 aacaucd aucauca 3.571 1.78e-04

439 aacaucd aucauca 3.104 9.55e-04

583 aacaucd aaccuca 3.234 6.10e-04

592 aacaucd accagca 2.506 6.11e-03

1348 aacaucd aacacca 1.753 3.98e-02

1600 aacaucd agcaaca 2.403 8.13e-03

1660 aacaucd aacauca 2.325 1.00e-02

1687 aacaucd aucaucg 2.468 6.79e-03

1711 aacaucd gacacca 2.286 1.11e-02

1867 aacaucd uacacca 2.403 8.13e-03

1870 aacaucd accaucu 2.662 3.88e-03

1882 aacaucd accaacg 2.299 1.08e-02

2062 aacaucd cacauca 1.805 3.55e-02

2257 aacaucd cccauca 2.195 1.41e-02

2260 aacaucd aucacca 2.221 1.32e-02

2805 aacaucd accaucu 2.896 1.89e-03

2818 aacaucd accagca 2.662 3.88e-03

2821 aacaucd agcaucu 2.792 2.62e-03

Protein: YBX2(Hs/Mm)

Sequence Position Motif K-mer Z-score P-value

149 aacawcd aagauca 1.784 3.72e-02

167 aacawcd aacauca 2.162 1.53e-02

322 aacawcd aucauca 2.041 2.06e-02

562 aacawcd cacaacc 2.162 1.53e-02

565 aacawcd aaccucu 2.676 3.73e-03

583 aacawcd aaccuca 3.068 1.08e-03

588 aacawcd cacaacc 2.892 1.91e-03

592 aacawcd accagca 2.473 6.70e-03

1597 aacawcd aauagca 2.892 1.91e-03

1600 aacawcd agcaaca 3.203 6.80e-04

1603 aacawcd aacaaga 3.122 8.98e-04

1870 aacawcd accaucu 2.730 3.17e-03

1882 aacawcd accaacg 2.284 1.12e-02

2780 aacawcd aaaaacu 2.338 9.69e-03

2783 aacawcd aacuacc 2.162 1.53e-02

2805 aacawcd accaucu 3.122 8.98e-04

2818 aacawcd accagca 2.081 1.87e-02

2821 aacawcd agcaucu 2.311 1.04e-02

Protein: ZC3H10(Hs/Mm)

Sequence Position Motif K-mer Z-score P-value

382 ssagcgm gcagagc 2.164 1.52e-02

408 ssagcgm gcagcgu 2.671 3.78e-03

531 ssagcgm gcugcgc 2.233 1.28e-02

876 ssagcgm gcugcgg 2.658 3.93e-03

893 ssagcgm ccagccc 2.233 1.28e-02

901 ssagcgm gaagcgg 2.356 9.24e-03

984 ssagcgm gcugcgc 1.767 3.86e-02

999 ssagcgm gcagcgu 1.932 2.67e-02

1255 ssagcgm ccagugc 2.603 4.62e-03

2420 ssagcgm ccagcga 2.616 4.45e-03

2453 ssagcgm gcagccc 2.808 2.49e-03

2467 ssagcgm ccagggc 2.644 4.10e-03

2473 ssagcgm ccagggc 2.151 1.57e-02

Protein: ZC3H14(Hs/Mm)

Sequence Position Motif K-mer Z-score P-value

2928 uuuduuu cuuguuu 1.753 3.98e-02

2963 uuuduuu uuugguu 3.299 4.85e-04

2964 uuuduuu uugguuu 3.278 5.23e-04

2975 uuuduuu uuuuugu 3.041 1.18e-03

2976 uuuduuu uuuuguu 3.155 8.02e-04

2977 uuuduuu uuuguug 3.165 7.75e-04

2983 uuuduuu guuguuu 3.206 6.73e-04

Protein: ZFP36(Hs/Mm)

Sequence Position Motif K-mer Z-score P-value

2948 uauuu uauug 2.284 1.12e-02

2958 uauuu uaguu 2.181 1.46e-02

2961 uauuu uuuuu 2.284 1.12e-02

2975 uauuu uuuuu 1.888 2.95e-02

Protein: ZNF326(Hs/Mm)

Sequence Position Motif K-mer Z-score P-value

2390 auucc uuucc 1.667 4.78e-02

2405 auucc aaucc 1.667 4.78e-02

2406 auucc auccc 1.667 4.78e-02

Protein: ZNF638(Hs/Mm)

Sequence Position Motif K-mer Z-score P-value

2078 bguusku uguucgg 2.723 3.23e-03

2093 bguusku uguugcu 2.569 5.10e-03

2962 bguusku uuuuggu 3.754 8.70e-05

2976 bguusku uuuuguu 3.400 3.37e-04

2979 bguusku uguuguu 4.077 2.28e-05

2982 bguusku uguuguu 4.077 2.28e-05
