# Supplementary material for: CSF3R-AS promotes hepatocellular carcinoma progression and sorafenib resistance through the CSF3R/JAK2/STAT3 positive feedback loop
Source: Cell Death Dis. 2025 Mar 28;16(1):217. doi: 10.1038/s41419-025-07558-4 (PMC11953311; doi:10.1038/s41419-025-07558-4)
Supplement: Supplementary file 12 — Supplementary figure and table legends [file 41419_2025_7558_MOESM12_ESM.docx]

**Supplementary Figure and Table Legends**

**Figure S1. Overexpression of CSF3R-AS promoted the proliferation of HCC.** (A-B) The results of EdU. (C-D) The results of CCK8. (E-G) The results of clone formation. (* represents P < 0.05, ** represents P < 0.01, *** represents P < 0.001)

**Figure S2.** **Overexpression of CSF3R-AS promoted invasion and angiogenesis, and suppressed apoptosis of HCC.** (A) The results of apoptosis. (B) The results of invasion. (C) The results of angiogenesis. (D-F) The quantitative statistics of Figure S2A-C. (** represents P < 0.01, *** represents P < 0.001, **** represents P < 0.0001)

**Figure S3. G-CSF promoted the progression of HCC.** (A-B) G-CSF promoted the proliferation of HCC. (C) G-CSF suppressed apoptosis of HCC. (D) G-CSF promoted invasion of HCC. (E-H) The quantitative statistics of Figure S3A-D. (I-J) G-CSF activated JAK2/STAT3 signaling pathway and downstream target genes. (* represents P < 0.05, ** represents P < 0.01, *** represents P < 0.001, **** represents P < 0.0001)

**Figure S4. Overexpression of CSF3R rescued the influence of CSF3R-AS on HCC proliferation, apoptosis, invasion and angiogenesis.** (A-C) CSF3R can rescue the influence of CSF3R-AS on HCC proliferation. (D) CSF3R can rescue the influence of CSF3R-AS on HCC apoptosis. (E) CSF3R can rescue the influence of CSF3R-AS on HCC invasion. (F) CSF3R can rescue the influence of CSF3R-AS on HCC angiogenesis. (G-K) The quantitative statistics of Figure S4A-F. (* represents P < 0.05, ** represents P < 0.01, *** represents P < 0.001)

**Figure S5. Knock down of CSF3R rescued the influence of CSF3R-AS on HCC proliferation, apoptosis, invasion and angiogenesis.** (A-C) CSF3R can rescue the influence of CSF3R-AS on HCC proliferation. (D) CSF3R can rescue the influence of CSF3R-AS on HCC apoptosis. (E) CSF3R can rescue the influence of CSF3R-AS on HCC invasion. (F) CSF3R can rescue the influence of CSF3R-AS on HCC angiogenesis. (G-K) The quantitative statistics of Figure S5A-F. (* represents P < 0.05, ** represents P < 0.01, *** represents P < 0.001, **** represents P < 0.0001)

**Table S1. The correlation between CSF3R-AS expression and HCC patients' pathological characteristics.**

**Table S2. Univariate analysis of HCC patients' OS**

**Table S3. Multivariate analysis of HCC patients' OS**

**Table S4. Primer sequences**

**Table S5. RBPs of CSF3R-AS**

**Table S6. RBPs of CSF3R**
