# Supplementary material for: CSF3R-AS promotes hepatocellular carcinoma progression and sorafenib resistance through the CSF3R/JAK2/STAT3 positive feedback loop
Source: Cell Death Dis. 2025 Mar 28;16(1):217. doi: 10.1038/s41419-025-07558-4 (PMC11953311; doi:10.1038/s41419-025-07558-4)

Figure3E

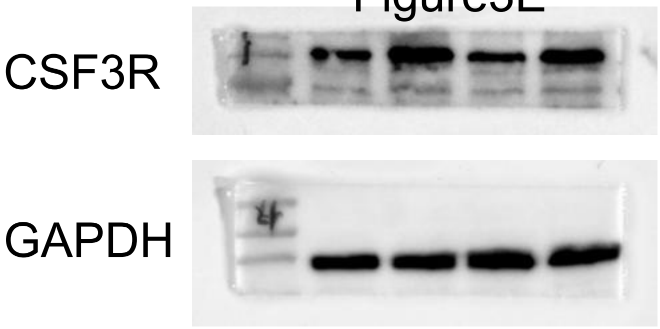

Figure3F

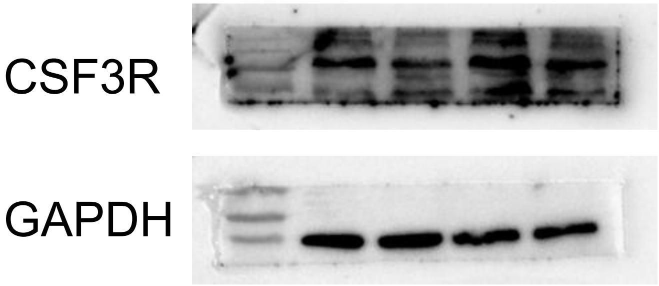

Figure3O

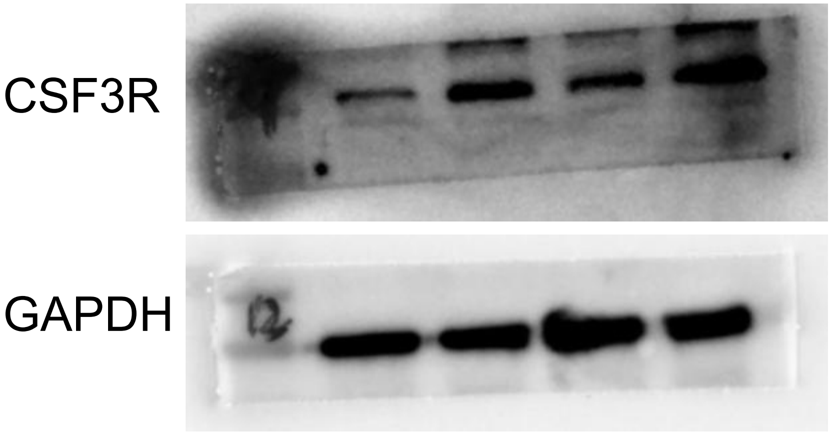

Figure3P

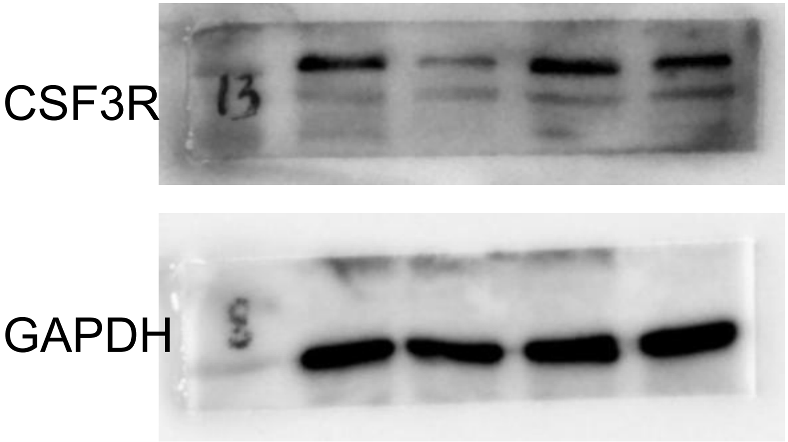

Figure 3R

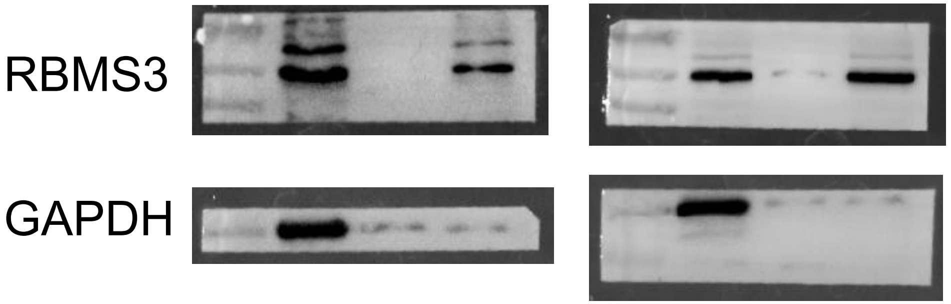

Figure 3S

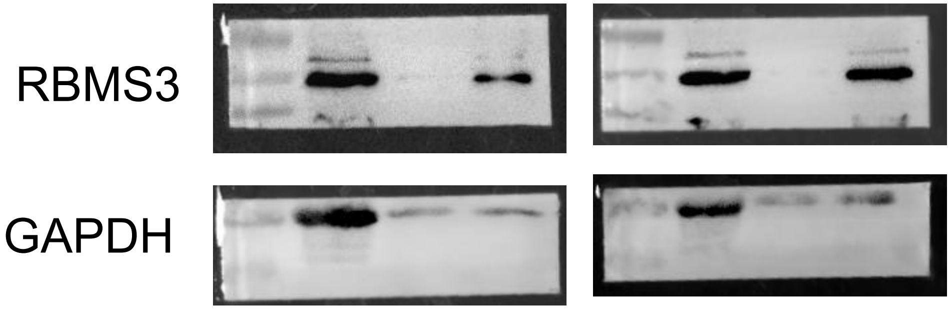

Figure 3U

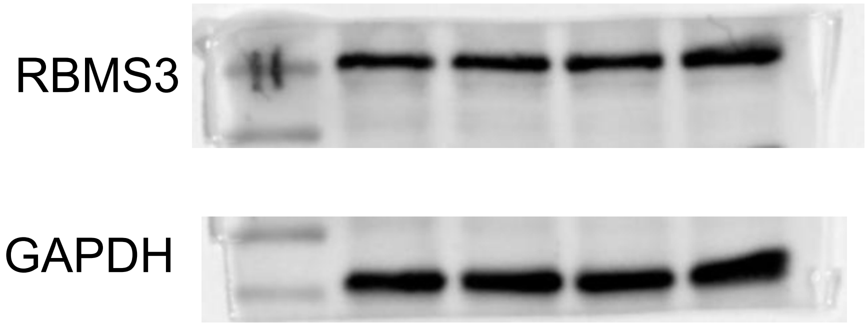

Figure 4B

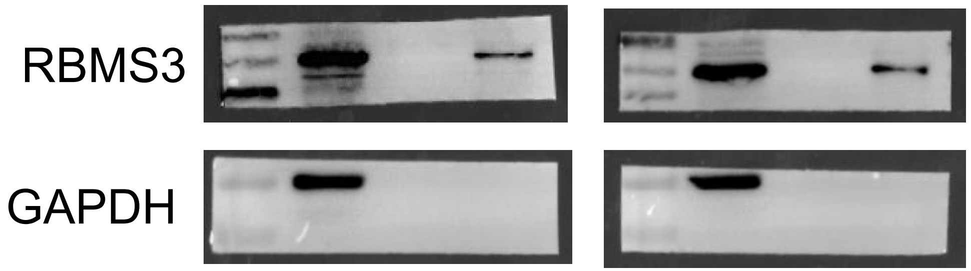

Figure 4C

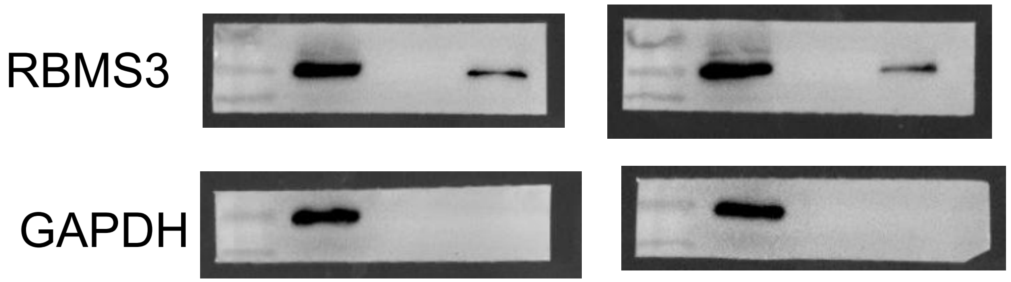

Figure 5A

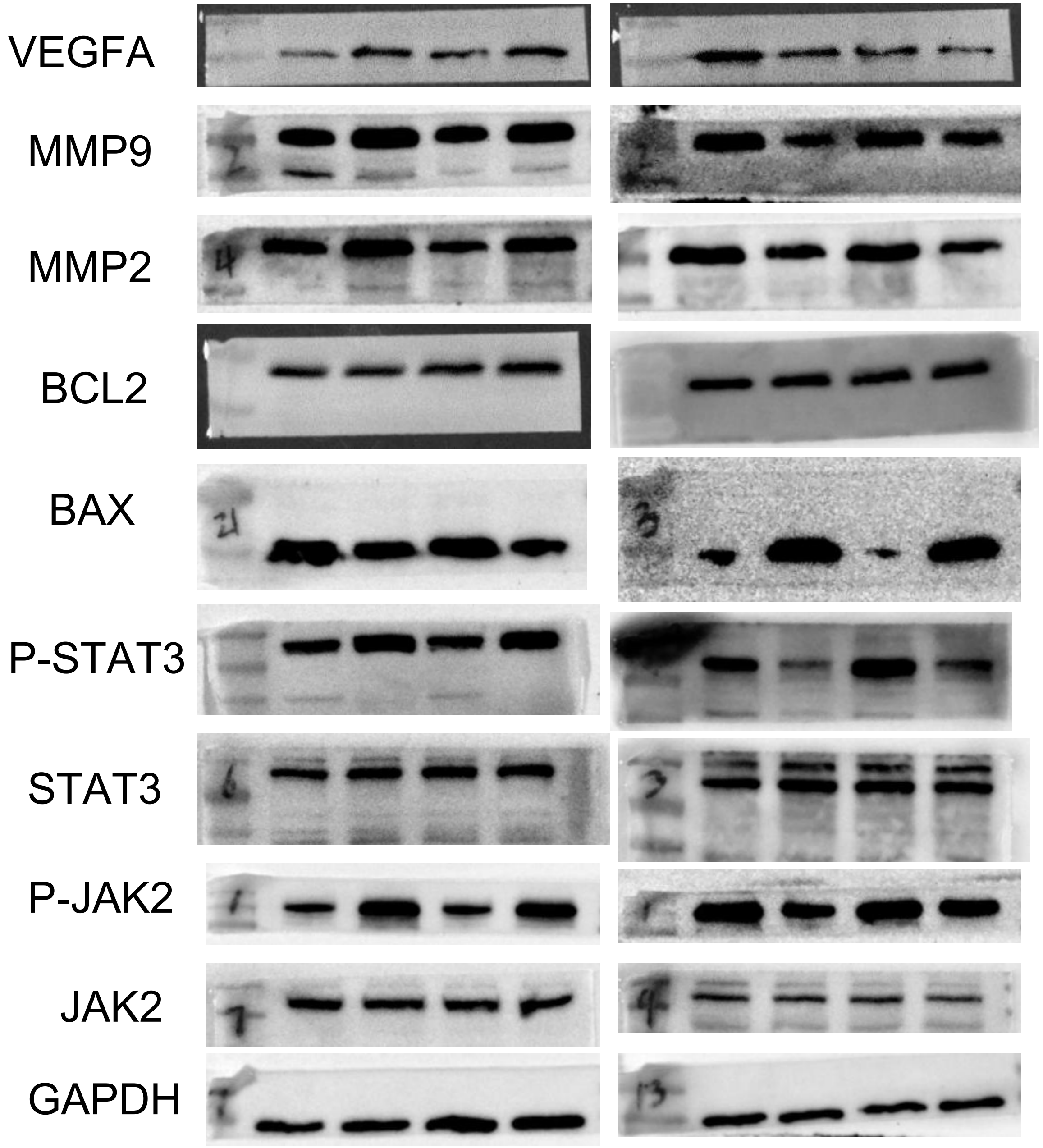

Figure 5D

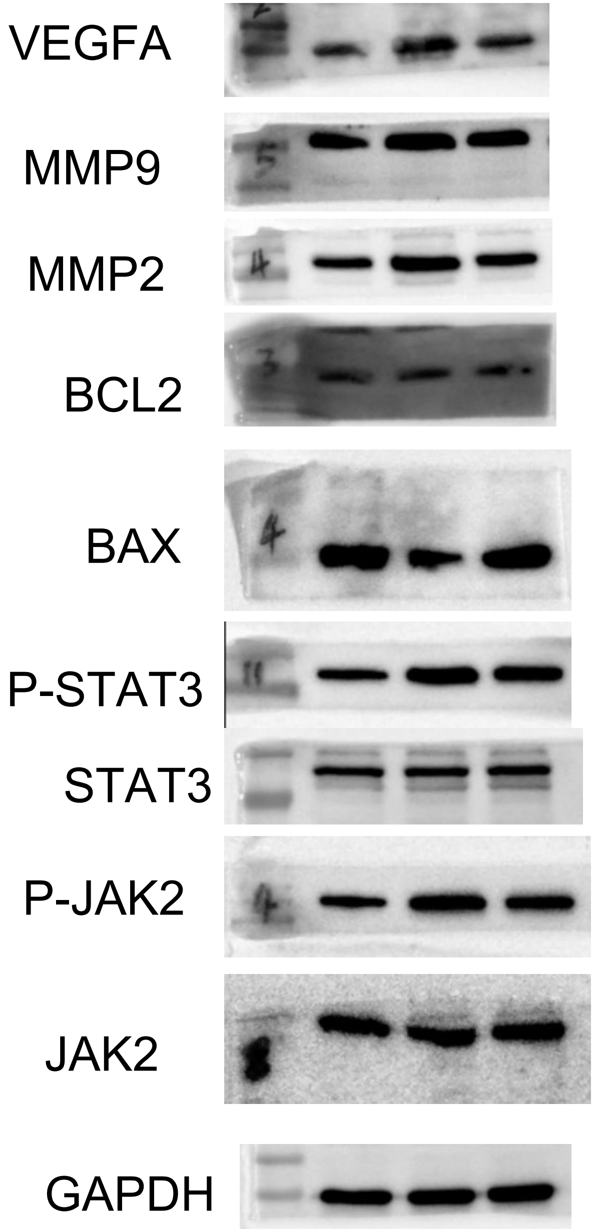

Figure 5E

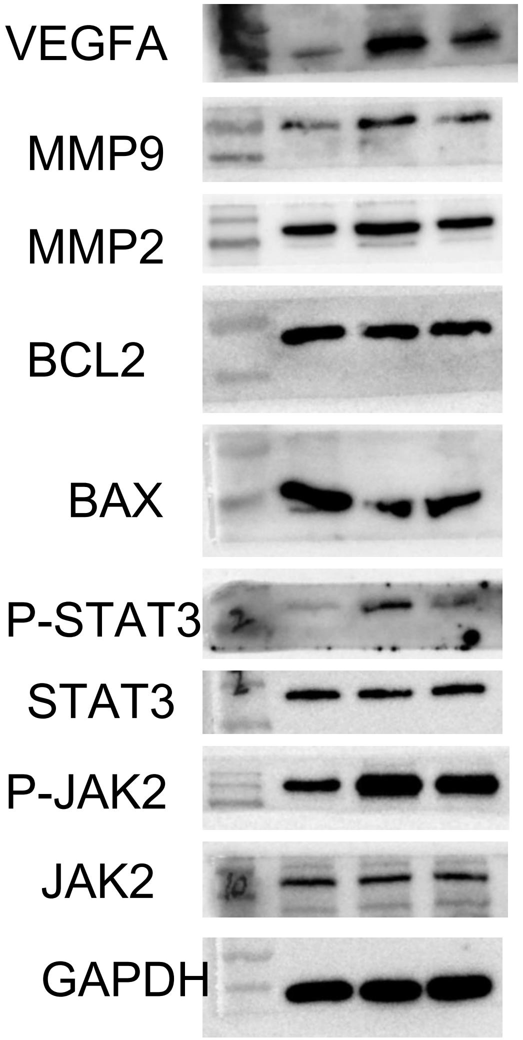

Figure 5F

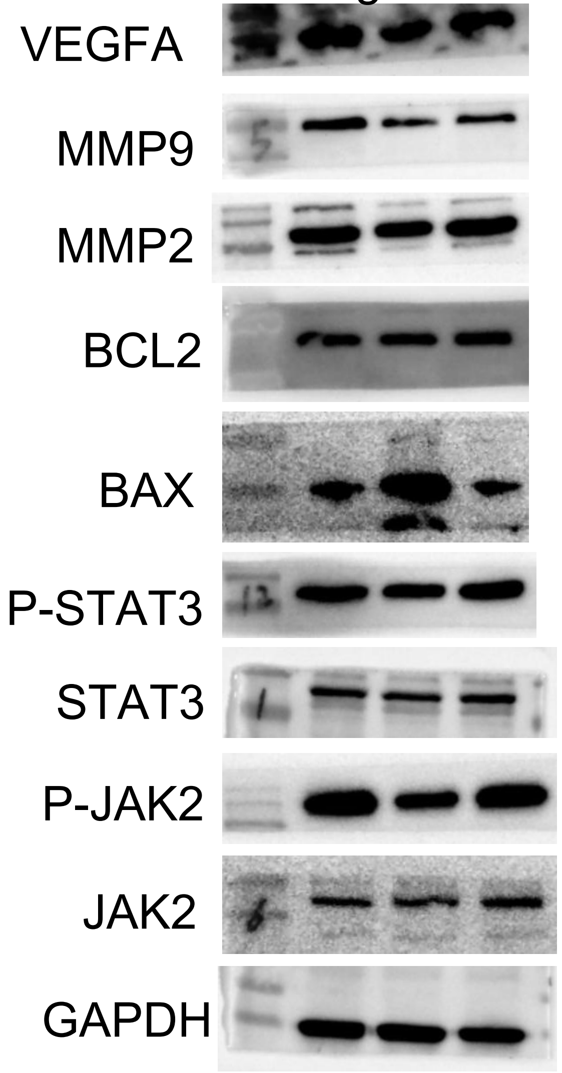

Figure 5G

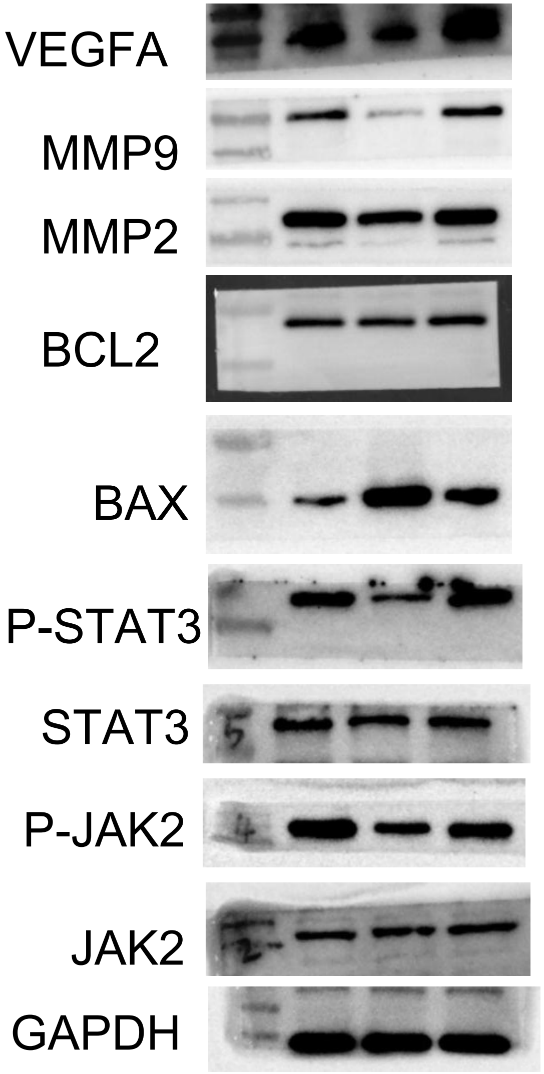

Figure 5Q

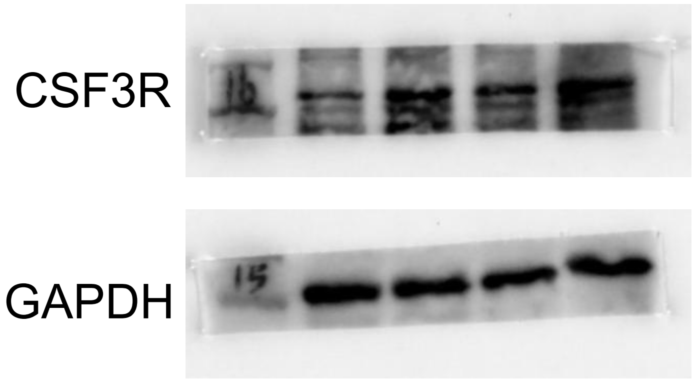

Figure 6E

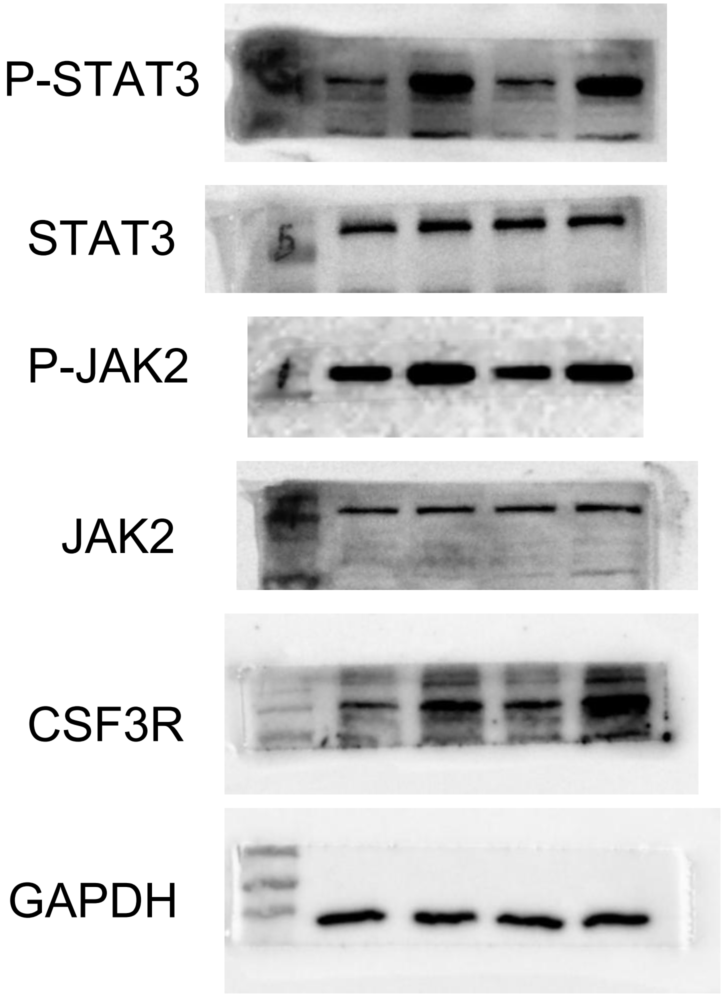

Figure 6F

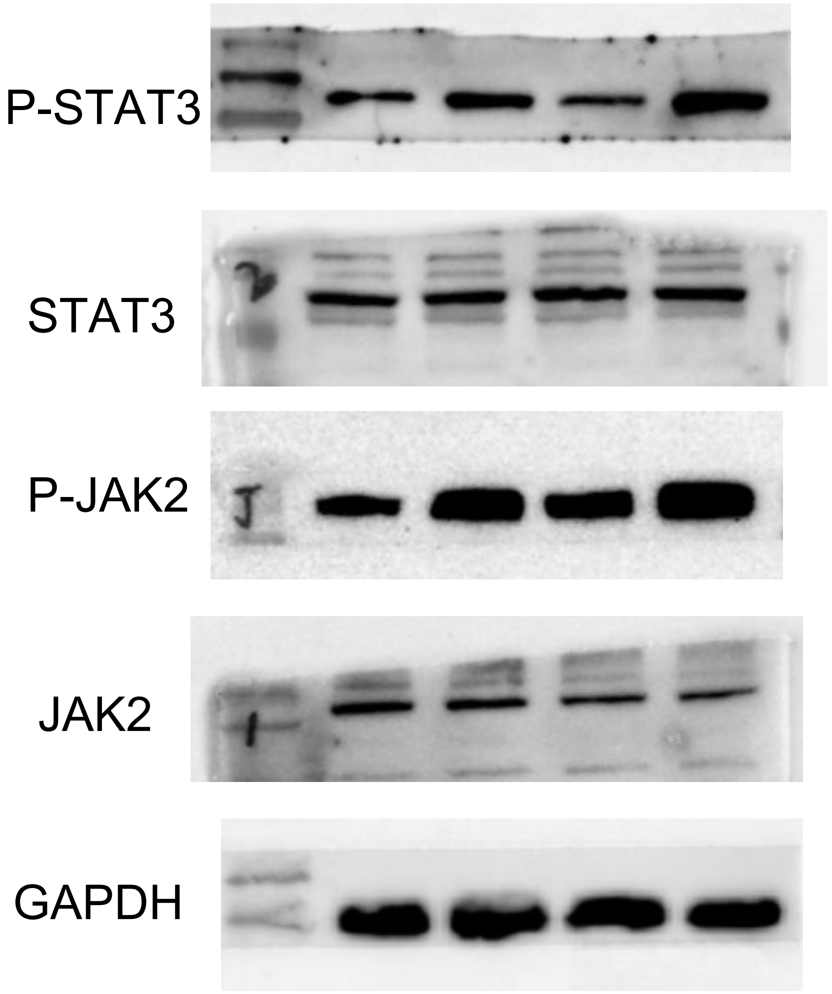

Supplement: Supplementary file 13 — Uncropped original western blots [file 41419_2025_7558_MOESM13_ESM.pdf]
